# Chemoselectivity in Gold(I)-Catalyzed Propargyl Ester Reactions: Insights From DFT Calculations

**DOI:** 10.3389/fchem.2019.00609

**Published:** 2019-09-06

**Authors:** Qing Sun, Pan Hong, Dongdong Wei, Anan Wu, Kai Tan, Xin Lu

**Affiliations:** State Key Laboratory of Physical Chemistry of Solid Surfaces, Fujian Provincial Key Laboratory for Theoretical and Computational Chemistry, Department of Chemistry, College of Chemistry and Chemical Engineering, Xiamen University, Xiamen, China

**Keywords:** homogeneous gold catalysis, chemoselectivity, alkynes, DFT, 1,2,3-triazole

## Abstract

Au-catalyzed propargyl ester reactions have been investigated by a comprehensive density functional theory (DFT) study. Our calculations explain the experimental observed chemoselectivity of Au-catalyzed propargyl ester reactions very well by considering all possible pathways both in the absence and presence of 1,2,3-triazole (TA). The “X-factor” of TA is disclosed to have triple effects on this reaction. First of all, it can stabilize and prevent rapid decomposition of the Au catalyst. Secondly, the existence of TA promotes the nucleophilic attack and alters the chemoselectivity of this reaction. Moreover, TA acts as a “relay” to promote the proton transfer.

## Introduction

Homogeneous gold catalysis has been proven a powerful tool with its extensive applications in modern organic synthesis over the past decades (Hashmi and Hutchings, 2006; Gorin and Toste, 2007; Hashmi, 2007, 2010, 2014; Corma et al., 2011; Rudolph and Hashmi, 2012; Obradors and Echavarren, 2014a,b; Hopkinson et al., 2016). Of particular importance is the selective activation of alkynes, allenes, and alkenes by homogeneous gold catalysis to produce chemically interesting intermediates (Hashmi, 2003; Jiménez-Núñez and Echavarren, 2008; Abu Sohel and Liu, 2009; Krause and Winter, 2011; Ohno, 2013; Zhang, 2014; Dorel and Echavarren, 2015). It is currently accepted that the cationic gold(I) acts as a soft π-Lewis acid and the carbon-carbon multiple bond is activated via a complex of the alkynes/allenes/alkenes-coordinated Au^+^ (Hashmi, 2003; Fürstner and Davies, 2007). The active catalysts employed in the activation of alkynes are generally in the form of [L-Au]^+^ (Fürstner and Davies, 2007; Hashmi, 2007; Shapiro and Toste, 2008). Various experiments have demonstrated that the ligands play a crucial role in the reactivity of the cationic gold catalysis (Gorin et al., 2008; Wang et al., 2012a; Ding et al., 2016; Ebule et al., 2016). Among the gold catalysts reported, the phosphine ligands (PR_3_) have taken a prominent place. However, it surfers from the rapid decomposition, resulting in poor reactivity (Chen et al., 2010; Wang et al., 2012b). Recent developments in the N-heterocyclic carbene (NHC) derivatives have significantly expanded the scope of ligands by improving thermal and substrate stability in addition to good reactivity (de Frémont et al., 2005; Marion et al., 2006; Diez-Gonzalez and Nolan, 2008; Diez-Gonzalez et al., 2009; Ramón et al., 2010).

**Scheme 1:**
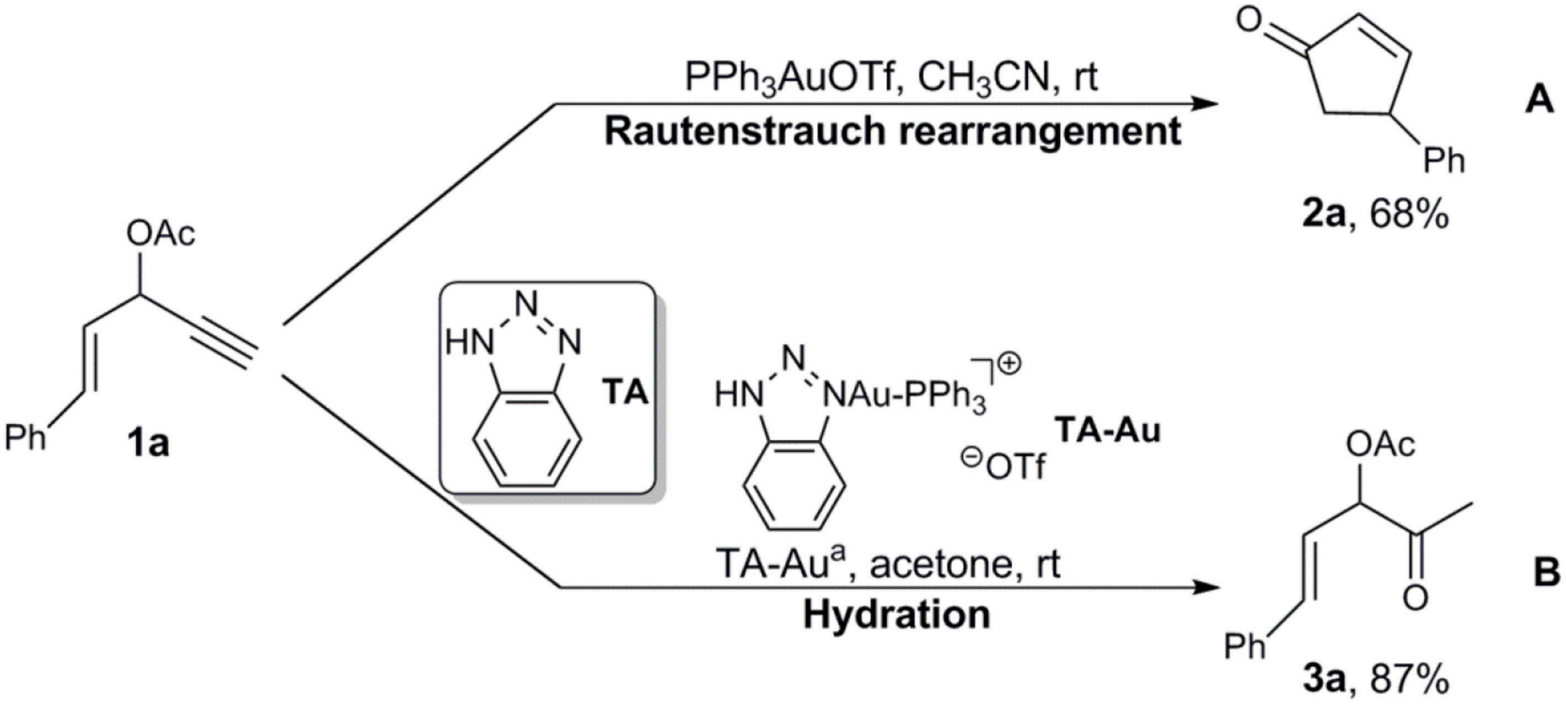
Au-catalyzed propargyl ester reactions reported by **(A)** Toste and **(B)** Shi groups.

Interestingly, Shi et al. developed 1,2,3-triazole (TA)-bound gold complexes as an effective catalysts (denoted as TA-Au) toward the alkynes activation (Chen et al., 2008; Liu et al., 2008; Sengupta et al., 2008; Duan et al., 2009a,b; Yan et al., 2010). This class of Au-catalysts exhibited better performance along with much lower overall costs in catalyzing the transformations of various alkynes, in comparison with the expensive NHC-Au catalysts (Duan et al., 2009a; Chen et al., 2010; Wang et al., 2010, 2011a,b,c; Hosseyni et al., 2015). More fascinating is the TA-Au catalysts even led to unique chemoselectivity. For instance, it was found that the propargyl ester underwent Rautenstrauch rearrangement to produce cyclopentenones with use of a conventional gold catalyst PPh_3_AuOTf (Scheme 1A; Shi et al., 2005), but was hydrated to form α-acetoxy ketone with use of a TA-Au catalyst (Scheme 1B; Wang et al., 2012b). The underlying mechanism of such unique effects of TA in gold catalysis remains unknown as an “X-factor” (Chen et al., 2010), and deserves further in-depth exploration. Herein, we present our theoretical work on the mechanism of the aforementioned Au-catalyzed transformations of propargyl ester in the absence/presence of TA, aiming to the unique role of TA.

## Computational details

All calculations were carried out with the Gaussian 09 program (Frisch et al., 2013). The geometries of all the species were fully optimized by using the M06 functional (Zhao and Truhlar, 2008) with the ultrafine integration grid. The 6-31G(d,p) (Ditchfield et al., 1971; Hehre et al., 1972; Hariharan and Pople, 1973, 1974) basis set was employed for C, H, O, N, P, and the Stuttgart/Dresden small-core RECP (relativistic effective core potential) plus valence double-basis set (SDD) (Andrae et al., 1990) was applied for Au. This combination of functional and basis sets has been frequently used in the mechanistic investigations on Au-catalyzed organic transformations (Shu et al., 2015; Shen et al., 2017a,b). Frequency calculations at the same level were performed to confirm each stationary point to be either a local minimum or a transition state (TS). All transition states were verified by using the intrinsic reaction coordinate (IRC) (Gonzalez and Schlegel, 1990) calculations. Gibbs free energies were obtained with frequency calculations on the optimized structures in acetone at standard condition, given in unit of kcal/mol. The solvent effects of acetone (ε = 20.493) were taken in account by using the SMD-flavor (Marenich et al., 2009) of self-consistent reaction field (SCRF) theory. The atomic charges were analyzed by natural bond orbital (NBO) theory (Foster and Weinhold, 1980; Carpenter and Weinhold, 1988; Reed et al., 1988). All 3D structures were generated by the CYLview (Legault, 2009).

It should be mentioned that theoretical modeling of reactions in wet chemistry requires not only the use of the SCRF-based solvent-effect model but also the explicit involvement of several H_2_O molecules as close environment. For example, the explicit involvement of a water trimer cluster led to better results in the theoretical simulation of organometallic reactions (Kovács et al., 2005; Shi et al., 2007). Accordingly, we took similarly a water trimer cluster model to simulate the hydration reactions (Figures S1, S2). To save computational costs, the bulky triphenylphosphine (PPh_3_) ligand used in experiments was simplified as trimethylphosphine (PMe_3_) and such simplification was been validated by our current results (Scheme S1) and by previous theoretical work on Au-catalyzed reactions (Shi et al., 2007; Faza and López, 2013; Jin et al., 2016).

## Results and Discussion

**Scheme 2:**
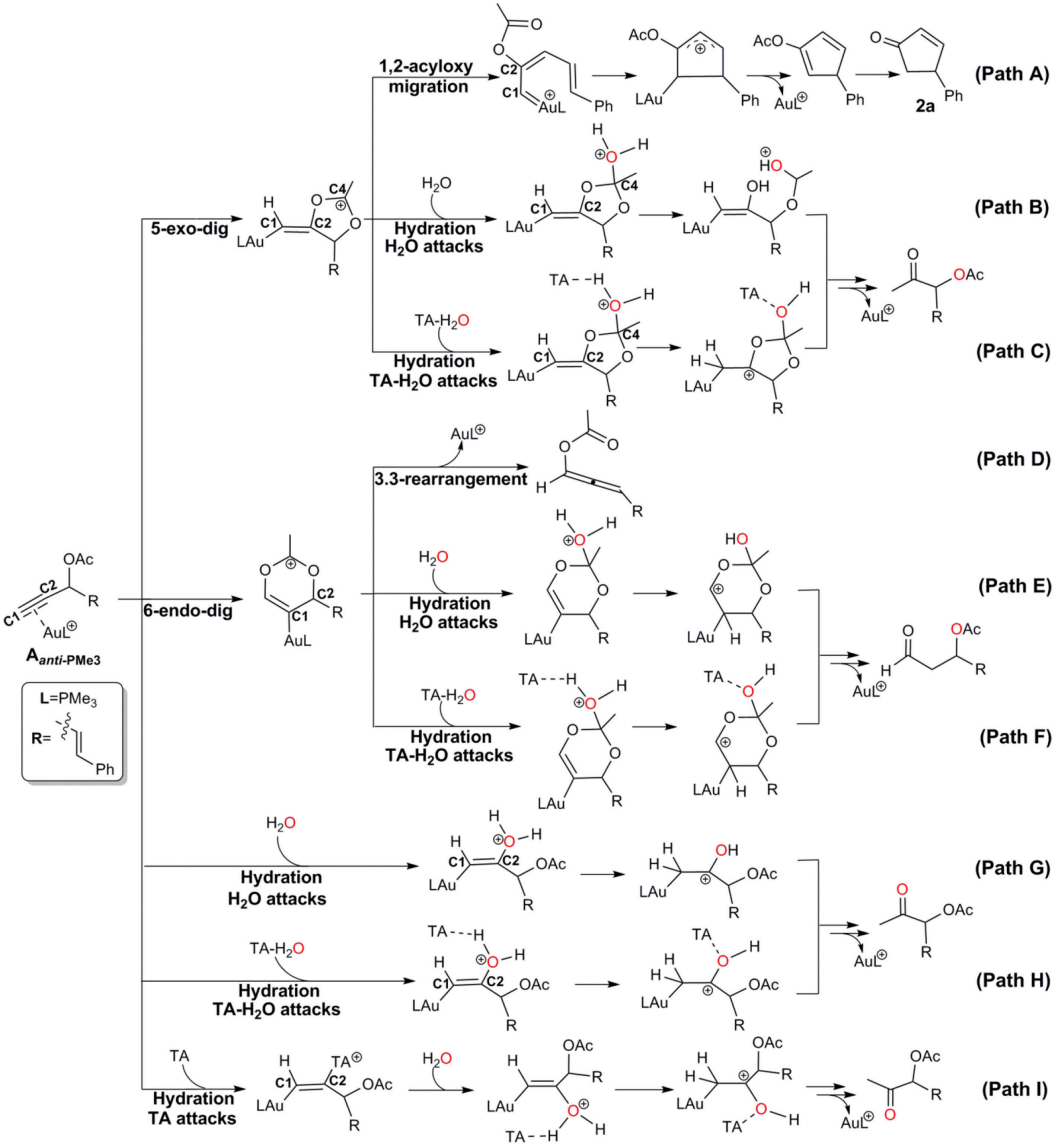
Schematic possible pathways for the Au-catalyzed propargyl ester reactions.

Under wet condition, Au-catalyzed reactions of propargyl ester can undergo two types of skeleton rearrangement, i.e., 1,2-acyloxy migration and 3,3-rearrangement (Path A and D in Scheme 2), and hydration (Path B, E, and G in Scheme 2). To understand the detailed reaction mechanisms at the molecular level, we considered all possible channels for the current model, as shown in Scheme 2 and Scheme S2.

**Figure 1:**
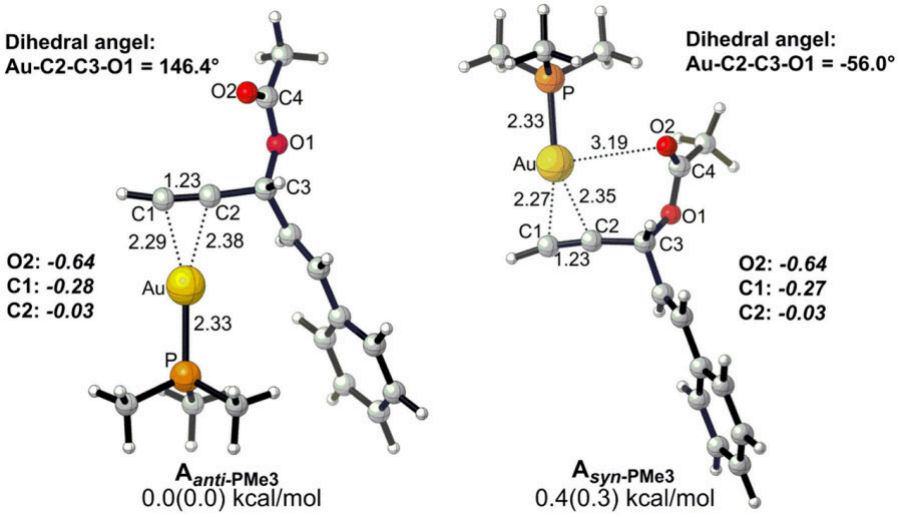
Optimized geometries for two types (*anti* and *syn*) of alkyne-coordinated [AuPMe_3_]^+^ (bond distance in Å, dihedral angel in degree. Gibbs free energy and enthalpy (in parenthesis) are given in kcal/mol). NBO charges are given for selected atoms in italic.

Before starting to investigate the detailed reaction mechanisms, we first focused on the complexes of the alkyne-coordinated [AuPMe_3_]^+^ as it is well-accepted that Au-catalyzed activation of alkynes begins with the coordination of the cationic [AuPMe_3_]^+^, to the substrate. Two types of complexes (**A**_***anti*−**PMe3_ and **A**_***syn*−**PMe3_) were found under nearly equilibrium state, slightly favoring the **A**_***anti*−**PMe3_ over **A**_***syn*−**PMe3_ (0.0 vs. 0.4 kcal/mol, Figure 1). Thus, we took **A**_***anti*−**PMe3_ as the reference, with respect to which the relative free energies were given throughout the whole work, unless otherwise noted.

### Chemoselectivity of Au-Catalyzed Propargyl Ester Reactions in the Absence of TA

**Figure 2:**
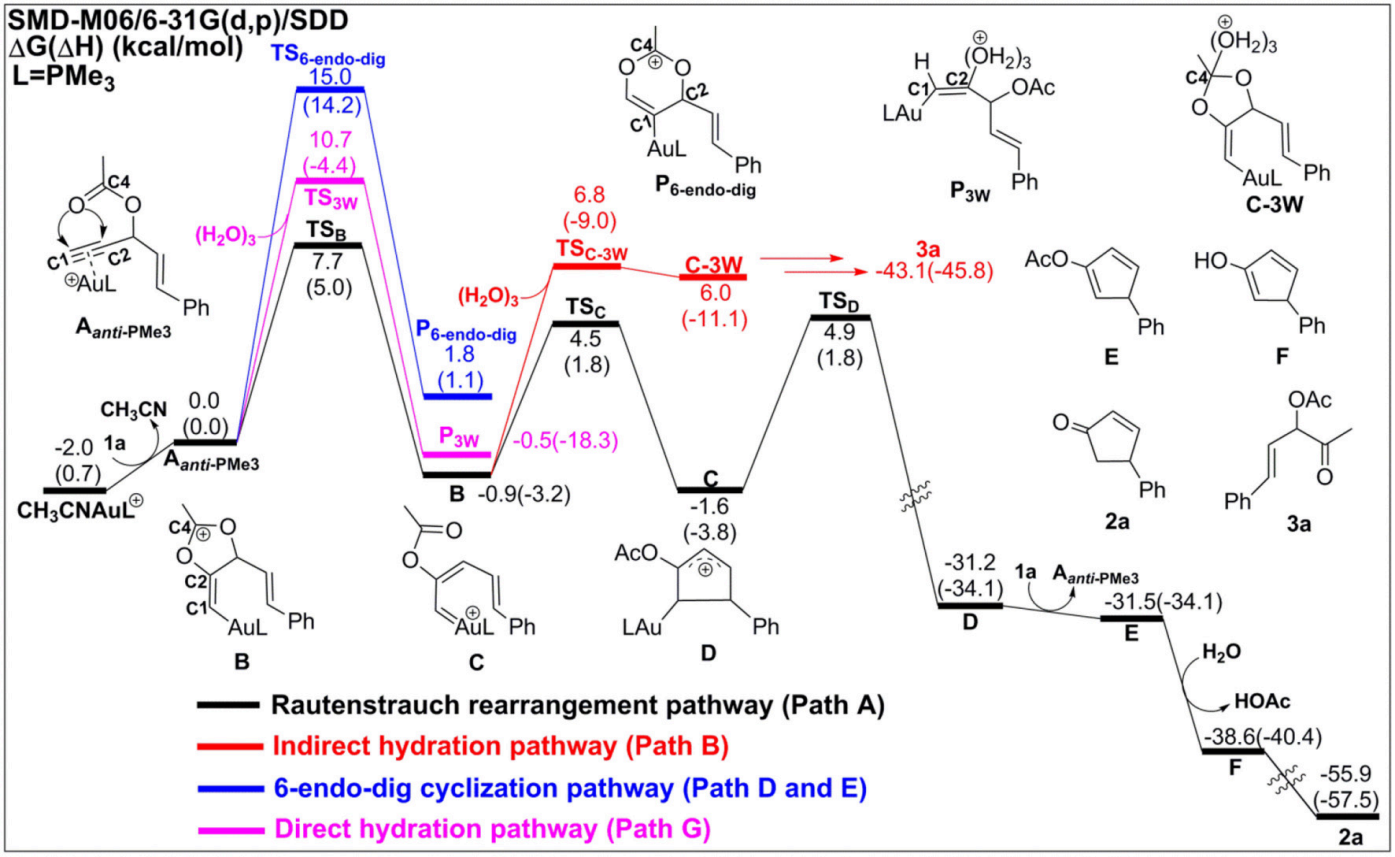
Relative Gibbs free energy and enthalpy (in parenthesis) (kcal/mol) profiles for Au-catalyzed Rautenstrauch rearrangement (black line), hydration reactions (indirect hydration in red line and direct hydration in pink line), and 6-endo-dig cyclization (blue line) of propargyl ester initiated by **A**_***anti*−**PMe3_.

In the absence of TA, acetonitrile, although it is an excellent ligand, can be easily substituted by the alkynes to form the alkyne-coordinated [AuPMe_3_]^+^ for further reactivities. The substrate exchange process is endergonic by 2.0 kcal/mol (Figure 2).

The Rautenstrauch rearrangement, namely the formation of cyclopentenone **2a**, is initialized with a 1,2-acyloxy migration as illustrated in Scheme 2 (Path A**)** and the corresponding energy profile is shown in Figure 2. A nucleophilic attack of the lone pair on the carboxyl oxygen to the **C2** site in **A**_***anti*−**PMe3_ via a 5-exo-dig cyclization, leading to the formation of a five-membered vinyl-gold intermediate **B**. This transformation only requires an activation free energy of 7.7 kcal/mol and is thermodynamically neutral process (exergonic by only 0.9 kcal/mol). **C1** and **C2** atoms in the five-membered vinyl-gold intermediate **B** change to *sp*^2^ hybridization, rendering that the formal positive charge partially distributes on **C4** atom (0.97e, Figure S3). The lone pair on the acetate group can also attack the **C1** site in **A**_***anti*−**PMe3_, leading to a 3,3-rearrangement via a 6-endo-dig cyclization (Path D in Scheme 2). However, our calculations indicate that the 6-endo-dig cyclization process is kinetically unfavorable and its apparent activation free energy is 7.3 kcal/mol higher than that for the 5-exo-dig cyclization (Figure 2). Thus, the subsequent 3,3-rearrangement and hydration reactions (Path D and E in Scheme 2) are not considered in current work. This significantly higher activation barrier (15.0 kcal/mol) via the 6-endo-dig cyclization, in comparison with that (7.7 kcal/mol) for the 5-exo-dig cyclization, is in line with its nucleophilic nature as the NBO charge analysis indicates that **C2** (−0.03e) bears less charges than **C1** (−0.28e) (Figure 1).

Note that C3–O1 bond in intermediate **B** is significantly weaker than that of normal C–O single bond (1.43 Å) and other C–O bonds in intermediate **B** (1.46 for C2–O2, 1.28 for C4–O1, and 1.28 Å for C4–O2, Figure S3), as demonstrated by its elongated bond length of 1.50 Å. Hence, the 1,2-acyloxy migration can easily take place via a cleavage of the C3–O1 bond, resulting to the isomerization into the vinyl gold-carbenoid **C** with a free energy release of 0.7 kcal/mol. The activation free energy is calculated to be 5.4 kcal/mol. Cyclization of **C** followed by the elimination of gold catalysts gives the cyclopentadiene **E** and final hydrolysis furnishes the desired cyclopentenone **2a**. The whole process proceeds smoothly with a low apparent activation free energy of 9.7 kcal/mol, and is highly exothermic with a free energy release of 53.9 kcal/mol, as shown in Figure 2.

The hydration reactions can be initiated by nucleophilic attack of water cluster at either carbocation **C4** via the favored 5-exo-dig cyclization (Path B in Scheme 2) or directly to **C2** in **A**_***anti*−**PMe3_ (Path G in Scheme 2). Due to the nature of its nucleophilic attack, the water cluster is more inclined to attack the positively charged **C4** (0.97e) (denoted as indirect hydration) instead of the negative charged **C2** (−0.03e) (denoted as direct hydration). The calculated free energy barriers are 7.7 and 10.8 kcal/mol, respectively (Figure 2). Further proton transfer and elimination of gold catalyst for both processes lead to the same ketone. In comparison to the Rautenstrauch rearrangement, the hydration reactions are clearly both kinetically and thermodynamically unfavorable, as shown in Figure 2. Thus, according to our calculations, Au catalyst selectively produces cyclopentenones in the absence of TA. In this sense, our calculations provide a theoretical support for experimental observations and insights into the chemoselectivity of Au-catalyzed propargyl ester reactions in the absence of TA.

### Chemoselectivity of Au-Catalyzed Propargyl Ester Reactions in the Presence of TA

**Scheme 3:**
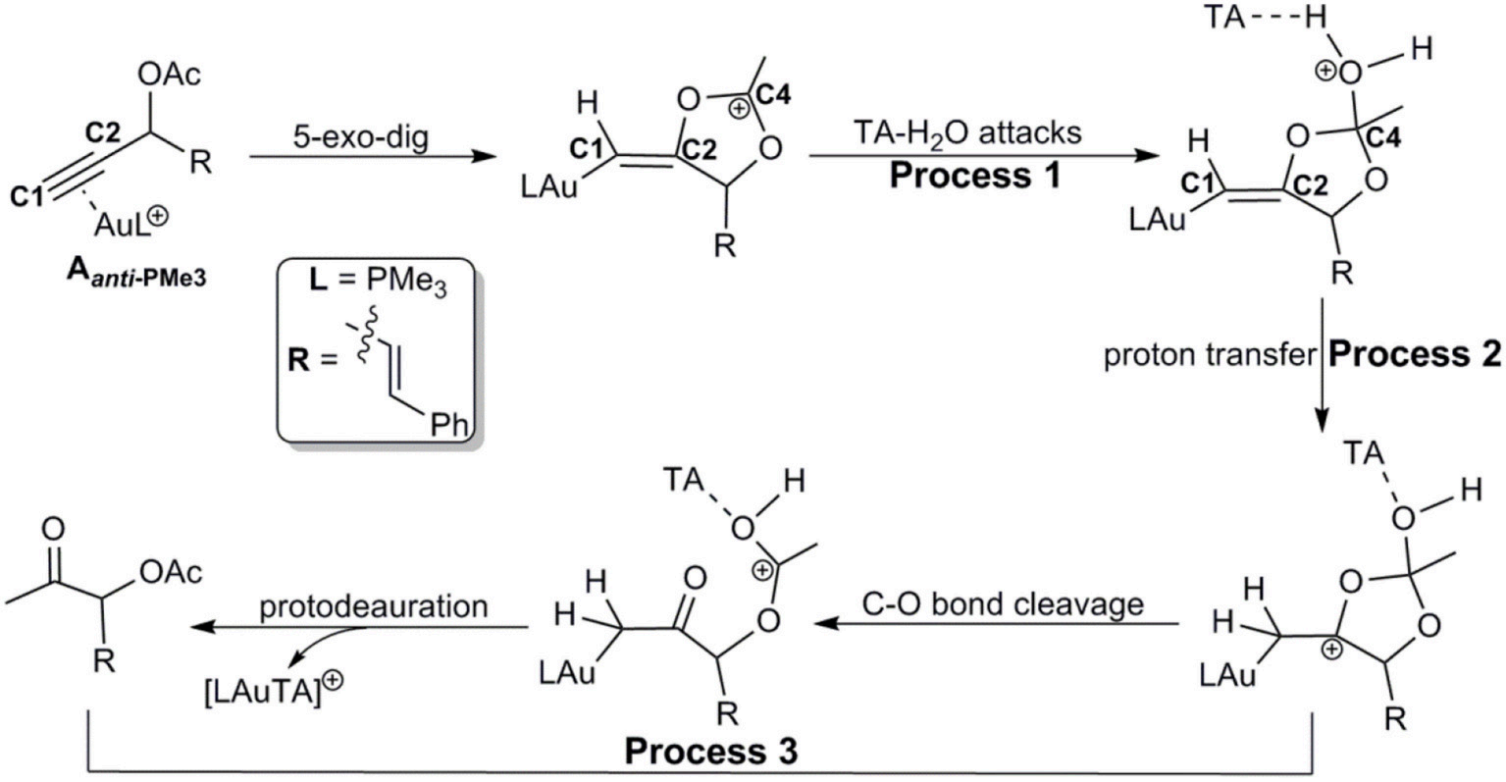
Path C for the TA-Au-catalyzed hydration of propargyl ester.

Experiments have demonstrated that TA provides unique chemoselectivity in addition to improved thermal and substrate stability (Chen et al., 2010; Wang et al., 2010, 2011a,b,c, 2012c). According to our calculations, TA can indeed stabilize the Au catalysts by coordinating with the cationic Au in [AuPMe_3_]^+^ with a stabilization free energy of 19.0 kcal/mol (Scheme S1). However, the longer Au–N bond (2.13 Å vs. 2.10 Å, Figure S4) in comparison to that in the anionic TA coordinated Au complex implies that the neutral TA can dissociate and release the coordination site for substrate activation. Under the experimental conditions, the substrate exchange process is calculated to be endergonic by 6.3 kcal/mol, leading to the alkyne-coordinated [AuPMe_3_]^+^ (Scheme S1). Subsequent Rautenstrauch rearrangement (Path A in Scheme 2) and 3,3-rearrangement (Path D in Scheme 2) are the same as that catalyzed by the [AuPMe_3_]^+^ in the absence of TA. Herein, we will focus on the hydration pathways (Path C, F, H and I in Scheme 2) of TA-Au-catalyzed propargyl ester reactions. Path F is excluded due to the high activation free energy for the 6-endo-dig cyclization as mentioned above, and the hydration pathway initialized by a direct attack of TA to **C2** (Path I) is also ruled out because of its high activation free energy of 12.3 kcal/mol.

As an electron-rich moiety, TA can be used not only as a ligand to the cationic Au but also as a good hydrogen bond acceptor. It can readily form a hydrogen-bond complex TA-(H_2_O)_3_ with water cluster although this process is endergonic by 3.2 kcal/mol. A significantly increased dipole in TA-(H_2_O)_3_ cluster [7.0 vs. 1.7 Debye in (H_2_O)_3_, Figure S4] may offer a facile route for the nucleophilic attack with the assistant of TA.

Similar as those in the absence of TA, the hydration reactions can be initiated by the nucleophilic attack of TA-(H_2_O)_3_ cluster at either the carbocation **C4** via the favored 5-exo-dig cyclization (Path C in Scheme 2) or directly to **C2** in **A**_***anti*−**PMe3_ (Path H in Scheme 2). Path C is found to be the most favorable pathway for the TA-Au-catalyzed hydration reactions. Herein, we only focus on the process of Path C in detail. To clarify the whole mechanism and the role of TA, we divided the hydration into three processes: TA-assisted nucleophilic addition of water, proton transfer, and the formation of α-acetoxy ketone, as shown in Scheme 3.

### Process 1: TA-Assisted Nucleophilic Addition of Water

**Figure 3:**
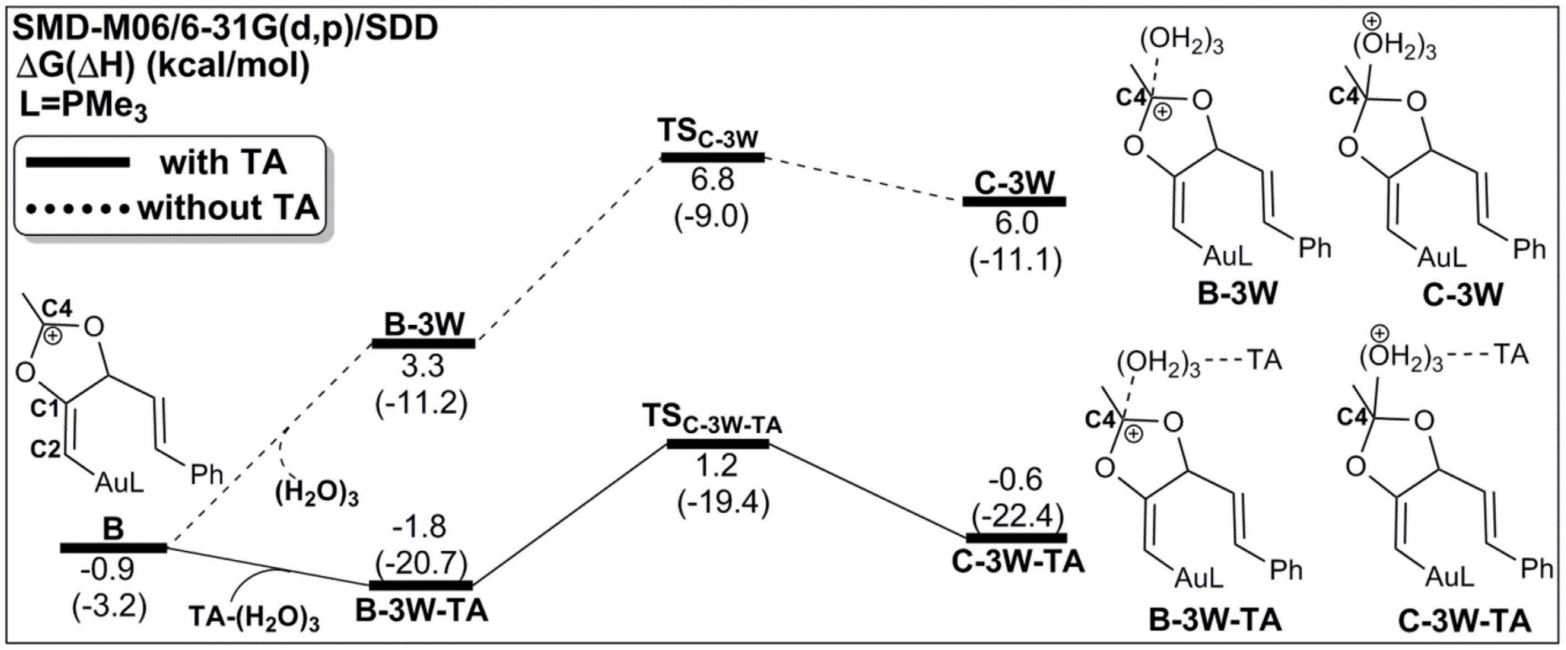
Relative Gibbs free energy and enthalpy (in parenthesis) (kcal/mol) profiles for the nucleophilic addition of water in the absence/presence of TA (dashed/solid line).

As discussed above, the water cluster is more inclined to attack the positively charged **C4** (0.97e) instead of the negative charged **C2** (−0.03e) due to the nature of nucleophilic attack. For the TA-(H_2_O)_3_ cluster, the same will occur. A complex **B-3W-TA** can be located on the potential energy surface with a stabilization energy of 0.9 kcal/mol, as shown in Figure 3. It is interesting to note that the TA-(H_2_O)_3_ cluster itself is unstable with respect to the separated TA and (H_2_O)_3_ by 3.2 kcal/mol. However, the complex **B-3W-TA** is more stable than **B-3W** in the absence of TA by 5.1 kcal/mol. This is mainly due to the enhanced electrostatic interaction caused by TA, as indicated by the increased charge transfer (0.13e) (Figure S3) between TA-(H_2_O)_3_ and the five-membered vinyl-gold intermediate **B** moiety. On the other hand, the shortened distance between **Ow1** and **C4** (2.37 in **B-3W-TA** vs. 2.50 Å in **B-3W**) also prove this (Figure S3). As a consequence, TA-assisted water addition requires a small free energy barrier of 3.0 kcal/mol, which is 4.7 kcal/mol lower than that in the absence of TA, leading to the intermediate **C-3W-TA**. In all, with the assistance of TA, it is more efficient for the water addition in this nucleophilic attack process.

### Process 2: Proton Transfer to the Terminal Carbon

**Figure 4:**
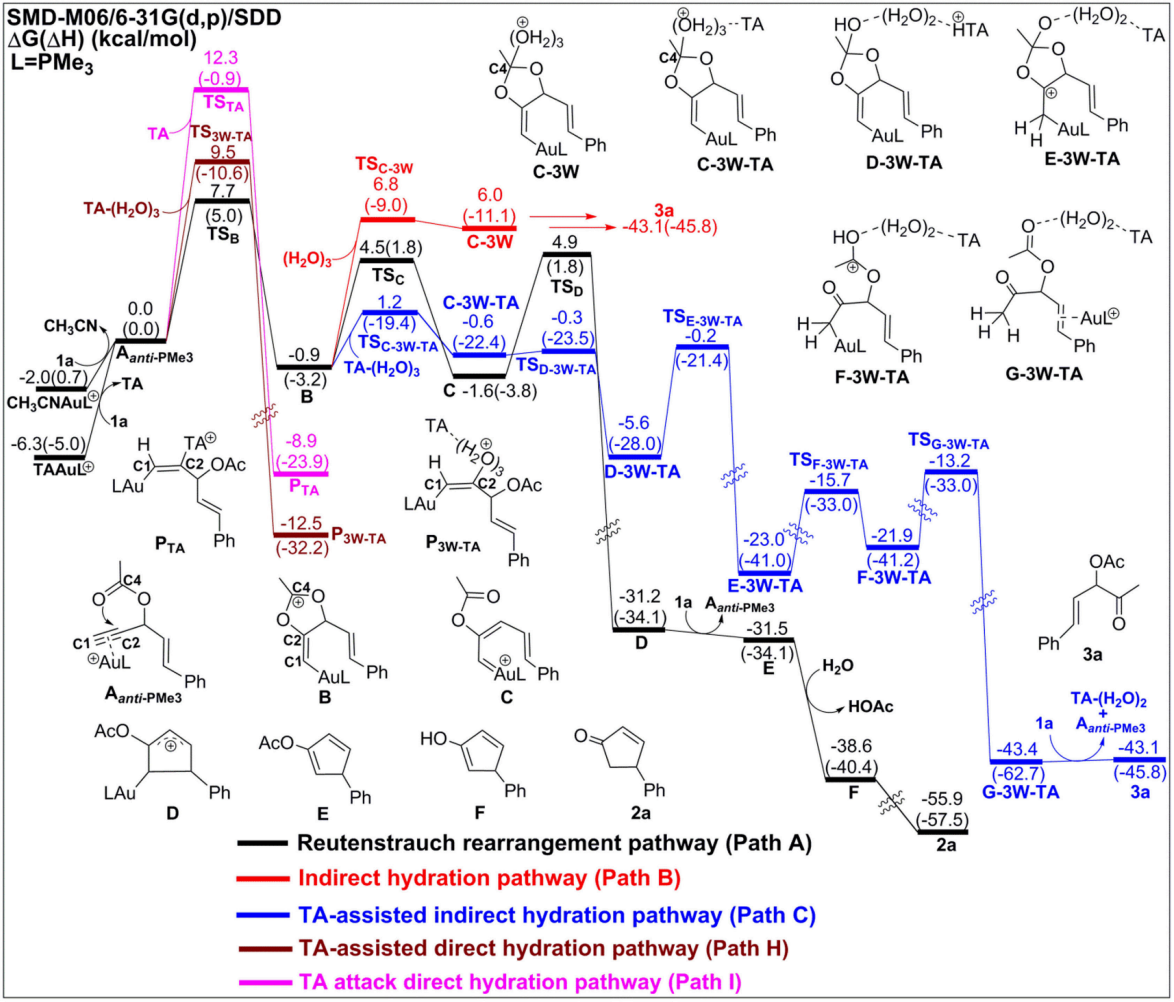
Relative Gibbs free energy and enthalpy (in parenthesis) (kcal/mol) profiles for Au-catalyzed Rautenstrauch rearrangement (black line), indirect hydrations (no-TA-assisted case in red line and TA-assisted case in blue line), TA-assisted direct hydration (purple line), and TA attack direct hydration (pink line) of propargyl ester initiated by **A**_***anti*−**PMe3_.

After the formation of intermediate **C-3W-TA**, it would be likely to undergo the protodeauration via proton-transfer, initialized by proton transfer to the terminal carbon (**C1**). A two-step pathway is located for this process as shown in Figure 4. A nearly barrierless (0.3 kcal/mol) double proton-transfer firstly takes place starting from **C-3W-TA**. That is, the proton **Hw2** from the nucleophilic attacking **Ow1** transfers to **Ow2** and simultaneously the proton **Hw3** on **Ow2** transfers to **N1** atom of TA, resulting to the intermediate **D-3W-TA** (Figure S5). This process is exergonic by 5.0 kcal/mol, which is in line with the fact that TA is a good hydrogen bond acceptor. The second step is the proton transfer from **H**_**N**_ on the TA to the terminal carbon **C1**. Note that this proton transfer step needs to overcome a barrier of 5.4 kcal/mol to form the intermediate **E-3W-TA** with a free energy release of 17.4 kcal/mol. Based on our computational results, we can draw conclusions that TA can stabilize the proton and act as a “relay” to accept and donate a proton. Similar mechanism has been generally found and accepted in protic-solvent-catalyzed organic and biochemical reactions (Fakhraian and Panbeh Riseh, 2005; Kim et al., 2006).

### Process 3: Formation of α-Acetoxy Ketone

Similar to the 1,2-acyloxy migration, we found that the C4–O2 in intermediate **E-3W-TA** is significantly weaker than that of normal C–O single bond (1.43 Å) and other C–O bonds in **E-3W-TA** (1.38 for C4–O1, 1.43 for C3–O1, and 1.31 Å for C2–O2, Figure S5), as demonstrated by its elongated bond length of 1.49 Å. Hence, cleavage of the C4–O2 bond can easily take place, with a free energy barrier of 7.3 kcal/mol, leading to an isomerization into the intermediate **F-3W-TA**. The subsequent protodeauration followed by the release of TA-(H_2_O)_2_ cluster gives the desired α-acetoxy ketone **2a**. This process requires an activation energy of 8.7 kcal/mol, accompanying by a free energy release of 21.5 kcal/mol, as displayed in Figure 4.

It is worth to note that the above mentioned reactions may also have the similar possibility to occur in **A**_***syn*−**PMe3_ as these two types of complexes (**A**_***anti*−**PMe3_ and **A**_***syn*−**PMe3_) were found under nearly equilibrium state. From our computational results (Figure S6 for all pathways initiated by **A**_***syn*−**PMe3_ in the absence/presence of TA), the *anti*-types of various paths are found to be more favorable.

According to our calculations, we can draw a conclusion that the chemoselectivity of entitled TA-Au catalyzed propargyl ester reactions would rely on the first two steps in each pathway. As shown in Figures 2, 4, the lowest free energy barrier for the first step is associated with the *anti*-type of 5-exo-dig cyclization in Path A, B, and C. That is, the intramolecular 5-exo-dig cyclization benefit from the nature of nucleophilic attack, is more favorable for the Rautenstrauch rearrangement (Path A) and hydration reactions (Path B and C). Subsequently, the existence of TA further promotes the nucleophilic attack and makes Path C the most favorable pathway in comparison with the Rautenstrauch rearrangement (Path A) and water-assisted hydration reaction (Path B). Therefore, the desired α-acetoxy ketone was obtained in the experiment with the assistance of TA. In summary, TA not only stabilizes the Au catalysts, but also alters the chemoselectivity of Au catalyzed propargyl ester reactions, and simultaneously acts as a “relay” to promote the proton transfer.

## Conclusions

The whole Au-catalyzed propargyl ester reactions have been investigated by a comprehensive DFT study. We considered all possible mechanisms, such as 3,3-rearrangement, 1,2-acyloxy migration and various hydration reactions, in the absence/presence of TA. Our computational results not only account for the experimental observations, but also clarify the role of TA. Our findings are summarized as follows.

1. Due to the nature of nucleophilic attack, 5-exo-dig cyclization is the most favorable to start the reactions in comparison to the 6-endo-dig and direct hydration.
2. The Rautenstrauch rearrangement (Path A) is found to be both kinetically and thermodynamically favorable in the absence of TA. Whereas, TA-assisted hydration is the most favorable pathway (Path C) in the presence of TA. These fit well with the experimental observed chemoselectivity of Au-catalyzed propargyl ester reactions.
3. TA does not act as a special “X-factor.” It has triple effects on this reaction. First of all, TA can stabilize and prevent rapid decomposition of the Au catalysts. Secondly, the existence of TA promotes the nucleophilic attack and alters the chemoselectivity of this reaction. Moreover, TA acts as a “relay” to promote the proton transfer.

Our calculation results not only shed light on the role of TA, but also highlight the possible way for the experimental design of more efficient catalysts with desired chemoselectivity.

## Data Availability

The raw data supporting the conclusions of this manuscript will be made available by the authors, without undue reservation, to any qualified researcher.

## Author Contributions

This work was completed by cooperation of all authors. AW and XL were responsible for the study of concept and design of the project. QS, PH, and DW searched the intermediates, transition states, analyzed the data, and drew energy profiles. QS, PH, DW, AW, KT, and XL drafted, revised, and checked the manuscript.

### Conflict of Interest Statement

The authors declare that the research was conducted in the absence of any commercial or financial relationships that could be construed as a potential conflict of interest.
