# Supplementary material for: Chemoselectivity in Gold(I)-Catalyzed Propargyl Ester Reactions: Insights From DFT Calculations
Source: Front Chem. 2019 Sep 6;7:609. doi: 10.3389/fchem.2019.00609 (PMC6743037; doi:10.3389/fchem.2019.00609)
Supplement: Supplementary file 1 [file Table_1.DOCX]

Supplementary Material

**Chemoselectivity in Gold(I)-Catalyzed Propargyl Ester Reactions: Insights from DFT Calculations**

Qing Sun, Pan Hong, Dongdong Wei, Anan Wu*, Kai Tan, Xin Lu*

State Key Laboratory of Physical Chemistry of Solid Surface & Fujian Provincial Key Laboratory for Theoretical and Computational Chemistry, Department of Chemistry, College of Chemistry and Chemical Engineering, Xiamen University, Xiamen, China
E-mail: ananwu@xmu.edu.cn; xinlu@xmu.edu.cn





**Scheme S1.** The formation process of [TAAuPPh_3_]^+^/[TAAuPMe_3_]^+^, the substrate exchange process of [TAAuPPh_3_]^+^/[TAAuPMe_3_]^+^ with the substrate **1a**, as well as the energies of **TS_C-1W-PPh3_**/**TS_C-1W_**. Gibbs free energy and enthalpy (in parenthesis) are given in kcal/mol.

**_

_**

**Scheme S2.** Schematic possible pathways for the Au-catalyzed propargyl ester reactions initiated from **A*_syn_*_-PMe3_**.





**Figure S1.** Relative Gibbs free energy and enthalpy (in parenthesis) (kcal/mol) profiles for the TA-free nucleophilic addition of water clusters ((H_2_O)_n_, n=1-4) to the 5-exo-dig cyclization intermediate **B**.





**Figure S2.** Relative Gibbs free energy and enthalpy (in parenthesis) (kcal/mol) profiles for the TA-involving nucleophilic addition of water clusters ((H_2_O)_n_, n=1-4) to the 5-exo-dig cyclization intermediate **B**.





**Figure S3.** Optimized geometries (bond distance in Å) for the nucleophilic addition of water in the absence/presence of TA. NBO charges are given for selected atoms in italic.

**_

_**

**Figure S4.** Optimized geometries (bond distance in Å) for [TAAuPMe_3_]^+^, TAAuPMe_3_, (H_2_O)_3_ and TA-(H_2_O)_3_, together with the dipole moments (in Debye) of (H_2_O)_3_ and TA-(H_2_O)_3_.





**Figure S5.** Optimized geometries (bond distance in Å) for the Process 2 and Process 3 of Path C.





**Figure S6.** Relative Gibbs free energy and enthalpy (in parenthesis) (kcal/mol) profiles for all the first steps of Au-catalyzed propargyl ester reactions initiated from **A*_syn_*_-PMe3._**

**Cartesian Coordinates and Thermochemical Data (Energies in Hartree)**

**A*_anti_*_-PMe3_**

Number of imaginary frequencies: 0

C 3.76966500 4.48204300 -2.89893900

O 4.26846300 5.27865100 -1.81754700

C 3.44678800 5.39626700 -0.74343000

O 2.34637700 4.89976700 -0.71036900

C 4.09443900 6.20254700 0.32928700

H 4.30027600 7.21256000 -0.04073400

H 5.05393900 5.75605000 0.60879000

H 3.43872700 6.25505700 1.19948500

C 2.70518000 5.22358400 -3.60504900

C 1.81210400 5.88428100 -4.12683300

H 0.98499200 6.51896200 -4.39060000

Au 2.66517200 4.76617600 -5.93971100

H 3.33578100 3.55125200 -2.50550500

C 4.89845700 4.18680100 -3.83371000

H 5.44515300 5.05549100 -4.20401800

C 5.15440700 2.94038900 -4.25212900

C 6.12381000 2.54900200 -5.27380400

C 6.76664600 3.48190800 -6.10242200

C 6.38677500 1.18638400 -5.46685000

C 7.65048900 3.05887300 -7.08630700

H 6.56294500 4.54605500 -5.98636700

C 7.27333100 0.76408300 -6.45146200

H 5.88552600 0.45606100 -4.83273300

C 7.90705200 1.69925800 -7.26464300

H 8.13807500 3.79353000 -7.72389100

H 7.46699400 -0.29772000 -6.58604100

H 8.59614400 1.37169900 -8.03971700

H 4.57343300 2.12274400 -3.81670700

P 3.08849600 3.94269600 -8.07347600

C 3.57097000 2.19175700 -8.08692100

C 1.64790700 4.05010500 -9.17449800

C 4.42368500 4.80769900 -8.94975800

H 1.90815700 3.63855200 -10.15727500

H 0.81516800 3.48154800 -8.74854600

H 1.34611600 5.09656900 -9.28497300

H 4.49361800 2.05917800 -7.50985700

H 2.77769000 1.58698700 -7.63594400

H 3.73696200 1.86708800 -9.12137700

H 4.53634400 4.38113800 -9.95395000

H 4.18660600 5.87333100 -9.02917200

H 5.36247100 4.68870600 -8.39751500

Electronic Energy (0K) = -1249.1996686

Electronic Energy (0K) + ZPE = -1248.872582

Enthalpy (298K) = -1248.846706

Free Energy (298K) = -1248.931048

**A*_syn_*_-PMe3_**

Number of imaginary frequencies: 0

C 1.58850300 -0.53226900 -0.22572300

O 1.64207900 -0.14281300 1.15918700

C 0.59752600 -0.49608600 1.94338500

O -0.35771300 -1.12182300 1.53793900

C 0.78443300 0.01947300 3.32873800

H 0.08116400 -0.46583800 4.00777700

H 0.59752100 1.10070500 3.32741100

H 1.81225600 -0.13434900 3.66851000

C 0.99548500 0.56182000 -1.01560800

C 0.63693800 1.45794200 -1.77647000

H 0.52026800 2.21379400 -2.53319300

Au -1.13466600 1.31679600 -0.35970600

H 0.96364900 -1.42960900 -0.32742200

C 2.98169100 -0.79326100 -0.69663500

H 3.67919200 0.03924200 -0.59778100

C 3.33529200 -1.97644200 -1.21288300

C 4.65728100 -2.35784500 -1.70665200

C 5.73954300 -1.46628900 -1.75304200

C 4.85304500 -3.67293300 -2.14802000

C 6.97652200 -1.88377000 -2.22286800

H 5.61256500 -0.43762800 -1.42089500

C 6.09272800 -4.09093700 -2.61836400

H 4.01700500 -4.37066700 -2.11608100

C 7.15861600 -3.19709700 -2.65642900

H 7.80652700 -1.18121200 -2.25327400

H 6.22620600 -5.11639000 -2.95569200

H 8.13000000 -3.51993400 -3.02431000

H 2.57283800 -2.75749800 -1.27417700

P -3.09750200 1.40562600 0.88659700

C -2.78490100 1.15489500 2.65880200

C -4.27127600 0.09795700 0.42806500

C -4.02671800 2.96052400 0.76868100

H -5.15850800 0.15547700 1.07021700

H -4.56767600 0.21650500 -0.61896600

H -3.79113500 -0.87855900 0.55431600

H -2.15071200 1.96195400 3.03985300

H -3.73721600 1.15151000 3.20312900

H -2.27535900 0.19508800 2.80109100

H -3.40057800 3.79077000 1.11033500

H -4.31884400 3.13800900 -0.27112900

H -4.92460200 2.89680700 1.39513900

Electronic Energy (0K) = -1249.1993045

Electronic Energy (0K) + ZPE = -1248.871801

Enthalpy (298K) = -1248.846272

Free Energy (298K) = -1248.930370

**A*_anti_*_-PPh3_**

Number of imaginary frequencies: 0

C 3.91508800 4.39762400 -2.90677700

O 4.25512700 5.47322700 -2.02025500

C 3.53581500 5.53745600 -0.87100900

O 2.63213300 4.77373900 -0.62846300

C 4.02507400 6.64501800 -0.00262600

H 4.00687300 7.59099300 -0.55291700

H 5.06420100 6.45604100 0.28716500

H 3.40126600 6.71875000 0.88926300

C 2.60435500 4.67434100 -3.52586700

C 1.48526800 4.94867300 -3.95131000

H 0.45819100 5.23015000 -4.10441500

Au 2.47742700 4.24467600 -5.86318600

H 3.82887000 3.46244200 -2.33476900

C 4.98863000 4.28678300 -3.94065400

H 5.18728400 5.19566200 -4.51193200

C 5.61989300 3.12825700 -4.17259600

C 6.61034000 2.86076100 -5.21325700

C 7.03716200 3.83379600 -6.12992700

C 7.11136100 1.55754400 -5.33658300

C 7.92585300 3.50378000 -7.14395800

H 6.66883600 4.85633000 -6.05801800

C 8.00186900 1.22830900 -6.35280500

H 6.78257900 0.79620300 -4.62977000

C 8.40671600 2.19970600 -7.26357600

H 8.24113200 4.26920200 -7.85086700

H 8.37624800 0.21027400 -6.43564000

H 9.09781600 1.94493300 -8.06378700

H 5.35600900 2.27004900 -3.54860900

P 2.91227300 3.67059500 -8.08488400

C 3.21496300 1.89220100 -8.32225000

C 4.25997300 1.30691100 -7.59734100

C 2.44944800 1.11610700 -9.19366600

C 4.53760900 -0.04508900 -7.74867400

H 4.86504200 1.91401900 -6.92105700

C 2.72925200 -0.24152700 -9.33502100

H 1.63796300 1.56458600 -9.76441200

C 3.76942800 -0.82096600 -8.61598000

H 5.35413600 -0.49415100 -7.18678100

H 2.13163900 -0.84530200 -10.01388100

H 3.98441900 -1.88089400 -8.73069300

C 1.50937100 4.10488000 -9.15368000

C 1.68998900 4.41791700 -10.50386100

C 0.22063100 4.05968800 -8.61205000

C 0.58402900 4.68624800 -11.30367800

H 2.69052800 4.45271900 -10.93255100

C -0.88120100 4.32200000 -9.41885200

H 0.08517500 3.82047600 -7.55627200

C -0.69846400 4.63722100 -10.76287500

H 0.72537100 4.93407800 -12.35297300

H -1.88244100 4.28682600 -8.99623200

H -1.55997600 4.84958600 -11.39178100

C 4.38507500 4.51659100 -8.73244800

C 5.14722900 3.95462200 -9.76199800

C 4.73870300 5.76131700 -8.20159200

C 6.24801500 4.64209400 -10.26182600

H 4.88154400 2.98175500 -10.17390400

C 5.84018700 6.44504300 -8.70666200

H 4.15156700 6.19039000 -7.38829300

C 6.59320400 5.88548500 -9.73623500

H 6.83921200 4.20412100 -11.06246600

H 6.11412300 7.41182600 -8.29096000

H 7.45683900 6.41836100 -10.12760300

Electronic Energy (0K) = -1823.967567

Electronic Energy (0K) + ZPE = -1823.479935

Enthalpy (298K) = -1823.444815

Free Energy (298K) = -1823.551392

**A*_syn_*_-PPh3_**

Number of imaginary frequencies: 0

C 4.27372800 4.16067700 -3.10406500

O 3.91132200 2.87876800 -3.64160700

C 4.44899600 2.54324500 -4.83982200

O 5.21157600 3.26156700 -5.44658600

C 3.94472700 1.21715200 -5.29214500

H 2.92178300 1.34824900 -5.67279600

H 3.90080100 0.50587700 -4.46301100

H 4.57553500 0.83386500 -6.09623000

C 3.27267500 5.16616300 -3.50652300

C 2.44863100 6.06661300 -3.65384100

H 1.74892400 6.87922200 -3.56330600

Au 2.58225800 5.24835700 -5.75735000

H 5.25535800 4.45628600 -3.49762500

C 4.29487000 4.07020000 -1.61308700

H 3.37963600 3.70089400 -1.14907000

C 5.36040300 4.46147900 -0.90387900

C 5.49635400 4.45799700 0.55148300

C 4.48677300 3.99950900 1.41111600

C 6.68773800 4.93653500 1.11210900

C 4.66880300 4.02225500 2.78643600

H 3.55234800 3.62103000 1.00107900

C 6.86890200 4.96086200 2.49024000

H 7.47616600 5.29428600 0.45095300

C 5.85932600 4.50344000 3.33162700

H 3.87736500 3.66293800 3.44059500

H 7.80002900 5.33763900 2.90754400

H 5.99724900 4.52038500 4.41043100

H 6.23224400 4.83043200 -1.45027500

P 2.37277300 4.70047800 -8.01994300

C 2.18004200 2.90417200 -8.23173000

C 1.12268800 2.27244500 -7.56426800

C 3.06537400 2.15241600 -9.00552600

C 0.94710200 0.89984300 -7.68569700

H 0.43530500 2.85860900 -6.95309900

C 2.89023100 0.77409100 -9.11276600

H 3.89090900 2.63575000 -9.52510000

C 1.83444400 0.14960700 -8.45736300

H 0.12187000 0.41260100 -7.17190700

H 3.58258000 0.18992800 -9.71431800

H 1.70045000 -0.92602900 -8.54540900

C 3.82129500 5.20174100 -8.99210800

C 5.05587500 5.32966000 -8.34885100

C 3.72301900 5.41457700 -10.37085100

C 6.18778200 5.66695900 -9.08388100

H 5.12594100 5.15994500 -7.27410600

C 4.85774800 5.75532700 -11.09836300

H 2.76290200 5.31345200 -10.87557600

C 6.08752700 5.88159600 -10.45577500

H 7.14753600 5.76822600 -8.58281300

H 4.78130400 5.92446900 -12.16978500

H 6.97207600 6.15150700 -11.02820000

C 0.92589200 5.48617200 -8.78664000

C 0.60668100 6.79143000 -8.39822800

C 0.17091700 4.84407200 -9.77201600

C -0.46120100 7.45129400 -8.99493000

H 1.19774600 7.28781200 -7.62762300

C -0.89999300 5.50886900 -10.36161000

H 0.41831600 3.82888400 -10.07950300

C -1.21551300 6.80841100 -9.97393500

H -0.70931500 8.46540300 -8.69102500

H -1.48896000 5.00892600 -11.12684600

H -2.05532000 7.32236000 -10.43579300

Electronic Energy (0K) = -1823. 9650745

Electronic Energy (0K) + ZPE = -1823.477393

Enthalpy (298K) = -1823.442355

Free Energy (298K) = -1823.549320

**In the absence of TA**

**A*_anti_*_-PMe3_ as the precursor**

**CH_3_CN**

Number of imaginary frequencies: 0

N -4.55809300 1.91370800 2.61397900

C -4.67211200 2.54735500 3.58036700

C -4.81355300 3.33750100 4.78399800

H -3.98366400 4.04566800 4.86683600

H -5.75397100 3.89574800 4.75850300

H -4.81194300 2.68598800 5.66288800

Electronic Energy (0K) = -132.6659112

Electronic Energy (0K) + ZPE = -132.620764

Enthalpy (298K) = -132.616271

Free Energy (298K) = -132.644728

**[CH_3_CNAuPMe_3_]^+^**

Number of imaginary frequencies: 0

N -3.13714500 -0.08096700 2.64930700

Au -2.99449700 -2.16920800 2.45518200

P -2.81832800 -4.45197000 2.22824000

C -1.70891900 -4.95221100 0.88038100

C -4.39929100 -5.27055800 1.87117600

C -2.17423900 -5.28306400 3.70864100

H -0.70465400 -4.55726500 1.06396000

H -1.66747200 -6.04683500 0.82674200

H -2.08057700 -4.55375900 -0.06901400

H -4.23685100 -6.35022100 1.76773600

H -5.10405200 -5.08364900 2.68757300

H -4.81739600 -4.87299500 0.94095100

H -1.17706900 -4.89723200 3.94340300

H -2.83919600 -5.09232600 4.55702700

H -2.11542000 -6.36266600 3.52504600

C -3.22158100 1.06639000 2.76358000

C -3.32612500 2.49398200 2.90686600

H -2.46285600 2.87037700 3.46368500

H -3.35167100 2.96063900 1.91775000

H -4.24375000 2.74148200 3.44893800

Electronic Energy (0K) = -729.3276986

Electronic Energy (0K) + ZPE = -729.167140

Enthalpy (298K) = -729.152713

Free Energy (298K) = -729.209465

**1a**

Number of imaginary frequencies: 0

C 3.42020000 3.93868800 -2.73603800

O 3.95757200 4.21947300 -1.42202000

C 3.11466700 4.03515600 -0.38475000

O 1.97356900 3.65678700 -0.51529700

C 3.79076200 4.36208400 0.90618700

H 4.07385400 5.42010300 0.91703600

H 4.71074300 3.77811300 1.01020500

H 3.11944600 4.15333200 1.74061000

C 2.57497100 5.05006600 -3.17442200

C 1.89585200 5.96759800 -3.57061300

H 1.29206800 6.78109700 -3.91677100

H 2.81724200 3.02163800 -2.68089400

C 4.58321500 3.74613100 -3.65479100

H 5.25245000 4.60187400 -3.75304500

C 4.77679600 2.60537400 -4.32537100

C 5.85820400 2.31027600 -5.26637600

C 6.78493600 3.27405200 -5.69117800

C 5.97507800 1.00677000 -5.76661700

C 7.79824300 2.93683500 -6.57783000

H 6.70855400 4.29877600 -5.33214000

C 6.99045100 0.66890000 -6.65448500

H 5.25677500 0.25268600 -5.44656600

C 7.90690500 1.63337800 -7.06227600

H 8.50770900 3.69680000 -6.89845100

H 7.06472800 -0.34966300 -7.02934700

H 8.70181800 1.37403600 -7.75804500

H 4.06601300 1.79037200 -4.16426400

Electronic Energy (0K) = -652.537361

Electronic Energy (0K) + ZPE = -652.324774

Enthalpy (298K) = -652.309121

Free Energy (298K) = -652.369532

**TS_B_**

Number of imaginary frequencies: 1

C -0.22794200 -0.35620400 2.70369500

O -0.09853200 -0.14108700 4.13273700

C -1.24997900 0.21997300 4.67947500

O -2.21982900 0.44249800 3.95420000

C -1.23993400 0.31064800 6.15484100

H -2.14780200 0.79868700 6.51027800

H -0.35175700 0.85515400 6.48831000

H -1.18112000 -0.70232400 6.56869700

C -1.29831800 0.52948200 2.19699100

C -1.85078800 1.25009800 1.31299200

H -2.80112400 1.77216500 1.32270100

Au -0.61181600 1.38139700 -0.42053700

H -0.51333000 -1.40910700 2.56224500

C 1.06653000 -0.05628800 2.03268500

H 1.45014100 0.95351400 2.18365400

C 1.66525700 -0.95572100 1.24169400

C 2.87239400 -0.74882600 0.44502700

C 3.47253000 0.51017300 0.28355000

C 3.43947200 -1.85120200 -0.20815600

C 4.61154900 0.65166500 -0.49823600

H 3.04347400 1.38735600 0.76640200

C 4.58011700 -1.70812500 -0.99034600

H 2.97463300 -2.82960800 -0.09302000

C 5.17004400 -0.45590300 -1.13687400

H 5.06606900 1.63342100 -0.61454000

H 5.00777200 -2.57579700 -1.48754100

H 6.06068400 -0.33974100 -1.75018600

H 1.21341100 -1.94763400 1.15872900

P 0.61389900 1.64204700 -2.39744000

C 1.53403800 0.16750200 -2.93704200

C 1.86568300 2.96111200 -2.33399900

C -0.43136100 2.07723400 -3.82145100

H 2.37258400 3.03546300 -3.30394600

H 1.38371800 3.91585300 -2.09972600

H 2.60252100 2.73369600 -1.55614900

H 0.84318500 -0.67228400 -3.06458600

H 2.03428000 0.37726100 -3.89055600

H 2.28452200 -0.09812400 -2.18308200

H -1.16760700 1.28511600 -3.99242200

H -0.96031400 3.01344600 -3.61477100

H 0.18983800 2.19866600 -4.71716600

Electronic Energy (0K) = -1249.1908862

Electronic Energy (0K) + ZPE = -1248.863374

Enthalpy (298K) = -1248.838813

Free Energy (298K) = -1248.918855

**B**

Number of imaginary frequencies: 0

C -0.06566100 -0.59233400 2.70090800

O 0.18268500 -0.40768300 4.17104500

C -0.80385800 0.24099200 4.67413400

O -1.69000900 0.65043900 3.84467800

C -0.91568100 0.47752300 6.11094800

H -1.53812400 1.35210000 6.30670800

H 0.07835100 0.58783200 6.55029200

H -1.39067900 -0.40356000 6.56197900

C -1.24208400 0.32094300 2.49151200

C -1.82223300 0.82737000 1.42105700

H -2.68640000 1.46712500 1.61318000

Au -1.11358800 0.47487900 -0.50349200

H -0.32746000 -1.65463500 2.60280900

C 1.13265900 -0.25405600 1.90700100

H 1.45333200 0.78839900 1.93462700

C 1.70870900 -1.16447700 1.10922400

C 2.77842400 -0.91781400 0.14642600

C 3.12686200 0.37491000 -0.27396100

C 3.45047600 -2.01263100 -0.41338800

C 4.12845200 0.56122400 -1.21790700

H 2.59697100 1.23823900 0.12629000

C 4.45604500 -1.82428300 -1.35475900

H 3.17493200 -3.01894000 -0.10013200

C 4.79632500 -0.53657800 -1.76054500

H 4.38550400 1.56844700 -1.53914800

H 4.97065900 -2.68430200 -1.77712000

H 5.57667000 -0.38657600 -2.50318800

H 1.33757000 -2.19224700 1.14973700

P -0.33359500 0.12219300 -2.70159500

C 0.68260300 -1.37234900 -2.93724700

C 0.71322900 1.46092200 -3.35993700

C -1.66150100 -0.04974400 -3.93783600

H 1.01415900 1.23091400 -4.38940800

H 0.15995000 2.40568700 -3.34455200

H 1.60791500 1.56372800 -2.73538100

H 0.12798700 -2.25562500 -2.60289000

H 0.94108700 -1.48473700 -3.99741600

H 1.60378800 -1.29017200 -2.34768200

H -2.28915700 -0.91051500 -3.68393200

H -2.28359800 0.85151000 -3.93645300

H -1.23154200 -0.19443700 -4.93646400

Electronic Energy (0K) = -1249.20626

Electronic Energy (0K) + ZPE = -1248.876213

Enthalpy (298K) = -1248.851810

Free Energy (298K) = -1248.932507

**TS_C_**

Number of imaginary frequencies: 1

C 4.91359600 3.63521200 0.28006800

O 5.49747000 3.01842200 -1.42508700

C 5.02280300 3.89316500 -2.16831200

O 4.19086100 4.77918600 -1.65775300

C 5.30176500 4.00996000 -3.61083000

H 6.17227200 3.41183300 -3.88147200

H 5.45380000 5.06037800 -3.87466100

H 4.42532200 3.65187600 -4.16391000

C 3.82641600 4.40231600 -0.32982500

C 2.65388300 4.74836800 0.19854500

H 2.03351100 5.36949300 -0.45387600

Au 2.05862600 4.25245100 2.12794000

P 1.40526700 3.69771200 4.32893700

H 5.86551300 4.15064500 0.43446800

C 4.62166200 2.56421200 1.16913500

H 3.66449500 2.05885300 1.05111300

C 5.47482400 2.27416500 2.18531000

C 5.23379700 1.35990500 3.27841600

C 4.03462600 0.63566300 3.41560500

C 6.22193300 1.23254400 4.26839900

C 3.84286400 -0.19450500 4.50873200

H 3.24843000 0.72672600 2.66775800

C 6.02438400 0.40273800 5.36333700

H 7.14735300 1.79728100 4.16570600

C 4.83507700 -0.31138600 5.48434600

H 2.91470400 -0.75284400 4.60759100

H 6.79618600 0.31250600 6.12361600

H 4.67660100 -0.96121000 6.34185600

H 6.42142100 2.81839400 2.22326600

C 2.78794200 3.56919700 5.51039700

C 0.54824900 2.09689800 4.48801100

C 0.26573500 4.88575100 5.10989200

H 3.32331200 4.52338700 5.55685100

H 2.40740300 3.31595700 6.50757100

H 3.48398600 2.78869300 5.17937800

H -0.36335400 2.10432800 3.88134400

H 1.20239600 1.29286100 4.13222900

H 0.28639900 1.91351900 5.53731300

H 0.73638500 5.87360900 5.15123500

H -0.65209500 4.95793900 4.51708600

H 0.01748600 4.55784800 6.12671900

Electronic Energy (0K) = -1249.1964812

Electronic Energy (0K) + ZPE = -1248.868011

Enthalpy (298K) = -1248.843768

Free Energy (298K) = -1248.923952

**C**

Number of imaginary frequencies: 0

C 2.22796600 0.86999300 -1.63370500

O 2.59931000 0.27329800 -4.22481600

C 2.11331900 1.35689400 -4.44486800

O 1.34767900 2.01680000 -3.53318500

C 2.23785300 2.15007200 -5.69813900

H 2.88934200 1.63224600 -6.40316800

H 2.64097900 3.14264200 -5.47269200

H 1.24785000 2.29441600 -6.14326000

C 1.08267700 1.35920100 -2.32374900

C -0.19879600 1.39128700 -1.86099600

H -0.89313500 1.80746800 -2.59927500

Au -0.92468700 0.88173700 0.00733600

P -1.82793800 0.45597200 2.15157800

H 3.18185600 1.34461400 -1.86473400

C 2.21524000 -0.10874600 -0.65420400

H 3.08679700 -0.18742600 -0.00654900

C 1.19386100 -1.04494600 -0.54306300

C 1.05487300 -2.02522200 0.49522500

C 1.77411300 -1.95477700 1.70528000

C 0.14740500 -3.08295700 0.29311600

C 1.59748200 -2.92858100 2.67281600

H 2.45985400 -1.12896100 1.88329300

C -0.01748200 -4.06059400 1.26083600

H -0.41813600 -3.12050700 -0.63691600

C 0.70848300 -3.98374400 2.44908500

H 2.15100600 -2.87370600 3.60651500

H -0.71259700 -4.87976400 1.09737900

H 0.57803200 -4.74775600 3.21193500

H 0.51161700 -1.14898800 -1.38853800

C -2.11557700 -1.29721300 2.55438300

C -3.44374400 1.24944200 2.42818700

C -0.79233300 1.05088900 3.52698300

H -1.15506100 -1.82249800 2.60788000

H -2.62304500 -1.37549100 3.52379800

H -2.73402800 -1.76485100 1.78086400

H -3.35156400 2.33119100 2.28525400

H -4.17086000 0.85989000 1.70792500

H -3.79468500 1.04454700 3.44690400

H 0.18086300 0.54826500 3.49504300

H -0.63475400 2.13022400 3.43121400

H -1.28068100 0.83705200 4.48555200

Electronic Energy (0K) = -1249.2061152

Electronic Energy (0K) + ZPE = -1248.877535

Enthalpy (298K) = -1248.852725

Free Energy (298K) = -1248.933596

**TS_D_**

Number of imaginary frequencies: 1

C 2.69833800 0.22915600 -1.54315600

O 3.07244100 0.29313600 -4.28676200

C 2.29857400 1.21302700 -4.26622200

O 1.41855700 1.41901000 -3.22804900

C 2.11247500 2.26348100 -5.30167500

H 2.80763300 2.09936400 -6.12573400

H 2.27968100 3.25163400 -4.86043100

H 1.08211000 2.24279600 -5.67127100

C 1.46633300 0.58596000 -2.13128300

C 0.26172600 0.10963400 -1.64402200

H -0.52258000 0.06304100 -2.41100900

Au -0.50472800 0.15549100 0.30786300

P -1.52325800 0.34159400 2.41356500

H 3.61289900 0.78778000 -1.71815500

C 2.64257300 -0.88343000 -0.73982400

H 3.44201500 -1.11187900 -0.03704300

C 1.52107200 -1.73485300 -0.83021800

C 1.12075700 -2.68754200 0.18876100

C 1.62108400 -2.62956700 1.50112800

C 0.21232700 -3.70140300 -0.15385100

C 1.24207200 -3.58262200 2.43354400

H 2.29996700 -1.82874000 1.79000600

C -0.17913700 -4.64260500 0.78781000

H -0.18032900 -3.73967000 -1.16916500

C 0.34049400 -4.58795900 2.08005900

H 1.63810100 -3.53799900 3.44529600

H -0.88177400 -5.42572600 0.51438500

H 0.03968800 -5.32890900 2.81707600

H 1.12893000 -1.93628800 -1.82885600

C -1.13508900 -1.02456200 3.55305700

C -3.34196500 0.36231600 2.34188200

C -1.09065500 1.84351600 3.34589400

H -0.05939300 -1.03713200 3.75938500

H -1.68392400 -0.89273500 4.49373300

H -1.42169000 -1.97766900 3.09410600

H -3.67685900 1.21519200 1.74238100

H -3.70175000 -0.56057600 1.87518800

H -3.75413300 0.44399600 3.35498000

H -0.01042600 1.86854200 3.52289600

H -1.37497200 2.72969900 2.76909500

H -1.61813100 1.84965700 4.30749200

Electronic Energy (0K) = -1249.196061

Electronic Energy (0K) + ZPE = -1248.867982

Enthalpy (298K) = -1248.843900

Free Energy (298K) = -1248.923266

**D**

Number of imaginary frequencies: 0

C 2.84888600 -0.02513300 -1.61694700

O 2.98195000 1.80734100 -3.75227800

C 1.87333100 2.14199500 -3.44576400

O 1.03907300 1.33847700 -2.67372900

C 1.15289600 3.38515200 -3.81549900

H 1.78267400 3.99562700 -4.46345300

H 0.89424500 3.94491000 -2.91001800

H 0.21619700 3.13793300 -4.32554900

C 1.49346300 0.22916800 -2.05994400

C 0.65319300 -0.84286100 -1.77718200

H -0.23977700 -1.03376700 -2.37545300

Au -0.35677700 0.07114500 0.01681200

P -1.48120600 0.82952200 1.90860200

H 3.66620700 0.68189500 -1.66019700

C 2.88672200 -1.29405900 -1.16128400

H 3.75754700 -1.78601700 -0.73704600

C 1.56215000 -1.96687600 -1.29448300

C 1.06423200 -2.71753600 -0.07956400

C 1.53870200 -2.43582700 1.20399300

C 0.03579500 -3.65001500 -0.23364400

C 0.98418000 -3.06861300 2.31453000

H 2.33981100 -1.70934000 1.34145900

C -0.52335600 -4.27654700 0.87531800

H -0.33226800 -3.87679900 -1.23450100

C -0.05147700 -3.98528000 2.15425600

H 1.36338600 -2.84139000 3.30896000

H -1.32375900 -5.00103200 0.74083400

H -0.48328400 -4.47953000 3.02171400

H 1.64387000 -2.68142000 -2.13415500

C -0.94666000 -0.02390400 3.42259500

C -3.28076000 0.58246400 1.84748100

C -1.25942000 2.59849700 2.26309400

H 0.12827300 0.12598600 3.57059300

H -1.49424900 0.37386400 4.28588600

H -1.14630600 -1.09689000 3.32621600

H -3.70123800 1.13539300 1.00142600

H -3.49894500 -0.48284000 1.71906800

H -3.73487600 0.93944900 2.77985800

H -0.19555300 2.81349000 2.40663100

H -1.63227900 3.19243000 1.42243700

H -1.81229200 2.86373500 3.17260500

Electronic Energy (0K) = -1249.2553356

Electronic Energy (0K) + ZPE = -1248.925255

Enthalpy (298K) = -1248.901092

Free Energy (298K) = -1248.980818

**E**

Number of imaginary frequencies: 0

C 2.88031900 -0.29012900 -1.77986900

O 2.62851300 1.53512100 -4.08001000

C 1.82958700 2.09292000 -3.37009000

O 1.17957100 1.46879800 -2.34964400

C 1.39578700 3.51602200 -3.46129700

H 1.89107600 4.00211800 -4.30292800

H 1.64308200 4.04031500 -2.53198600

H 0.30930900 3.56996900 -3.58537600

C 1.51808100 0.16504800 -2.06176500

C 0.63147800 -0.82968700 -1.90195500

H -0.44402700 -0.77610800 -2.02747500

H 3.76325800 0.33743700 -1.83898900

C 2.81586400 -1.58978300 -1.44812300

H 3.64204600 -2.23763700 -1.17280900

C 1.38756800 -2.06644000 -1.50630100

C 0.91102400 -2.70431800 -0.21862900

C 0.91268900 -1.96517800 0.96861300

C 0.46961300 -4.02612600 -0.18918400

C 0.48091700 -2.53754500 2.15867800

H 1.25733800 -0.93098400 0.95066500

C 0.03574700 -4.60279500 1.00364800

H 0.46475400 -4.60874700 -1.10999700

C 0.04012000 -3.86064000 2.17981800

H 0.48764100 -1.95062200 3.07502300

H -0.30683200 -5.63561600 1.01045700

H -0.29856400 -4.30907300 3.11138100

H 1.29862900 -2.81586500 -2.31148300

Electronic Energy (0K) = -652.5929094

Electronic Energy (0K) + ZPE = -652.377725

Enthalpy (298K) = -652.363513

Free Energy (298K) = -652.419771

**H_2_O**

Number of imaginary frequencies: 0

O 2.86590400 3.52996500 0.74730000

H 3.10108500 3.16636000 1.60758000

H 1.94979500 3.25673200 0.63083600

Electronic Energy (0K) = -76.3921609

Electronic Energy (0K) + ZPE = -76.370614

Enthalpy (298K) = -76.366834

Free Energy (298K) = -76.388920

**HOAc**

Number of imaginary frequencies: 0

O 1.67756000 1.13987700 -4.18465200

C 1.20250100 1.93809400 -3.40875600

O 1.34459400 1.82085500 -2.07753600

C 0.40750100 3.14904600 -3.76823900

H 0.29013500 3.21244500 -4.85090400

H 0.91097500 4.04777700 -3.39650200

H -0.57594200 3.10696800 -3.28867300

H 1.87004200 1.01761500 -1.91951300

Electronic Energy (0K) = -228.9725508

Electronic Energy (0K) + ZPE = -228.910757

Enthalpy (298K) = -228.905238

Free Energy (298K) = -228.938054

**F**

Number of imaginary frequencies: 0

C 1.64981400 -0.98215900 -1.61015800

O 1.62511100 1.34522600 -2.29328800

C 0.92756500 0.22750500 -2.00828800

C -0.40257500 -0.00266400 -2.02616700

H -1.18865400 0.70281600 -2.27614200

H 2.73131000 -1.04036700 -1.52762700

C 0.75266400 -1.95525000 -1.38613100

H 0.95321300 -2.97423200 -1.06977100

C -0.64006900 -1.43660700 -1.63958800

C -1.56634900 -1.63178700 -0.45750300

C -1.29916000 -0.99204700 0.75729600

C -2.68741100 -2.45538300 -0.55002300

C -2.13602500 -1.17151300 1.85168700

H -0.42460500 -0.34574400 0.83543700

C -3.53006900 -2.63674500 0.54596200

H -2.90391700 -2.95795500 -1.49250100

C -3.25704100 -1.99560200 1.74932800

H -1.91488100 -0.66693100 2.79031600

H -4.40268700 -3.28072300 0.45558900

H -3.91366300 -2.13441300 2.60565600

H -1.06390000 -1.99442500 -2.49297500

H 1.00673100 2.04907200 -2.53602000

Electronic Energy (0K) = -500.0239025

Electronic Energy (0K) + ZPE = -499.845822

Enthalpy (298K) = -499.835171

Free Energy (298K) = -499.881852

**2a**

Number of imaginary frequencies: 0

C 1.60892700 -0.84875600 -1.60871500

O 1.38157200 1.37716600 -2.50444400

C 0.89292700 0.32601600 -2.13081100

C -0.58942900 -0.00701800 -2.12486100

H -1.10712400 0.69151100 -1.45572700

H 2.68695800 -0.87955300 -1.48302500

C 0.73714600 -1.82954200 -1.34107100

H 0.99155800 -2.81001300 -0.94297900

C -0.69371100 -1.46291100 -1.63926100

C -1.59398600 -1.67324000 -0.44299700

C -1.30348500 -1.05317000 0.77646100

C -2.72292500 -2.48765900 -0.53020400

C -2.12930400 -1.23703000 1.87986500

H -0.41929900 -0.41950900 0.85707100

C -3.55209500 -2.67449500 0.57355100

H -2.95490200 -2.97765800 -1.47550200

C -3.25763500 -2.04941200 1.78134500

H -1.89069000 -0.74718800 2.82183700

H -4.43049200 -3.31105000 0.48811000

H -3.90361900 -2.19450100 2.64460600

H -1.05783400 -2.11186900 -2.44969300

H -1.00760400 0.14523300 -3.12613900

Electronic Energy (0K) = -500.0513436

Electronic Energy (0K) + ZPE = -499.872961

Enthalpy (298K) = -499.862357

Free Energy (298K) = -499.909529

**TS_C-1W_**

Number of imaginary frequencies: 1

C 1.32259100 -0.60592100 1.46230500

O 1.35773500 -1.43458600 2.68358200

C 0.76162800 -2.60349900 2.45657900

O -0.06695800 -2.47486100 1.25040700

C 0.07801400 -3.22484100 3.61517900

H -0.71382000 -2.55400000 3.95899600

H 0.80200000 -3.36988700 4.42206200

H -0.34768400 -4.18983100 3.33083900

C 0.06407500 -1.08859100 0.80144700

C -0.82929000 -0.53127700 0.00384300

H -1.64907000 -1.18781000 -0.29850600

Au -0.71521500 1.42914500 -0.68023600

P -0.65161000 3.63569700 -1.51416700

H 2.21918200 -0.85129000 0.87001700

C 1.31513100 0.82566400 1.83615200

H 0.47235100 1.14961500 2.44871100

C 2.25250400 1.67596700 1.39771300

C 2.28187800 3.12147300 1.61294500

C 1.19350500 3.82997900 2.14573500

C 3.43100900 3.83436300 1.24600700

C 1.26440200 5.20653800 2.31599000

H 0.27974300 3.30258300 2.41737900

C 3.50108200 5.21261600 1.41753300

H 4.27642700 3.29241300 0.82363600

C 2.41764100 5.90279000 1.95370400

H 0.41232500 5.74229700 2.72955200

H 4.40254300 5.74892900 1.12970800

H 2.46737700 6.98122500 2.08586400

H 3.07726500 1.27228200 0.80419700

O 1.63594500 -3.60482000 1.76098300

H 0.83715700 -3.23871300 0.89047300

H 2.55993000 -3.28866600 1.68909400

C -1.38579800 3.81327700 -3.17334700

C 1.01517500 4.35088300 -1.69709000

C -1.54947700 4.86371400 -0.51004300

H -0.84382600 3.18128900 -3.88478500

H -2.43295000 3.49384000 -3.14723100

H -1.33219400 4.85874100 -3.50079500

H 1.51435600 4.39102600 -0.72139600

H 1.60995600 3.72430500 -2.37046700

H 0.94428800 5.36403700 -2.11160400

H -1.48176600 5.85328600 -0.97841200

H -2.60174100 4.57227100 -0.42611000

H -1.11394000 4.90762000 0.49440000

Electronic Energy (0K) = -1325.5483

Electronic Energy (0K) + ZPE = -1325.195391

Enthalpy (298K) = -1325.170226

Free Energy (298K) = -1325.251196

**C-1W**

Number of imaginary frequencies: 0

C 0.98550400 -2.30992400 1.08837800

O 0.09502100 -2.78782100 2.24221600

C 0.03176500 -3.98929400 2.67656200

O 0.74366700 -4.16796400 -0.31231400

C -0.57548900 -4.20361700 3.99851700

H -1.14083100 -3.32667600 4.31498500

H 0.24009400 -4.37743900 4.71232800

H -1.20118300 -5.09907700 3.98272100

C 0.44412100 -2.79562300 -0.21623900

C -0.15538600 -2.09997500 -1.18436600

H -0.42138800 -2.71116900 -2.05626900

Au -0.52425300 -0.06185800 -1.32677900

P -0.91891100 2.24485100 -1.63468600

H 1.96382700 -2.77038800 1.28823100

C 1.02683800 -0.84496700 1.28985100

H 0.05831700 -0.34817500 1.35799900

C 2.17731100 -0.16366000 1.34904200

C 2.30011300 1.28878100 1.46972200

C 1.21594300 2.11317400 1.80899300

C 3.54450700 1.88532900 1.22959000

C 1.37527100 3.48996400 1.89458100

H 0.24252800 1.67216700 2.02217800

C 3.70176700 3.26515400 1.30853700

H 4.39382400 1.25274700 0.97437200

C 2.61771700 4.07212200 1.64133800

H 0.52575800 4.11458100 2.16438900

H 4.67515000 3.71032600 1.11487400

H 2.73923900 5.15075800 1.70992500

H 3.11693000 -0.71401600 1.25493700

O 0.48087100 -5.00371600 2.04684700

H 0.30007600 -4.55481700 -1.08410200

H 0.69418600 -4.80275600 1.06828500

C -1.89094400 2.63617500 -3.12663300

C 0.59064100 3.24823000 -1.83212600

C -1.83674300 3.07567500 -0.29619500

H -1.36560200 2.26680400 -4.01372000

H -2.86854500 2.14619400 -3.06826600

H -2.03183500 3.72062800 -3.21277200

H 1.21819100 3.15758900 -0.93804600

H 1.15609000 2.88721700 -2.69786000

H 0.32587900 4.30188300 -1.98443100

H -1.94970800 4.14298800 -0.52308900

H -2.82738800 2.61944600 -0.19579300

H -1.29966300 2.96111900 0.65169500

Electronic Energy (0K) = -1325.6091076

Electronic Energy (0K) + ZPE = -1325.253317

Enthalpy (298K) = -1325.226964

Free Energy (298K) = -1325.311319

**(H_2_O)_2_**

Number of imaginary frequencies: 0

O -1.99666800 -1.18309000 1.28065900

H -1.16842300 -1.49285800 1.65792400

H -2.15261900 -0.33704500 1.73462300

O -2.41680500 1.29305400 2.62886600

H -2.48428400 1.99256100 1.96749300

H -3.28861000 1.27457000 3.04223700

Electronic Energy (0K) = -152.7940615

Electronic Energy (0K) + ZPE = -152.747252

Enthalpy (298K) = -152.740655

Free Energy (298K) = -152.773516

**TS_C-2W_**

Number of imaginary frequencies: 1

C 4.12597000 1.65369200 1.29046300

O 4.60701000 3.00189700 1.04286600

C 3.82589500 3.54061100 0.08428500

O 3.30797500 2.56380900 -0.70428500

C 4.43595400 4.68514600 -0.64391700

H 5.32074700 4.32383000 -1.17515800

H 4.73655900 5.46405100 0.06215400

H 3.72038700 5.09520900 -1.35993600

C 3.33038200 1.35956800 0.03330000

C 2.70503500 0.25325800 -0.35723000

H 2.16708800 0.34370500 -1.30571400

Au 2.79333900 -1.53803200 0.68970400

P 2.87492400 -3.61357800 1.81239000

C 5.25249200 0.71670800 1.52223100

H 5.96301500 0.60425500 0.70094000

C 5.33217600 -0.02170900 2.63625300

C 6.29971900 -1.08728500 2.89689500

C 6.94149100 -1.77877100 1.85898000

C 6.55981400 -1.46647200 4.22032500

C 7.83170600 -2.80753600 2.14290700

H 6.72306100 -1.52252000 0.82285500

C 7.45647500 -2.49016700 4.50346600

H 6.04825900 -0.94690500 5.03000500

C 8.09483700 -3.16405700 3.46489900

H 8.31790000 -3.34003200 1.32825100

H 7.65216500 -2.76839200 5.53669800

H 8.78913700 -3.97230400 3.68357100

H 4.60507600 0.16442200 3.43285800

O 2.54832700 4.12973100 0.79870200

H 2.79711700 4.55828000 1.63978600

H 1.74777700 3.40077100 0.93931400

O 0.79723900 2.43939200 1.10841100

H -0.04529500 2.64026300 0.67080200

H 1.12265100 1.59850900 0.72726100

C 1.23873900 -4.31646100 2.20362900

C 3.71110900 -4.93694900 0.87837900

C 3.74996100 -3.61437200 3.41175300

H 0.69725400 -3.63671700 2.87013900

H 0.66070900 -4.43835700 1.28150300

H 1.35331300 -5.29112200 2.69353100

H 4.81641800 -3.43202500 3.23684600

H 3.35759600 -2.82229900 4.05935300

H 3.62614000 -4.58595400 3.90570500

H 4.74681200 -4.64024200 0.67768700

H 3.70591200 -5.86950600 1.45594000

H 3.19927800 -5.09688500 -0.07639800

H 3.47190400 1.67878800 2.17799800

Electronic Energy (0K) = -1402.0085054

Electronic Energy (0K) + ZPE = -1401.628427

Enthalpy (298K) = -1401.601070

Free Energy (298K) = -1401.686352

**C-2W**

Number of imaginary frequencies: 0

C -0.04140800 2.24179600 0.08070600

O 0.26803800 3.63946200 -0.12370200

C -0.45597000 4.06450800 -1.19322000

O -0.68244500 3.00544700 -2.03478900

C 0.12040500 5.26266700 -1.86579300

H 1.11287700 5.00745500 -2.24651800

H 0.21203000 6.08491100 -1.15071000

H -0.52319700 5.56992300 -2.69324400

C -0.61528400 1.83257200 -1.26604600

C -1.05333100 0.64834500 -1.69152900

H -1.46463000 0.65358600 -2.70591100

Au -0.85755200 -1.09617600 -0.57885600

P -0.60699300 -3.08437800 0.67238000

C 1.17075600 1.47784300 0.46687200

H 2.01284100 1.53518600 -0.22557400

C 1.18035900 0.67430900 1.53774000

C 2.24128600 -0.25972400 1.91441200

C 3.22353100 -0.68368800 1.00663100

C 2.26015400 -0.78169200 3.21515700

C 4.20790300 -1.58133300 1.40082300

H 3.20421000 -0.32028300 -0.01990300

C 3.24346300 -1.68326800 3.60762600

H 1.49506000 -0.46433100 3.92365100

C 4.22283700 -2.08287300 2.70213000

H 4.96250500 -1.90176300 0.68574200

H 3.24536500 -2.07411500 4.62272900

H 4.99182100 -2.78980200 3.00529300

H 0.30872200 0.69016100 2.19952800

O -1.84674600 4.43570400 -0.70943300

H -1.79020200 4.93472400 0.12657000

H -2.62694800 3.54504500 -0.68838700

O -3.37479300 2.57688700 -0.67256600

H -4.12891900 2.66258000 -1.28145800

H -2.85684200 1.78176000 -0.94032900

C 1.05125000 -3.83772000 0.59086500

C -0.89677400 -2.88471300 2.46140400

C -1.72683200 -4.44214200 0.19710900

H 1.29435100 -4.08219800 -0.44863900

H 1.79799200 -3.12813800 0.96795700

H 1.07901500 -4.75211400 1.19632900

H -2.76655700 -4.11995800 0.31739000

H -1.56004100 -4.70535400 -0.85272100

H -1.54309400 -5.32171300 0.82625500

H -1.90943900 -2.50491200 2.63334600

H -0.77595600 -3.84697500 2.97409500

H -0.17443500 -2.16675400 2.86803300

H -0.80707500 2.15710600 0.87065700

Electronic Energy (0K) = -1402.0092702

Electronic Energy (0K) + ZPE = -1401.628707

Enthalpy (298K) = -1401.601064

Free Energy (298K) = -1401.687048

**(H_2_O)_3_**

Number of imaginary frequencies: 0

O 1.33244100 -4.66068300 -0.08040100

H 0.93876800 -4.59046700 0.79677600

H 2.03444700 -3.97871600 -0.09025600

O 2.78341200 -2.35550200 -0.60481400

H 3.48683900 -2.50608700 -1.24637800

H 1.97692700 -2.23216000 -1.14566600

O 0.31883800 -2.74849600 -1.81710800

H 0.42166700 -3.54924000 -1.26368600

H 0.41550800 -3.06646300 -2.72200900

Electronic Energy (0K) = -229.2058069

Electronic Energy (0K) + ZPE = -229.131079

Enthalpy (298K) = -229.123291

Free Energy (298K) = -229.159656

**B-3W**

Number of imaginary frequencies: 0

O 0.04641000 -1.35468500 4.31753700

H 0.02654300 -0.87713700 5.15655100

H -0.73808900 -1.91791000 4.33844700

O 2.43120000 -2.51296300 3.36189500

H 2.67667800 -1.63765600 3.01182000

H 1.64502200 -2.29437700 3.89257100

O 2.45626600 0.19938900 2.39480500

H 2.29709900 0.16679600 1.43771600

H 1.61780100 0.49961700 2.76938700

C 0.24457100 -2.09290200 1.15621800

O 0.31164800 -3.43518800 1.78669800

C 1.47149700 -3.93345400 1.53979900

O 2.17172000 -3.32046300 0.65338100

C 1.89808800 -5.21112300 2.10420000

H 1.61125600 -6.00920000 1.40792400

H 1.40392200 -5.38093500 3.06201300

H 2.98372800 -5.21813600 2.21649100

C 1.47696100 -2.10625900 0.28820200

C 1.95716500 -1.23830900 -0.58384900

H 2.91627800 -1.49412600 -1.03873200

Au 0.94967000 0.54797200 -0.94698100

P -0.20067600 2.58340400 -1.27224100

H 0.35288600 -1.39538100 1.99775200

C -1.02980700 -1.87277400 0.44159200

H -1.24030200 -2.52755000 -0.40522400

C -1.79899600 -0.81873600 0.74958800

C -2.96685100 -0.34174900 0.01332300

C -3.26970700 -0.78180700 -1.28417500

C -3.79135400 0.62398400 0.60656200

C -4.37838400 -0.28255700 -1.95520900

H -2.62270100 -1.50673100 -1.77571400

C -4.89909300 1.12551200 -0.06760100

H -3.55584600 0.97560600 1.61094400

C -5.19683200 0.67099200 -1.34943500

H -4.60151200 -0.63083200 -2.96121800

H -5.53048700 1.87251500 0.40802000

H -6.06076600 1.06352100 -1.88089800

H -1.52104800 -0.22093900 1.62415100

C -1.56801000 2.51947700 -2.47505300

C 0.82999400 3.97263200 -1.84331100

C -0.98796900 3.20836500 0.24817200

H -1.18031600 2.23778600 -3.45968500

H -2.30281000 1.76879500 -2.15916400

H -2.05463700 3.50024700 -2.54238100

H 1.63046500 4.15786300 -1.11936200

H 1.28090800 3.72231900 -2.80931500

H 0.21843300 4.87683500 -1.94999600

H -1.48465400 4.16665800 0.05241400

H -1.73187300 2.48126200 0.59628000

H -0.23095400 3.34269400 1.02853800

Electronic Energy (0K) = -1478.4262343

Electronic Energy (0K) + ZPE = -1478.021037

Enthalpy (298K) = -1477.987844

Free Energy (298K) = -1478.085434

**TS_C-3W_**

Number of imaginary frequencies: 1

O -0.26809500 0.43132200 2.50668100

H -0.18000200 0.03065800 3.38141000

H -1.00125900 -0.03695600 2.08625500

O 1.79176800 0.37430000 0.81610100

H 2.05085800 1.30942600 0.60634000

H 1.09782600 0.41002400 1.52686900

O 2.38472000 2.94540800 0.13883900

H 2.10089500 3.07395600 -0.78093800

H 1.92233200 3.62382700 0.64794600

C -0.36707100 1.37244100 -1.14308000

O -0.30292800 0.04716400 -0.52289400

C 0.93148500 -0.39605900 -0.65655200

O 1.58594200 0.18363500 -1.64258100

C 1.19819500 -1.81377000 -0.35166600

H 0.73409100 -2.42065300 -1.13687100

H 0.75484700 -2.07764000 0.61150000

H 2.27297800 -1.99931500 -0.33513100

C 0.88297300 1.37503000 -1.99720700

C 1.34750900 2.25655100 -2.87041100

H 2.31618900 2.00984800 -3.31252800

Au 0.31147200 4.00287900 -3.31142900

P -0.89082600 5.97770200 -3.79304700

H -0.29524400 2.10826800 -0.32569600

C -1.62539700 1.55378100 -1.90197100

H -1.80920300 0.83917300 -2.70595800

C -2.41295000 2.61785800 -1.69711200

C -3.56188700 3.01414100 -2.51029600

C -3.79017500 2.48585100 -3.78996500

C -4.44644600 3.98269100 -2.01615200

C -4.88552000 2.89822800 -4.53765300

H -3.09501300 1.75976300 -4.20900900

C -5.54091500 4.39763700 -2.76717200

H -4.27029800 4.40376100 -1.02630100

C -5.76547100 3.85342000 -4.02877100

H -5.04943200 2.48046000 -5.52856000

H -6.21974400 5.14699700 -2.36626400

H -6.61917500 4.17768000 -4.61956300

H -2.17381400 3.28303600 -0.86140600

C 0.11020000 7.36384800 -4.42403100

C -1.75795100 6.68080000 -2.35181500

C -2.20743100 5.78390500 -5.03838500

H 0.87617600 7.62953200 -3.68794000

H 0.60578900 7.06456000 -5.35362500

H -0.52803500 8.23496700 -4.61611500

H -2.47613200 5.94752300 -1.96514900

H -1.03507400 6.91757700 -1.56390000

H -2.29429600 7.59317000 -2.64048000

H -2.73075000 6.73690400 -5.18417500

H -1.76929100 5.46077100 -5.98870500

H -2.92202500 5.02128500 -4.70527500

Electronic Energy (0K) = -1478.4216747

Electronic Energy (0K) + ZPE = -1478.016022

Enthalpy (298K) = -1477.984337

Free Energy (298K) = -1478.079847

**C-3W**

Number of imaginary frequencies: 0

O 0.41692300 -2.80197400 4.99207500

H 0.52527100 -3.28881600 5.82162200

H -0.40992400 -3.12119900 4.60230500

O 2.22946600 -2.93900300 3.21971200

H 2.64746300 -2.01730900 3.03301800

H 1.55112700 -2.89503500 3.99168300

O 3.24007200 -0.64526200 2.67711400

H 2.94114100 -0.40022200 1.78256000

H 2.98322300 0.08797300 3.25405400

C 0.18512300 -1.79798700 1.41687800

O 0.23355400 -3.11618100 2.01578100

C 1.53136400 -3.52427700 1.98237600

O 2.16091200 -2.94657100 0.91899400

C 1.69302400 -5.00117400 2.08029300

H 1.20443100 -5.46669100 1.22042100

H 1.22380800 -5.36261200 3.00041300

H 2.75425500 -5.25977100 2.08723000

C 1.44646500 -1.78150700 0.57258100

C 1.89397900 -0.87805500 -0.29495000

H 2.87179900 -1.10701100 -0.72937800

Au 0.82766900 0.83426900 -0.78957400

P -0.38395000 2.78815700 -1.33567400

H 0.24438800 -1.04450200 2.22181100

C -1.06185300 -1.61763400 0.63547300

H -1.23700700 -2.35056900 -0.15427000

C -1.85690500 -0.55425500 0.80812700

C -3.00063700 -0.18541800 -0.02636100

C -3.20235400 -0.73210300 -1.30276400

C -3.90935600 0.77267900 0.44340600

C -4.29467400 -0.34863900 -2.07027900

H -2.48826400 -1.45013100 -1.70363000

C -5.00043300 1.15952700 -0.32755500

H -3.75481900 1.20879100 1.43044600

C -5.19852000 0.59668000 -1.58550000

H -4.43710500 -0.78108800 -3.05828500

H -5.69768800 1.90145700 0.05529300

H -6.04952500 0.89873100 -2.19178000

H -1.63345600 0.12945400 1.63323300

C 0.62454700 4.16316200 -1.98083600

C -1.27933000 3.52860700 0.06989600

C -1.67841800 2.56469700 -2.59972900

H 1.37296100 4.45102200 -1.23493400

H 1.14205500 3.84290600 -2.89128600

H -0.01231900 5.02663900 -2.20890800

H -2.01536900 2.81140000 0.45292000

H -0.57420200 3.77432200 0.87102700

H -1.79887200 4.44011900 -0.25051900

H -2.20036600 3.51304700 -2.77743100

H -1.22269500 2.22009400 -3.53415500

H -2.39795400 1.80857400 -2.26209700

Electronic Energy (0K) = -1478.4252078

Electronic Energy (0K) + ZPE = -1478.018746

Enthalpy (298K) = -1477.987751

Free Energy (298K) = -1478.081180

**(H_2_O)_4_**

Number of imaginary frequencies: 0

O 6.38769900 3.82080000 -1.14638000

H 5.97947900 3.91474100 -0.27872900

H 7.13532100 3.19526300 -1.00643900

O 8.29934100 1.87739300 -0.92290700

H 8.28835000 1.47502800 -0.04752200

H 7.85458800 1.21968400 -1.50570500

O 6.83088700 0.24048700 -2.54160800

H 6.07770600 0.86245800 -2.66678200

H 7.23416400 0.16631400 -3.41364200

O 4.90769000 2.16951500 -2.74691300

H 4.12153900 1.92587100 -2.24598800

H 5.36289000 2.84321100 -2.19128200

Electronic Energy (0K) = -305.61588

Electronic Energy (0K) + ZPE = -305.515753

Enthalpy (298K) = -305.505397

Free Energy (298K) = -305.549499

**TS_C-4W_**

Number of imaginary frequencies: 1

O 0.02850200 3.89136100 0.86179900

H -0.03561800 4.73288400 1.32991800

H -0.24803600 3.20581500 1.49621400

O -0.33085400 1.42370300 2.19776400

H -0.25568200 1.42340200 3.16401300

H -1.17653000 0.98835000 2.01152000

O 1.72472800 0.34028900 0.75538100

H 2.10019700 1.23383400 0.48190000

H 1.00292600 0.56549700 1.39056900

O 2.43053500 2.80902200 0.09541700

H 2.39208700 2.86926500 -0.87086700

H 1.65458800 3.33068200 0.39866900

C -0.37497500 1.28251700 -1.23732700

O -0.31954000 -0.02466900 -0.58000300

C 0.92690200 -0.45817400 -0.67236400

O 1.58676600 0.09045800 -1.68072300

C 1.19131800 -1.87535200 -0.35330200

H 0.75693400 -2.48746800 -1.15095400

H 0.72300900 -2.14050000 0.59698100

H 2.26669000 -2.05363300 -0.30397600

C 0.88467000 1.26928000 -2.07307500

C 1.35373000 2.14637700 -2.94839400

H 2.32850500 1.90794700 -3.38167800

Au 0.32666600 3.91938800 -3.30194100

P -0.85948900 5.93331500 -3.63293900

H -0.30684500 2.04645900 -0.44785400

C -1.63001900 1.45577200 -2.00429100

H -1.80262700 0.75473400 -2.82249600

C -2.43330800 2.50302000 -1.77306300

C -3.58885200 2.90687600 -2.57240600

C -3.79957000 2.43203500 -3.87570000

C -4.49574000 3.83350700 -2.03991300

C -4.89999800 2.85490700 -4.61030400

H -3.08655100 1.74029600 -4.32207200

C -5.59650000 4.25691900 -2.77667900

H -4.33194900 4.21465700 -1.03199600

C -5.80318000 3.76604100 -4.06309200

H -5.04997200 2.47974200 -5.62030400

H -6.29361400 4.97241200 -2.34629500

H -6.66118400 4.09817100 -4.64321900

H -2.19835900 3.14966400 -0.92165100

C 0.14409300 7.35807600 -4.16593500

C -1.70902500 6.52958600 -2.13381300

C -2.19026000 5.84329500 -4.87578400

H 0.91763500 7.56282400 -3.41836400

H 0.63031800 7.12807400 -5.11984800

H -0.49100400 8.24433400 -4.28541600

H -2.44389500 5.78174500 -1.81270100

H -0.98028800 6.67803700 -1.32946400

H -2.22388100 7.47620500 -2.33946200

H -2.71184500 6.80611400 -4.94142000

H -1.76327100 5.59332300 -5.85285900

H -2.90370900 5.05897100 -4.59486900

Electronic Energy (0K) = -1554.8363905

Electronic Energy (0K) + ZPE = -1554.403286

Enthalpy (298K) = -1554.370116

Free Energy (298K) = -1554.467315

**C-4W**

Number of imaginary frequencies: 0

O 1.07671900 0.59862300 3.61370800

H 1.28343900 1.32494500 4.22245900

H 0.68942300 -0.14510800 4.13741900

O 0.32088900 -1.78692600 4.64031300

H 0.30926300 -1.92267800 5.60010400

H -0.53653200 -2.11165700 4.32411100

O 2.19609100 -2.88893200 2.90451100

H 2.82662700 -1.34149000 2.55525000

H 1.58383500 -2.77538300 3.66065600

O 2.88480600 -0.36708000 2.32859200

H 2.61754500 -0.32738100 1.37208000

H 2.09652800 0.14256700 2.93577800

C 0.12057300 -1.91169500 1.19457300

O 0.15853600 -3.17489500 1.87006500

C 1.49181800 -3.57305100 1.87204300

O 2.04080000 -3.11948000 0.66628600

C 1.61990000 -5.05677500 2.01075400

H 1.10185000 -5.55411500 1.18651000

H 1.17897500 -5.37615400 2.95964300

H 2.67734100 -5.33380700 2.00070800

C 1.38888400 -1.94207900 0.35170700

C 1.87004200 -1.00174200 -0.46813300

H 2.83878100 -1.23844700 -0.91924700

Au 0.84592900 0.76730700 -0.83798200

P -0.34735200 2.76857700 -1.23473700

H 0.18384200 -1.09248000 1.93154800

C -1.12531300 -1.74416700 0.40425200

H -1.30362600 -2.48344400 -0.37874700

C -1.92306200 -0.68195100 0.57523100

C -3.06674900 -0.30759300 -0.25659500

C -3.24343500 -0.81471900 -1.55270300

C -3.99643700 0.62041100 0.23276400

C -4.33203100 -0.42272500 -2.32161000

H -2.51253500 -1.50871100 -1.96563200

C -5.08494300 1.01356600 -0.53834800

H -3.86060200 1.02686800 1.23514200

C -5.25772600 0.49033400 -1.81714100

H -4.45480300 -0.82370800 -3.32542900

H -5.79941900 1.73078400 -0.14029800

H -6.10601800 0.79881300 -2.42402200

H -1.69826500 0.00047700 1.40218300

C 0.66077700 4.19317700 -1.76137700

C -1.25083100 3.38722300 0.22370800

C -1.63787700 2.64689100 -2.51725100

H 1.40522800 4.42084000 -0.99115200

H 1.18314900 3.94901300 -2.69243300

H 0.02264100 5.07079900 -1.92165100

H -1.99644500 2.64360900 0.52906700

H -0.55242100 3.54863700 1.05202700

H -1.75903000 4.32969300 -0.01476800

H -2.16848300 3.60230400 -2.61240100

H -1.17761700 2.38922500 -3.47711900

H -2.35044300 1.85757500 -2.24820600

Electronic Energy (0K) = -1554.8436981

Electronic Energy (0K) + ZPE = -1554.411739

Enthalpy (298K) = -1554.379031

Free Energy (298K) = -1554.477899

**TS_3W_**

Number of imaginary frequencies: 1

C 3.27800000 3.34779700 -2.40654900

O 3.44566000 4.58849400 -1.68898400

C 3.47113500 4.57133800 -0.33270000

O 3.28572200 3.56572000 0.31562700

C 3.73120800 5.93346500 0.20386900

H 2.83015900 6.53662200 0.03957100

H 4.56225700 6.41079000 -0.32297400

H 3.93752800 5.87750900 1.27363400

C 1.91631200 3.27424400 -2.95694900

C 1.01352900 3.30864100 -3.83410900

H -0.05998000 3.16528800 -3.83248000

Au 1.95093700 3.82570100 -5.69758400

H 3.45547400 2.50205700 -1.73025500

C 4.26242600 3.35111800 -3.53729500

H 4.35505300 4.30564500 -4.06165700

C 4.91125900 2.25079200 -3.93372600

C 5.79336800 2.15749600 -5.09637400

C 6.25733300 3.28900700 -5.78476300

C 6.16972400 0.89032500 -5.55951700

C 7.05829100 3.15163700 -6.91048100

H 6.00133500 4.28585100 -5.42679900

C 6.96886900 0.75317100 -6.68991600

H 5.82233700 0.00708400 -5.02481000

C 7.41394800 1.88292100 -7.37008000

H 7.41135000 4.03925700 -7.43196100

H 7.24724400 -0.23912900 -7.03763700

H 8.04170800 1.77850100 -8.25202700

H 4.74422200 1.31981300 -3.38620800

P 2.79950600 4.31958200 -7.81637000

C 3.61718100 2.90318300 -8.61115700

C 4.03551100 5.65347100 -7.84288300

C 1.52644700 4.84457700 -9.00333100

H 4.38307300 5.81436800 -8.87078900

H 3.58858600 6.57681500 -7.45995500

H 4.88705600 5.38076700 -7.20987600

H 2.90276300 2.07997800 -8.71510100

H 3.98321500 3.19756300 -9.60249200

H 4.46012500 2.56852600 -7.99579100

H 0.77644600 4.05412200 -9.11050500

H 1.03527800 5.75300100 -8.64003000

H 1.98970300 5.04381000 -9.97735700

O 0.88311600 2.94711500 -1.04261500

H 1.55236100 2.82553500 -0.35049400

H 0.62812900 3.89391300 -0.95634100

O 0.64780400 5.65238000 -0.94729000

H 0.97817800 6.06841600 -1.76943800

H -0.12031300 6.16627800 -0.67396000

O 1.99081000 6.64080100 -3.18294900

H 1.72054600 6.11986400 -3.95408500

H 2.77918800 6.18180600 -2.85549100

Electronic Energy (0K) = -1478.4133622

Electronic Energy (0K) + ZPE = -1478.009474

Enthalpy (298K) = -1477.976988

Free Energy (298K) = -1478.073573

**P_3W_**

Number of imaginary frequencies: 0

C 0.15245200 -0.45025500 2.54262800

O 0.58111400 0.63650700 3.43655900

C 0.42866800 0.48712500 4.75607600

O -0.24848700 -0.40123000 5.25216300

C 1.14781600 1.54040000 5.52046300

H 0.77468500 2.52493400 5.21674100

H 2.21667700 1.50958900 5.28698600

H 0.99063300 1.40005300 6.59050000

C -1.29701000 -0.25309800 2.19979600

C -1.88808000 -0.03704200 1.02700200

H -2.97619600 0.06576300 1.11038300

Au -1.04916700 0.15381500 -0.86296200

H 0.28056000 -1.39702600 3.08969100

C 1.07964400 -0.39352200 1.38214700

H 1.17625100 0.58348400 0.90466300

C 1.75823800 -1.45811600 0.93957800

C 2.65582000 -1.48514500 -0.21594400

C 3.06326800 -0.32218300 -0.88845700

C 3.12490800 -2.72162800 -0.67745100

C 3.90616600 -0.40087500 -1.98933200

H 2.72895700 0.65446600 -0.53921200

C 3.96562400 -2.80055700 -1.78298500

H 2.82100800 -3.62907600 -0.15668100

C 4.35888100 -1.64027700 -2.44335100

H 4.21787600 0.51076100 -2.49568000

H 4.31750300 -3.77086900 -2.12636900

H 5.02009900 -1.69757000 -3.30501600

H 1.61783000 -2.41476500 1.44924700

P -0.26474900 0.37844600 -3.07965800

C 0.59950100 -1.07661000 -3.75666400

C 0.89066000 1.75714100 -3.37552800

C -1.60865600 0.67637600 -4.27490200

H 1.18262200 1.78226100 -4.43258500

H 0.40713600 2.70347200 -3.11021200

H 1.78519600 1.63108800 -2.75567400

H -0.05339100 -1.95352300 -3.69355300

H 0.86585300 -0.89667900 -4.80556600

H 1.51199800 -1.27037200 -3.18070300

H -2.31404900 -0.16098300 -4.24792300

H -2.14288700 1.59389100 -4.00621000

H -1.20034000 0.77571400 -5.28806300

O -2.11086100 -0.23085900 3.38193100

H -1.59054600 -0.56799000 4.15598900

H -2.36086400 0.99146400 3.56185400

O -2.40719200 2.13634000 3.62314800

H -1.90050000 2.54504500 2.84332900

H -3.31444100 2.48251100 3.64198500

O -1.03804600 2.99016800 1.59513100

H -1.03703600 2.22957300 0.98477800

H -0.10918300 3.12645600 1.83182600

Electronic Energy (0K) = -1478.4361964

Electronic Energy (0K) + ZPE = -1478.029665

Enthalpy (298K) = -1477.999093

Free Energy (298K) = -1478.091500

**TS_6-endo-dig_**

Number of imaginary frequencies: 1

C 3.60068400 3.29228600 -2.86534300

O 3.96267200 3.76977200 -1.54368100

C 3.03614300 4.16762300 -0.67804800

O 1.83765700 4.25261400 -0.93857300

C 3.61559900 4.50232600 0.64836300

H 4.40881500 5.24726200 0.52713700

H 4.07127200 3.60761300 1.08506000

H 2.83853700 4.88483700 1.31116900

C 2.57527500 4.18892700 -3.49281700

C 1.60096900 4.75333000 -2.92430000

H 0.72512300 5.35610800 -2.75697300

Au 2.60902800 4.64789200 -5.57554700

H 3.14315200 2.29848300 -2.74753600

C 4.85188700 3.20816200 -3.66755000

H 5.38037700 4.15196200 -3.81551600

C 5.24883900 2.07040100 -4.24779300

C 6.36553000 1.92675600 -5.18236400

C 6.94003100 3.02638400 -5.83728500

C 6.86509000 0.64714900 -5.45647000

C 7.99482200 2.84854700 -6.72279600

H 6.54393600 4.02720500 -5.66644400

C 7.92418500 0.47021700 -6.33952900

H 6.41534300 -0.21331600 -4.96238600

C 8.49383800 1.57087100 -6.97372900

H 8.42542700 3.71060100 -7.22814800

H 8.30315900 -0.53029700 -6.53608900

H 9.31772300 1.43439300 -7.67064500

H 4.69240700 1.15508100 -4.02825100

P 2.86537400 5.01657800 -7.87022900

C 4.47395900 4.43885200 -8.49365200

C 2.77236400 6.76045900 -8.37724100

C 1.63581000 4.16734500 -8.90643700

H 2.92636900 6.83778500 -9.46046100

H 1.78995000 7.16817400 -8.11837300

H 3.54423200 7.33701400 -7.85732000

H 4.58689700 3.36930500 -8.28635500

H 4.53453400 4.61009600 -9.57544200

H 5.28037600 4.98630900 -7.99396800

H 1.68525400 3.08859500 -8.72648300

H 0.63201300 4.52375500 -8.65371300

H 1.84034900 4.37111700 -9.96459700

Electronic Energy (0K) = -1249.1759433

Electronic Energy (0K) + ZPE = -1248.848869

Enthalpy (298K) = -1248.824003

Free Energy (298K) = -1248.907124

**P_6-endo-dig_**

Number of imaginary frequencies: 0

C -0.06126200 -0.75557600 1.82594100

O -0.03783300 -0.86980500 3.36281200

C -0.38880300 0.08952400 4.12076300

O -0.91554400 1.17951900 3.69397100

C -0.16252000 -0.03935700 5.56729400

H 0.88268700 0.22710200 5.77067000

H -0.31100800 -1.07595400 5.87849900

H -0.81481400 0.63742700 6.12102100

C -0.81756900 0.44209000 1.38880700

C -1.18427200 1.34295600 2.28774600

H -1.71223600 2.27902200 2.15653000

Au -1.15354200 0.67042200 -0.65879000

H -0.56245200 -1.68929700 1.54736400

C 1.35926900 -0.75392700 1.39686700

H 1.91162300 0.16885400 1.58605200

C 1.91192800 -1.81556400 0.79271700

C 3.28011900 -1.93773700 0.29629400

C 4.22367500 -0.90384600 0.39761500

C 3.66841200 -3.14464100 -0.30055100

C 5.51353200 -1.07914400 -0.08241700

H 3.94677300 0.04381600 0.85594000

C 4.96075100 -3.31916400 -0.78174100

H 2.94113700 -3.95171300 -0.38191600

C 5.88726500 -2.28647900 -0.67313500

H 6.23509600 -0.26964500 0.00357500

H 5.24480700 -4.26303600 -1.24159900

H 6.89969300 -2.41877600 -1.04811000

H 1.28320300 -2.69747800 0.64265600

P -1.51191300 0.92107800 -2.97207000

C -0.50171300 -0.18518000 -4.00833300

C -1.14950700 2.58589100 -3.61626400

C -3.22068400 0.59911100 -3.51455900

H -1.33599000 2.61882600 -4.69658800

H -1.78723200 3.32134700 -3.11482400

H -0.10165500 2.83628900 -3.42049200

H -0.72360900 -1.22802400 -3.75851700

H -0.72096600 -0.01019200 -5.06869600

H 0.56043800 0.00364200 -3.82052800

H -3.50184100 -0.42728100 -3.25676200

H -3.90384000 1.28913800 -3.00841800

H -3.30146100 0.73721700 -4.59964100

Electronic Energy (0K) = -1249.2000035

Electronic Energy (0K) + ZPE = -1248.869533

Enthalpy (298K) = -1248.844901

Free Energy (298K) = -1248.928204

**In the absence of TA**

**A*_syn_*_-PMe3_ as the precursor**

**TS*_syn_*_-5-exo-dig_**

Number of imaginary frequencies: 1

C 3.45985600 3.56474800 -2.39695500

O 3.83372000 4.10007500 -1.10480900

C 2.76849800 4.51103000 -0.42702600

O 1.66743600 4.50548900 -0.97419500

C 3.04462600 4.93699300 0.96207300

H 2.17952400 5.45427600 1.37780800

H 3.92880200 5.58045000 0.98626500

H 3.26496900 4.04934100 1.56560300

C 2.31302700 4.31599800 -2.92262600

C 1.54089200 4.87671300 -3.75605700

H 1.82474000 4.95417400 -4.80817400

Au -0.35346200 5.69305700 -3.30833900

H 3.18914100 2.50910000 -2.24999700

C 4.59355300 3.70875600 -3.35352400

H 4.97788700 4.72113200 -3.48055000

C 5.06495900 2.65537100 -4.03338900

C 6.13673500 2.65937400 -5.02489600

C 6.72314000 3.83860200 -5.50859900

C 6.59799100 1.42941400 -5.51259900

C 7.74712800 3.78199200 -6.44308100

H 6.36941000 4.80634400 -5.15774900

C 7.62480500 1.37374600 -6.44763100

H 6.14186300 0.51102600 -5.14507100

C 8.20275600 2.55056400 -6.91447100

H 8.19226800 4.70346500 -6.81167100

H 7.97280300 0.41049200 -6.81364300

H 9.00489300 2.51140500 -7.64810200

H 4.62129300 1.67704900 -3.83244100

P -2.46531000 6.60372300 -2.88434200

C -2.78243100 6.97290200 -1.13165300

C -3.84612800 5.52847500 -3.38097900

C -2.77519300 8.17598900 -3.74553800

H -4.79931200 6.02299600 -3.15737300

H -3.78511200 5.32219700 -4.45444700

H -3.78947800 4.58164400 -2.83420600

H -2.04219000 7.69243700 -0.76705100

H -3.78856400 7.39447300 -1.01777400

H -2.70332800 6.05377700 -0.54196600

H -2.03534100 8.91924400 -3.43126700

H -2.69011000 8.02644100 -4.82676500

H -3.78199000 8.53879400 -3.50522100

Electronic Energy (0K) = -1249.1791091

Electronic Energy (0K) + ZPE = -1248.852429

Enthalpy (298K) = -1248.827546

Free Energy (298K) = -1248.911130

**P*_syn_*_-5-exo-dig_**

Number of imaginary frequencies: 0

C 2.17758400 -1.62096400 1.16658100

O 2.70922500 -1.12190400 2.48476600

C 1.81600700 -0.38643800 3.03641000

O 0.74182000 -0.20170400 2.36170800

C 2.00048600 0.18765000 4.36648600

H 1.45949900 1.13246300 4.44915800

H 3.06368000 0.31479400 4.57827500

H 1.58208800 -0.51661600 5.09766300

C 0.87711100 -0.86743300 1.07644300

C -0.03239300 -0.71695800 0.13320000

H 0.23370300 -1.26630600 -0.77533300

Au -1.79903100 0.36157200 0.22223400

H 2.03957700 -2.69872200 1.32227600

C 3.14660800 -1.34344100 0.08620900

H 3.31443600 -0.29191900 -0.14976500

C 3.73498000 -2.34223200 -0.58715900

C 4.66998200 -2.22577400 -1.70244000

C 5.10472800 -0.99111300 -2.20805700

C 5.15677600 -3.40153400 -2.28901400

C 5.99878000 -0.94130200 -3.26770200

H 4.74246300 -0.06326600 -1.76943400

C 6.05209300 -3.35056900 -3.35096700

H 4.82292600 -4.36309400 -1.90099600

C 6.47556000 -2.11957500 -3.84275900

H 6.32834700 0.02221800 -3.64996600

H 6.41977500 -4.27291100 -3.79478300

H 7.17605400 -2.07512400 -4.67372400

H 3.49530500 -3.36616600 -0.28870500

P -3.82561000 1.56484600 0.29728500

C -4.42531300 1.93939800 1.97687700

C -5.23409800 0.72261500 -0.49437800

C -3.77027600 3.19215200 -0.52115700

H -6.13386700 1.34585800 -0.42326000

H -5.00480500 0.53451500 -1.54846300

H -5.41564600 -0.23595600 0.00288000

H -3.67790400 2.53689500 2.50946200

H -5.36751700 2.49844800 1.92413300

H -4.58677900 1.00630100 2.52676100

H -3.00384000 3.81569800 -0.04906000

H -3.51676300 3.06305000 -1.57860800

H -4.74504300 3.68810400 -0.43801800

Electronic Energy (0K) = -1249.2009702

Electronic Energy (0K) + ZPE = -1248.870606

Enthalpy (298K) = -1248.846132

Free Energy (298K) = -1248.929106

**TS*_syn_*_-3W_**

Number of imaginary frequencies: 1

C 2.73018600 3.53127400 -2.13879600

O 3.84067000 3.15374400 -2.98310100

C 4.47019600 4.11347600 -3.68862800

O 4.14537400 5.28374600 -3.67345600

C 5.58692700 3.53741200 -4.48707500

H 5.15352600 3.04404000 -5.36915800

H 6.13061300 2.77871100 -3.91809100

H 6.26082600 4.33073100 -4.81511300

C 1.49657700 3.29081400 -2.93177600

C 0.79309700 3.09603100 -3.95907000

H -0.26993300 2.94468400 -4.10767400

Au 1.93952100 3.42318000 -5.73604200

H 2.80077000 4.60219100 -1.89719100

C 2.83107400 2.70421300 -0.89767200

H 2.74022500 1.62603300 -1.03440700

C 3.05921900 3.27221000 0.29382600

C 3.22940200 2.59909900 1.58053700

C 3.13430300 1.20793500 1.73416300

C 3.50464100 3.38156600 2.70969200

C 3.31276200 0.62400600 2.98025500

H 2.91769100 0.57672400 0.87433200

C 3.68437700 2.79570000 3.95753900

H 3.57908000 4.46269400 2.59797900

C 3.58907400 1.41440500 4.09598000

H 3.23573000 -0.45597900 3.08498600

H 3.89936300 3.41895700 4.82267100

H 3.72853600 0.95176600 5.07047100

H 3.13079100 4.36333900 0.32940500

P 3.02651400 3.93417700 -7.73863600

C 4.08813500 2.61522200 -8.39942800

C 4.10882100 5.38879100 -7.60382200

C 1.87631300 4.32947500 -9.08938800

H 4.54176600 5.62229900 -8.58421600

H 3.52865300 6.24639800 -7.24595700

H 4.91539700 5.18390800 -6.89167400

H 3.49167400 1.71354900 -8.57146500

H 4.53546400 2.94388200 -9.34543900

H 4.88375200 2.38483800 -7.68313800

H 1.22954400 3.46820300 -9.28566500

H 1.25390500 5.18353600 -8.80319000

H 2.44100600 4.57651400 -9.99654100

O 0.12870000 3.62626800 -1.32983000

H 0.48610400 3.28890000 -0.49570800

H 0.28081900 4.60419800 -1.29842500

O 0.94843400 6.16636400 -1.48375900

H 1.08161600 6.39026200 -2.42982700

H 0.51602100 6.92877400 -1.08328900

O 1.63667200 6.51710700 -4.13246700

H 1.18684200 5.86370700 -4.68715600

H 2.53277900 6.15248300 -4.01746700

Electronic Energy (0K) = -1478.4083154

Electronic Energy (0K) + ZPE = -1478.004672

Enthalpy (298K) = -1477.972240

Free Energy (298K) = -1478.070236

**P*_syn_*_-3W_**

Number of imaginary frequencies: 0

C 0.29940400 0.28021200 1.86260800

O 1.26075600 -0.09241300 0.86394300

C 1.87058200 0.85593500 0.15469500

O 1.61962900 2.04961800 0.24456600

C 2.94453300 0.29158000 -0.70871700

H 2.66401800 -0.69428800 -1.09066700

H 3.84958400 0.17011500 -0.10073700

H 3.16747200 0.97879900 -1.52809300

C -1.11181900 0.29758200 1.31048800

C -1.55659300 0.21926300 0.05410600

H -2.65044600 0.28833200 0.00770500

Au -0.55747100 0.16300900 -1.75761400

H 0.54415700 1.28807100 2.23814900

C 0.43424300 -0.70865500 2.97861300

H 0.31062400 -1.75575300 2.69532500

C 0.65948100 -0.33138200 4.24460200

C 0.77589800 -1.19742100 5.41802900

C 0.59343300 -2.58697600 5.36092400

C 1.08799200 -0.61257700 6.65194100

C 0.72683700 -3.36221300 6.50416100

H 0.34180400 -3.06556700 4.41625900

C 1.22096800 -1.38950200 7.79721900

H 1.22931400 0.46639200 6.70429700

C 1.04136400 -2.76754900 7.72617700

H 0.58203300 -4.43872500 6.44471100

H 1.46480800 -0.91744400 8.74639500

H 1.14338300 -3.37918200 8.61983500

H 0.78208100 0.73755200 4.44383300

P 0.48749900 0.21595700 -3.88071900

C 1.76430400 -1.04735700 -4.19013900

C 1.33029700 1.79368500 -4.23505800

C -0.66675300 0.01287200 -5.27674000

H 1.80254000 1.76100300 -5.22448700

H 0.60046500 2.61027200 -4.20809400

H 2.09489200 1.98467400 -3.47323700

H 1.33404400 -2.04599200 -4.06104700

H 2.14761900 -0.94643100 -5.21293200

H 2.59072700 -0.92490500 -3.48212800

H -1.15526900 -0.96488900 -5.20923500

H -1.43460200 0.79260700 -5.23514300

H -0.12599200 0.08464200 -6.22831300

O -2.02092100 0.56939300 2.33975000

H -1.65995900 0.26704100 3.19221900

H -2.27362600 2.23777500 2.13344600

O -2.19756600 3.16834000 1.79234000

H -1.45926000 3.21643600 0.81809700

H -3.08982700 3.50860700 1.61992600

O -0.72346000 3.23581000 -0.10500300

H -1.03980200 2.56718900 -0.74960600

H 0.17512200 2.88985600 0.15246800

Electronic Energy (0K) = -1478.4386976

Electronic Energy (0K) + ZPE = -1478.032193

Enthalpy (298K) = -1478.001643

Free Energy (298K) = -1478.094648

**In the presence of TA**

**A*_anti_*_-PMe3_ as the precursor**

**TA**

Number of imaginary frequencies: 0

C -0.41032700 0.98737000 0.01026400

C 0.98895600 1.03188200 -0.08163200

C 1.66580700 2.26134200 -0.13943600

C 0.99450500 3.48975600 -0.10934700

C -0.38204400 3.42559200 -0.01875000

C -1.07714700 2.19474600 0.04007300

H -0.93572800 0.03693100 0.05393200

H 1.52543000 4.43616300 -0.15488400

H -0.95348100 4.35058300 0.00782400

H -2.16206200 2.20894500 0.11035500

N 2.97164800 1.89237900 -0.22049600

H 3.80315100 2.46949800 -0.28214000

N 1.91800500 0.01981200 -0.13162200

N 3.09422400 0.55016900 -0.21468400

Electronic Energy (0K) = -395.6047703

Electronic Energy (0K) + ZPE = -395.498617

Enthalpy (298K) = -395.491788

Free Energy (298K) = -395.528700

**PMe_3_**

Number of imaginary frequencies: 0

P 0.93010300 1.01041400 -0.76192300

C 2.26875800 1.00412800 0.51585400

C -0.50287300 1.43870500 0.32784300

C 0.65280100 -0.81439400 -0.89477500

H -0.42094600 2.48079100 0.65783700

H -1.43992300 1.33424400 -0.23125200

H -0.54463300 0.78952900 1.21301200

H 3.19432400 0.60704200 0.08320300

H 2.46578100 2.02836300 0.85301100

H 1.99349000 0.39201600 1.38542500

H 0.51372500 -1.27427800 0.09304200

H -0.23616800 -1.01238600 -1.50475700

H 1.51137500 -1.28645300 -1.38630000

Electronic Energy (0K) = -460.9658757

Electronic Energy (0K) + ZPE = -460.854017

Enthalpy (298K) = -460.846389

Free Energy (298K) = -460.883270

**[AuPMe_3_]^+^**

Number of imaginary frequencies: 0

Au 2.69883900 4.74844200 -6.00415000

P 3.09302600 3.93355400 -8.09269600

C 3.56836000 2.18520200 -8.08294300

C 1.64098700 4.05264600 -9.16982500

C 4.42760700 4.81458100 -8.94381400

H 1.89464800 3.64400200 -10.15583800

H 0.81321700 3.48207400 -8.73765800

H 1.34247700 5.10051100 -9.27221300

H 4.48439300 2.05382100 -7.49892400

H 2.76724500 1.58745800 -7.63747800

H 3.74038400 1.85812800 -9.11589400

H 4.56158800 4.37974300 -9.94202000

H 4.17030400 5.87415500 -9.03634500

H 5.35628800 4.71721400 -8.37317500

Electronic Energy (0K) = -596.620669

Electronic Energy (0K) + ZPE = -596.506780

Enthalpy (298K) = -596.497380

Free Energy (298K) = -596.541213

**[TAAuPMe_3_]^+^**

Number of imaginary frequencies: 0

C -2.13497900 0.81451000 0.19899800

C -0.87866600 0.46773400 -0.31577500

C -0.18501200 1.47048800 -0.95805900

C -0.71307400 2.77823100 -1.09009700

C -1.94867300 3.12949200 -0.58757200

C -2.65202400 2.10979300 0.06419800

H -0.48488800 -0.53997600 -0.20784800

H 0.79501100 1.25908600 -1.37773700

H -0.12054700 3.52924900 -1.60641700

H -2.35358200 4.13148900 -0.68839800

N -3.07323800 0.08195300 0.88100400

N -3.86463600 2.03699700 0.68273500

H -4.56582800 2.76206400 0.80887400

N -4.10377200 0.82562100 1.16444700

Au -2.98008700 -1.96970600 1.45878900

P -2.87812200 -4.18191500 2.09333000

C -1.74594100 -5.18009500 1.08186400

C -4.47578200 -5.04133800 1.99636600

C -2.31587800 -4.42423100 3.80386000

H -0.73142700 -4.77549600 1.15579700

H -1.75273000 -6.21703300 1.43873800

H -2.06533000 -5.15206800 0.03520800

H -4.35145100 -6.08307200 2.31606800

H -5.20235700 -4.54375900 2.64666600

H -4.84477200 -5.01684900 0.96606000

H -1.31276400 -4.00292000 3.92484300

H -3.00016800 -3.91793500 4.49218700

H -2.29238200 -5.49640100 4.03357500

Electronic Energy (0K) = -992.2764824

Electronic Energy (0K) + ZPE = -992.054291

Enthalpy (298K) = -992.037390

Free Energy (298K) = -992.100415

**TAAuPMe_3_**

Number of imaginary frequencies: 0

C -2.07806200 0.79546200 0.19980900

C -0.89053500 0.46465900 -0.47169200

C -0.25949600 1.48510200 -1.15430500

C -0.78216800 2.80191300 -1.17823300

C -1.94952900 3.12781000 -0.51849300

C -2.60390400 2.09950300 0.18134000

H -0.49118000 -0.54814900 -0.45162500

H 0.66456800 1.27847200 -1.69044300

H -0.24467800 3.56901000 -1.73173800

H -2.35284900 4.13796800 -0.53491100

N -2.94358300 0.06444800 0.95477100

N -3.75552500 2.09960400 0.92066800

N -3.93178500 0.88153100 1.36640700

Au -2.90042100 -1.95809400 1.50145500

P -2.86674200 -4.18183100 2.11580300

C -1.66218300 -5.18683300 1.19578400

C -4.45445100 -5.03538100 1.87439100

C -2.45741400 -4.46384600 3.86540400

H -0.65371600 -4.79199700 1.35638000

H -1.70638200 -6.22682200 1.54119400

H -1.89128400 -5.14779600 0.12589300

H -4.36530600 -6.08247700 2.18831700

H -5.23095000 -4.54139900 2.46741400

H -4.73682100 -4.99388500 0.81739600

H -1.46559200 -4.05381500 4.08198600

H -3.19421300 -3.96170100 4.50071100

H -2.46307100 -5.53960500 4.07914800

Electronic Energy (0K) = -991.8149578

Electronic Energy (0K) + ZPE = -991.606267

Enthalpy (298K) = -991.589524

Free Energy (298K) = -991.652507

**PPh_3_**

Number of imaginary frequencies: 0

P 0.77821300 0.37956400 0.01715500

C 1.67881400 1.20736400 1.39421100

C 1.02381900 1.27223100 2.62968600

C 2.96087300 1.75338200 1.27878200

C 1.64080900 1.85846400 3.73028200

H 0.01971700 0.85813000 2.72871800

C 3.57408600 2.34982200 2.37735900

H 3.48737200 1.71023200 0.32597100

C 2.91710500 2.40169300 3.60399700

H 1.12108800 1.89913400 4.68523700

H 4.57146900 2.77271500 2.27505900

H 3.39828700 2.86859000 4.46076100

C 1.76169500 0.91476700 -1.44609800

C 2.81779200 0.18657600 -2.00208500

C 1.41426200 2.14571400 -2.01569600

C 3.51121700 0.68175100 -3.10393100

H 3.10538800 -0.77161700 -1.57062300

C 2.11401900 2.64543200 -3.10875200

H 0.58661900 2.71864000 -1.59563100

C 3.16285300 1.91094200 -3.65732500

H 4.33076100 0.10553300 -3.52875900

H 1.83553200 3.60539200 -3.53849800

H 3.70683300 2.29508900 -4.51761700

C 1.31202000 -1.37275500 0.21065200

C 0.67662500 -2.31606600 -0.60711900

C 2.28569200 -1.80845500 1.11353800

C 1.01755400 -3.66211200 -0.53615300

H -0.09115500 -1.98865000 -1.30951100

C 2.61743600 -3.15922000 1.19404300

H 2.79172100 -1.09154200 1.75888200

C 1.98776200 -4.08682000 0.36928000

H 0.51952900 -4.38241800 -1.18186600

H 3.37542000 -3.48659200 1.90285200

H 2.24969500 -5.14075100 0.43383700

Electronic Energy (0K) = -1035.7340579

Electronic Energy (0K) + ZPE = -1035.461827

Enthalpy (298K) = -1035.444846

Free Energy (298K) = -1035.508619

**[AuPPh_3_]^+^**

Number of imaginary frequencies: 0

Au 2.61348800 4.20826600 -5.88923800

P 2.95255200 3.65793800 -8.07915000

C 3.21861100 1.87541500 -8.28844700

C 4.09042300 1.22421300 -7.40842300

C 2.61522700 1.16825000 -9.33109600

C 4.35928800 -0.12805100 -7.57603600

H 4.55779800 1.77893000 -6.59430700

C 2.88535600 -0.18848000 -9.48828200

H 1.94001500 1.67107600 -10.02149400

C 3.75436600 -0.83435800 -8.61438700

H 5.03689900 -0.63354600 -6.89229200

H 2.41401900 -0.74005000 -10.29808500

H 3.96060700 -1.89471000 -8.74024000

C 1.52656300 4.11541200 -9.10205000

C 1.69609200 4.58764600 -10.40634000

C 0.24097400 3.93234000 -8.58161100

C 0.57833600 4.87562000 -11.18374000

H 2.69452500 4.72907200 -10.81700000

C -0.86982000 4.21662300 -9.36632500

H 0.11589000 3.56918300 -7.56099800

C -0.70023400 4.69049000 -10.66568900

H 0.70892500 5.24666200 -12.19738600

H -1.86894800 4.07595500 -8.96120000

H -1.57051000 4.92053800 -11.27594700

C 4.40805100 4.49683700 -8.76353600

C 5.22568200 3.86589200 -9.70578600

C 4.66981800 5.81492500 -8.37582500

C 6.30088700 4.55732200 -10.25535600

H 5.02350000 2.84098800 -10.01365200

C 5.74313100 6.49976100 -8.93229300

H 4.03251000 6.29946400 -7.63557700

C 6.55904000 5.86978000 -9.86970000

H 6.93882300 4.06707000 -10.98672700

H 5.94736900 7.52368700 -8.62898200

H 7.40303000 6.40425300 -10.29942200

Electronic Energy (0K) = -1171.3832901

Electronic Energy (0K) + ZPE = -1171.109385

Enthalpy (298K) = -1171.090313

Free Energy (298K) = -1171.161065

**[TAAuPPh_3_]^+^**

Number of imaginary frequencies: 0

C -2.15914600 0.79637200 0.21969300

C -0.89739300 0.44408500 -0.27771700

C -0.19222700 1.44342400 -0.91274400

C -0.71448000 2.75249100 -1.05429000

C -1.95559700 3.10872500 -0.56909000

C -2.67073100 2.09267400 0.07554700

H -0.50857300 -0.56488800 -0.16266200

H 0.79268300 1.22818800 -1.31876300

H -0.11271100 3.50062300 -1.56404900

H -2.35588300 4.11179000 -0.67731700

N -3.10819400 0.06876200 0.89166900

N -3.89144500 2.02460500 0.67887000

H -4.59197200 2.75204000 0.79494800

N -4.14031700 0.81557900 1.16077900

Au -3.00782500 -1.97533000 1.47069200

P -2.86204500 -4.19275000 2.09959400

C -4.48174400 -5.01704200 2.12021200

C -5.60529200 -4.27218700 2.49286200

C -4.61327000 -6.37481500 1.81528300

C -6.85159200 -4.88444500 2.56479900

H -5.50013700 -3.21141800 2.72283900

C -5.86389900 -6.98045000 1.88450900

H -3.74092900 -6.95983900 1.52657000

C -6.98034500 -6.23704400 2.25854100

H -7.72434300 -4.30310300 2.85260300

H -5.96575200 -8.03618900 1.64463400

H -7.95670100 -6.71356700 2.30821600

C -1.81046000 -5.15349100 0.96757600

C -0.93650700 -6.14011900 1.43043900

C -1.92157100 -4.90632400 -0.40524100

C -0.17782200 -6.87268600 0.52172500

H -0.84833300 -6.33983400 2.49749500

C -1.16626900 -5.64565400 -1.30762900

H -2.60108900 -4.13258200 -0.76437100

C -0.29217500 -6.62650600 -0.84337800

H 0.50435500 -7.63826700 0.88371200

H -1.25412400 -5.45115900 -2.37393900

H 0.30437400 -7.19942900 -1.54958800

C -2.14564300 -4.39647300 3.75849500

C -2.62570100 -5.35127000 4.65779700

C -1.05455400 -3.59299200 4.10795000

C -2.01595200 -5.49784800 5.90066800

H -3.47276900 -5.98146300 4.39143100

C -0.44570300 -3.74936600 5.34731300

H -0.68349900 -2.84636900 3.40498100

C -0.92866800 -4.70008400 6.24442500

H -2.39326600 -6.23890700 6.60134400

H 0.40236400 -3.12421800 5.61644500

H -0.45622400 -4.81699400 7.21702900

Electronic Energy (0K) = -1567.0406456

Electronic Energy (0K) + ZPE = -1566.658472

Enthalpy (298K) = -1566.631892

Free Energy (298K) = -1566.720172

**A*_anti_*_-TA_**

Number of imaginary frequencies: 0

C 3.50555000 3.70014700 -2.47439300

O 4.13794800 4.32240300 -1.34967000

C 3.38821500 4.39677800 -0.22102600

O 2.24308900 4.01443700 -0.17938600

C 4.17177900 4.99696200 0.89490300

H 4.50843400 6.00077500 0.61573900

H 5.06602400 4.39575500 1.08886400

H 3.55660700 5.04869900 1.79415600

C 2.42006600 4.56678800 -2.98460200

C 1.44634500 5.26182300 -3.29597100

H 0.51230300 5.79728500 -3.27278800

H 3.04572600 2.74994000 -2.16399600

C 4.54524100 3.47439300 -3.52398200

H 5.15340800 4.34685800 -3.77000700

C 4.65888000 2.31040900 -4.17506800

C 5.53103500 2.03687400 -5.31645900

C 6.25471000 3.04364400 -5.97489400

C 5.60695700 0.72703300 -5.80733400

C 7.03471600 2.74196200 -7.08263100

H 6.19559700 4.07423500 -5.62611000

C 6.38787600 0.42546100 -6.91758100

H 5.04272400 -0.05892500 -5.30660600

C 7.10244000 1.43282600 -7.56004900

H 7.58676100 3.53360700 -7.58526300

H 6.43534500 -0.59752800 -7.28421800

H 7.70848600 1.20153300 -8.43332900

H 4.02559700 1.47789700 -3.85743000

Au 2.59449100 5.31423500 -5.13625500

C 3.43080600 3.12386100 -7.88749800

C 3.69108800 4.49985500 -7.93592800

C 4.35157100 5.08750600 -9.02243900

C 4.79128900 4.33857400 -10.12058000

C 4.53364700 2.98481100 -10.06286100

C 3.86406300 2.38531500 -8.96714500

H 2.91863800 2.67657500 -7.03830100

H 5.30333700 4.79843500 -10.95975300

H 4.85401000 2.35125300 -10.88610100

H 3.69303400 1.31192200 -8.98357000

N 4.41777800 6.40672400 -8.68346900

N 3.42908400 5.50950700 -7.04432600

N 3.87045800 6.64736600 -7.50358200

H 4.81748900 7.18357000 -9.20397100

Electronic Energy (0K) = -1183.8127898

Electronic Energy (0K) + ZPE = -1183.491365

Enthalpy (298K) = -1183.466559

Free Energy (298K) = -1183.548593

**A*_syn_*_-TA_**

Number of imaginary frequencies: 0

C 3.57236200 3.70509200 -2.55368100

O 2.47571900 2.77631500 -2.51940500

C 2.33650400 1.94937000 -3.57963300

O 3.05601300 1.99894100 -4.55359200

C 1.19359300 1.01578100 -3.37705100

H 0.25743700 1.58535100 -3.38607900

H 1.26860200 0.52402800 -2.40270700

H 1.17460300 0.27324300 -4.17610000

C 3.16865700 4.92748500 -3.27754000

C 2.88656100 6.07158600 -3.65397300

H 2.67994400 7.12634800 -3.71767000

H 4.42535300 3.24674400 -3.07156600

C 3.92674800 4.06098500 -1.14709000

H 3.11401300 4.46478400 -0.54239300

C 5.17265600 3.91004900 -0.68196800

C 5.65741000 4.23486400 0.65840200

C 4.84587900 4.81695300 1.64384400

C 6.99293600 3.95048700 0.97235300

C 5.35852100 5.09913900 2.90194700

H 3.80640300 5.05366500 1.42489300

C 7.50620100 4.23401500 2.23281900

H 7.63027100 3.50042100 0.21196100

C 6.68959600 4.80887500 3.20190400

H 4.71731900 5.55064500 3.65586400

H 8.54563600 4.00606500 2.45811200

H 7.08717900 5.03301000 4.18924500

H 5.92414200 3.49605500 -1.35933300

Au 2.69067400 4.87407800 -5.45658000

C 4.47641400 4.84355400 -8.64870600

C 3.19901200 4.29022500 -8.49612400

C 2.53033700 3.67592000 -9.56290300

C 3.09455500 3.57853100 -10.83990100

C 4.35244300 4.12638000 -10.98089600

C 5.03344800 4.74848700 -9.90564700

H 4.98739700 5.31861500 -7.81490900

H 2.57213500 3.10118300 -11.66271800

H 4.84228000 4.08105100 -11.95014300

H 6.02325800 5.16033100 -10.08395500

N 1.34705600 3.27745200 -9.01454000

N 2.36384500 4.20775600 -7.41100900

N 1.25699500 3.59839300 -7.73543600

H 0.56853500 2.79072200 -9.45218600

Electronic Energy (0K) = -1183.8103069

Electronic Energy (0K) + ZPE = -1183.488540

Enthalpy (298K) = -1183.463595

Free Energy (298K) = -1183.548008

**TA-H_2_O**

Number of imaginary frequencies: 0

C 3.31953200 2.92781100 -3.91369800

C 4.71833600 3.00330100 -3.83706100

C 5.51201500 1.88334500 -3.56080800

C 4.83390900 0.69570900 -3.36738000

C 3.42309400 0.60860100 -3.44286600

C 2.64489000 1.71406100 -3.71521700

H 6.59467000 1.94536900 -3.50213100

H 5.40158100 -0.20606400 -3.14939100

H 2.94933400 -0.35669600 -3.28132800

H 1.55969100 1.67878800 -3.78152500

H 5.86056800 4.80511100 -4.12509300

N 3.83467300 5.00033500 -4.29470700

N 2.83720800 4.18274200 -4.19627800

N 4.97198500 4.31723600 -4.08319600

O -0.01194600 3.62740300 -4.14140800

H 0.85687800 4.04584700 -4.26498600

H -0.16195400 3.70213200 -3.19320700

Electronic Energy (0K) = -472.0070954

Electronic Energy (0K) + ZPE = -471.876213

Enthalpy (298K) = -471.866045

Free Energy (298K) = -471.911156

**TS_C-1W-TA_**

Number of imaginary frequencies: 1

C 3.27779600 2.76173400 -3.77286100

C 4.63142600 3.09426400 -3.92746600

C 5.64167700 2.12591000 -3.97056000

C 5.22258900 0.81514900 -3.85685700

C 3.85807200 0.46877700 -3.69666600

C 2.86720600 1.42635600 -3.64885400

H 6.68851200 2.39347600 -4.08064300

H 5.96362600 0.01885300 -3.88310500

H 3.59299700 -0.58178700 -3.60739000

H 1.81728700 1.17024100 -3.53218400

H 5.39116400 5.10028800 -4.12963300

N 3.37760800 4.93561400 -3.94516100

N 2.56298000 3.93339600 -3.79828100

N 4.61482000 4.45361800 -4.02342400

O 0.09403400 4.52902700 -4.40783900

H 1.05783200 4.25599100 -4.12250300

H -0.52956800 3.97499000 -3.90539100

C 1.84965800 4.02942500 -6.96005200

O 0.77852500 4.97554700 -6.67169300

C -0.22359300 4.32023500 -6.09554100

O -0.14421000 3.00400900 -6.29073800

C -1.56956000 4.93434500 -6.20538100

H -1.52993300 5.98092000 -5.89762900

H -2.29441500 4.39076300 -5.59442200

H -1.87781100 4.87636100 -7.25389200

C 1.17857900 2.69436700 -6.72052400

C 1.63282400 1.45748300 -6.85632200

H 0.92595000 0.66778300 -6.58744800

Au 3.57825200 1.04569100 -7.46290000

P 5.77674100 0.53764300 -8.16515200

H 2.65563900 4.21213800 -6.23698900

C 2.37182100 4.18793600 -8.33873500

H 1.64801000 4.09119400 -9.14994000

C 3.68982800 4.27609700 -8.56033300

C 4.36155700 4.19387700 -9.85692500

C 3.73946300 3.63408400 -10.98307500

C 5.68365500 4.64447600 -9.97457500

C 4.41330800 3.55552600 -12.19501600

H 2.72759100 3.23957600 -10.90169100

C 6.35654300 4.56734800 -11.18931000

H 6.17880600 5.06847000 -9.10129700

C 5.72191100 4.02464900 -12.30393900

H 3.91926100 3.11589000 -13.05879100

H 7.37960400 4.92912900 -11.26519200

H 6.24833500 3.95761300 -13.25336800

H 4.34812100 4.39152200 -7.69230900

C 6.11008900 0.81809700 -9.93653600

C 6.25089500 -1.20071400 -7.88764300

C 7.10525900 1.47633800 -7.33945700

H 6.10993900 1.89688300 -10.13513200

H 7.09124300 0.40471300 -10.20111100

H 5.33535600 0.34437100 -10.54853300

H 6.15144500 -1.43764900 -6.82222700

H 5.58558400 -1.85898200 -8.45636300

H 7.28762600 -1.36903400 -8.20382000

H 6.91209400 2.55197300 -7.42754600

H 7.13710300 1.20826700 -6.27786500

H 8.07304200 1.24192500 -7.79999400

Electronic Energy (0K) = -1721.2268248

Electronic Energy (0K) + ZPE = -1720.763326

Enthalpy (298K) = -1720.730856

Free Energy (298K) = -1720.829047

**C-1W-TA**

Number of imaginary frequencies: 0

C -0.23017300 0.15949700 3.35373300

C 1.09419700 0.61607600 3.29617900

C 2.18553700 -0.25907400 3.30193100

C 1.87010300 -1.60021000 3.35772500

C 0.53154400 -2.06299800 3.41742600

C -0.54488800 -1.20226700 3.42027200

H 3.20829200 0.10199700 3.26585500

H 2.67438100 -2.33174500 3.36339200

H 0.35273700 -3.13394300 3.46319400

H -1.57270400 -1.54802900 3.46159400

H 1.71484700 2.67728300 3.16937600

N -0.26534800 2.38149400 3.21793300

N -0.98724000 1.29741800 3.30065800

N 0.97659800 1.97662000 3.22203800

O -3.44811700 2.30189900 2.45856700

H -2.01092300 1.42271100 3.19032800

H -4.22346200 2.08907700 3.00042600

C -1.77350300 1.79349200 0.11580700

O -2.83583300 2.72805500 0.32449900

C -3.80092100 2.10687700 1.09274100

O -3.73474800 0.73683600 0.79455900

C -5.16920900 2.64883500 0.80096800

H -5.19967400 3.71658900 1.03523100

H -5.92121900 2.12771500 1.40232300

H -5.40098600 2.50326300 -0.25714900

C -2.44031900 0.45273700 0.38718900

C -1.94049100 -0.77831900 0.27112300

H -2.63020700 -1.57769100 0.56155600

Au 0.00339700 -1.19216800 -0.31822800

P 2.21302400 -1.71295100 -0.99166800

H -0.96257900 1.98364100 0.83871700

C -1.21858600 1.89285200 -1.25865400

H -1.93443800 1.77937200 -2.07552700

C 0.09943500 1.98112300 -1.47744600

C 0.77534000 1.87229600 -2.77091300

C 0.16369400 1.27132900 -3.88161300

C 2.08939000 2.34226300 -2.90367600

C 0.83783500 1.17167300 -5.09207300

H -0.84154000 0.86246400 -3.78824000

C 2.76326900 2.24313500 -4.11651900

H 2.57769100 2.79977500 -2.04336300

C 2.13813100 1.65964000 -5.21587100

H 0.35021700 0.70038400 -5.94285900

H 3.77991600 2.62064100 -4.20300900

H 2.66498800 1.57597700 -6.16378200

H 0.75467600 2.12518500 -0.61124000

C 2.56904700 -1.45698100 -2.76322000

C 2.69147400 -3.44787300 -0.69416300

C 3.53319300 -0.76026000 -0.16574600

H 2.55683800 -0.38203500 -2.98161900

H 3.55832400 -1.86249500 -3.00913800

H 1.80789500 -1.95095500 -3.37650600

H 2.58150400 -3.68104800 0.37100400

H 2.03447100 -4.11197400 -1.26598300

H 3.73189700 -3.61552500 -0.99845100

H 3.35117300 0.31295700 -0.29770800

H 3.53796000 -0.98801700 0.90630900

H 4.50996700 -1.01729800 -0.59420600

Electronic Energy (0K) = -1721.2435619

Electronic Energy (0K) + ZPE = -1720.776045

Enthalpy (298K) = -1720.743558

Free Energy (298K) = -1720.840974

**TA-(H_2_O)_2_**

Number of imaginary frequencies: 0

C 2.34145300 -0.04385400 -1.84473500

C 1.03092900 -0.45410600 -1.55600300

C 0.72633900 -1.25773800 -0.45091600

C 1.79705900 -1.62410400 0.34049500

C 3.12217100 -1.21567000 0.05635300

C 3.42102400 -0.42340500 -1.03300700

H -0.28942100 -1.57278100 -0.23102800

H 1.61858900 -2.24845300 1.21304500

H 3.92086900 -1.54002900 0.71928900

H 4.43974300 -0.10493100 -1.26512400

H -0.69808900 0.06268500 -2.73081700

N 1.08059800 0.80900400 -3.39381000

N 2.30424000 0.72663700 -2.98095500

N 0.30018000 0.10989900 -2.55589800

O 6.46406500 0.52389300 -2.04068900

H 5.92426600 1.03534800 -2.67693200

H 6.61525600 -0.31297500 -2.49167400

O 4.78588100 1.89970000 -3.79691600

H 3.89652600 1.52365500 -3.63506600

H 4.71946900 2.81010000 -3.48767500

Electronic Energy (0K) = -548.4136351

Electronic Energy (0K) + ZPE = -548.257322

Enthalpy (298K) = -548.244376

Free Energy (298K) = -548.296844

**TS_C-2W-TA_**

Number of imaginary frequencies: 1

C 6.28002700 3.85402200 -0.56180700

O 6.67364600 3.39474400 0.75398200

C 6.17200100 2.14706000 0.92210900

O 5.06712600 1.98146600 0.14008100

C 5.98061100 1.76000800 2.34784900

H 5.24286200 2.43088500 2.79607800

H 6.92715700 1.84911300 2.88708500

H 5.62312000 0.72935400 2.40978100

C 5.10273100 2.95569600 -0.88473000

C 4.24561900 2.98388000 -1.89815100

H 3.50958600 2.17419600 -1.89975400

Au 4.35387600 4.38981600 -3.42189100

P 4.49100500 5.99502000 -5.15125700

C 5.94163200 5.29811400 -0.53954500

H 5.17227100 5.59861100 0.17456400

C 6.45976400 6.15423200 -1.42899600

C 6.06036200 7.54894800 -1.62138400

C 4.82607600 8.04141100 -1.17109500

C 6.91834900 8.41916700 -2.30797400

C 4.47724000 9.37009300 -1.38007000

H 4.12727500 7.37232100 -0.67071800

C 6.56667100 9.74774700 -2.52031700

H 7.87544600 8.04250400 -2.66890500

C 5.34606700 10.22836500 -2.05324500

H 3.51622100 9.73669900 -1.02566400

H 7.24765700 10.40959000 -3.05091400

H 5.06716700 11.26634400 -2.22021800

H 7.24466100 5.78822100 -2.09966000

O 7.22230800 1.15659700 0.35653600

H 6.86443000 0.71705500 -0.46344400

C 7.09150500 1.80387000 -4.10762800

C 5.95200800 1.00855100 -4.32116200

C 5.21635800 1.05129300 -5.51239300

C 5.67844100 1.93021400 -6.47039000

C 6.82213500 2.74248800 -6.26542200

C 7.54466600 2.69743400 -5.09214400

H 4.34101800 0.42862700 -5.66987700

H 5.15162800 2.00209400 -7.41916200

H 7.13471300 3.41270900 -7.06318200

H 8.42429100 3.31472700 -4.92761300

H 8.13492200 1.63409700 0.02392800

H 5.11745400 -0.35826500 -2.88144500

N 6.78912300 0.64919500 -2.30310900

N 7.56386700 1.53524900 -2.85198000

N 5.82484500 0.31553500 -3.16014400

O 9.19051100 2.17451700 -0.69283200

H 10.03951600 1.72377500 -0.57506200

H 8.90540300 2.03456600 -1.62786100

H 7.11333300 3.67747000 -1.26281600

C 3.83499200 5.45716200 -6.76590100

C 6.18982400 6.53834600 -5.53592500

C 3.61567200 7.56118800 -4.82297100

H 4.38224100 4.57040500 -7.10621900

H 2.77607200 5.19671600 -6.66420800

H 3.94362700 6.25861900 -7.50697500

H 4.01959900 8.02507800 -3.91416900

H 3.73993900 8.24860400 -5.66885400

H 2.54952100 7.36269600 -4.66979200

H 6.62825600 7.01567900 -4.65119000

H 6.80430100 5.67384400 -5.81102100

H 6.17977200 7.25732800 -6.36456900

Electronic Energy (0K) = -1797.6459398

Electronic Energy (0K) + ZPE = -1797.157747

Enthalpy (298K) = -1797.123197

Free Energy (298K) = -1797.225416

**C-2W-TA**

Number of imaginary frequencies: 0

C 1.01437700 -0.47302100 2.63846900

O 1.70302600 -0.88854800 3.83577700

C 1.27312900 -2.14827100 4.14723300

O 0.00305400 -2.32373200 3.64431000

C 1.37894000 -2.45074300 5.60528500

H 0.74848600 -1.75251400 6.16206100

H 2.41729600 -2.34136500 5.92959100

H 1.04935100 -3.47516800 5.79646000

C -0.19818000 -1.38390700 2.61724900

C -1.26291900 -1.37589500 1.82135400

H -1.97848800 -2.18359300 2.00395300

Au -1.49956800 -0.00562700 0.28091200

P -1.75457000 1.56536100 -1.46800400

C 0.67671000 0.97116000 2.70004300

H 0.08928000 1.28680300 3.56459100

C 0.97699800 1.81075500 1.70147100

C 0.53972500 3.20230000 1.57973700

C -0.56228300 3.70490200 2.28809700

C 1.21761700 4.05878100 0.70124100

C -0.95356400 5.02987700 2.13835100

H -1.12856900 3.04651500 2.94573000

C 0.82260500 5.38335600 0.54845700

H 2.06996400 3.67426700 0.14098800

C -0.26256200 5.87423200 1.26980500

H -1.81121200 5.40389500 2.69349400

H 1.36380200 6.03410700 -0.13490700

H -0.57593700 6.90890400 1.15011000

H 1.59233000 1.43295200 0.87761200

O 2.15138900 -3.12002200 3.42490800

H 1.60035600 -3.65118200 2.80265000

C 1.04036400 -2.62117100 -0.94321700

C -0.11237200 -3.42378400 -0.90743500

C -1.08652800 -3.38687900 -1.91297100

C -0.84597400 -2.50653400 -2.94770500

C 0.30975600 -1.68706700 -2.99202200

C 1.26656500 -1.72433600 -1.99991000

H -1.97107000 -4.01541100 -1.87943300

H -1.56346300 -2.44018300 -3.76237600

H 0.43964800 -1.01527100 -3.83766600

H 2.15566600 -1.09932100 -2.02639100

H 3.02478700 -2.62999400 2.72365700

H -0.60881700 -4.80000600 0.67162700

N 1.13744200 -3.78274400 0.88798100

N 1.76890600 -2.89097000 0.18348800

N 0.01750300 -4.11791200 0.25402200

O 3.72806900 -2.21745200 1.86226300

H 4.58115200 -2.67857000 1.80578800

H 3.19276400 -2.39824300 1.02738400

H 1.65844200 -0.66241700 1.76216600

C -2.76294400 1.00229700 -2.87946300

C -0.19343300 2.10273900 -2.24518300

C -2.53681900 3.13764800 -0.97399600

H -2.30006100 0.11583100 -3.32774200

H -3.76662100 0.73510900 -2.53217900

H -2.83752300 1.79458200 -3.63451400

H -1.94045100 3.61369400 -0.18523200

H -2.60734700 3.81312500 -1.83554800

H -3.54083800 2.94218900 -0.58229600

H 0.43850800 2.59105200 -1.49370500

H 0.34202400 1.23555200 -2.64766200

H -0.40000500 2.81170800 -3.05657100

Electronic Energy (0K) = -1797.6463631

Electronic Energy (0K) + ZPE = -1797.157973

Enthalpy (298K) = -1797.123021

Free Energy (298K) = -1797.226681

**TA-(H_2_O)_3_**

Number of imaginary frequencies: 0

C 3.61158900 0.12154800 -4.30129000

C 2.46699200 -0.69697900 -4.30685000

C 2.54564600 -2.09454500 -4.24810900

C 3.81695200 -2.62674600 -4.18377400

C 4.97667300 -1.81406800 -4.17796500

C 4.89710300 -0.43926000 -4.23637300

H 1.65751500 -2.71952600 -4.25323000

H 3.93652500 -3.70664300 -4.13611100

H 5.95083700 -2.29406500 -4.12607700

H 5.78187800 0.19168000 -4.23256200

H 0.40950400 0.08882600 -4.33650900

N 1.91034500 1.43808300 -4.40608500

N 3.20431300 1.42989600 -4.36299300

N 1.43930000 0.18744900 -4.37713100

O -0.07448600 3.36663600 -3.98285800

H 0.66486500 2.84135900 -4.35075300

H -0.33955600 3.96933200 -4.68907600

O 2.79998100 4.33636800 -3.59443300

H 3.17106400 3.46402400 -3.78925400

H 1.87490700 4.15497000 -3.38823000

O -1.24372400 0.80280100 -4.10041900

H -1.04508300 1.75621400 -4.01113200

H -1.59102800 0.54348700 -3.23883300

Electronic Energy (0K) = -624.8209745

Electronic Energy (0K) + ZPE = -624.639545

Enthalpy (298K) = -624.623754

Free Energy (298K) = -624.683219

**B-3W-TA**

Number of imaginary frequencies: 0

C 0.74303300 -1.07040000 2.84169900

C -0.27625800 -2.03659000 2.77296500

C -0.01544400 -3.38047700 2.47611200

C 1.30927600 -3.70757600 2.26129900

C 2.34524400 -2.74563400 2.33799300

C 2.08420600 -1.42114800 2.62579800

H -0.80800400 -4.12156400 2.42588000

H 1.56984600 -4.73862700 2.03243700

H 3.37115400 -3.06602400 2.16787000

H 2.87461700 -0.67700300 2.69134100

H -2.39705300 -1.57445400 3.03018000

N -1.11864600 -0.03809500 3.23823900

N 0.16166000 0.13947800 3.13154600

N -1.40369400 -1.32674500 3.03962400

O -3.21006600 2.17459100 3.05390200

H -2.26930200 2.41360400 3.17283800

H -3.25225800 1.22317200 3.24233300

O -0.45885700 2.89838300 3.13095200

H 0.02051200 2.05640200 3.25332300

H -0.16886500 3.47510700 3.84672600

C -1.57847000 2.00330500 0.22734800

O -2.70299400 2.95167500 0.43589700

C -3.75065200 2.27553800 0.74841100

O -3.66299500 0.99923800 0.57978700

C -5.03627200 2.91997000 1.01521800

H -4.87712600 3.88338000 1.50075800

H -5.65275100 2.26961900 1.63866600

H -5.54335200 3.07931800 0.05510900

C -2.30163200 0.68152400 0.18895500

C -1.91695000 -0.54914100 -0.09397700

H -2.68618100 -1.32025600 0.00350800

Au 0.02992100 -0.98952500 -0.68402300

P 2.19161400 -1.47639900 -1.50450000

H -0.95602700 2.11424400 1.12597000

C -0.82010300 2.33528300 -0.99810800

H -1.38581100 2.34699700 -1.93102200

C 0.51183600 2.47604500 -0.95761000

C 1.41146900 2.63485600 -2.09847300

C 0.97551000 2.53436500 -3.42867200

C 2.77128900 2.87014700 -1.85455100

C 1.87433000 2.67827700 -4.47717000

H -0.07267500 2.33474200 -3.64490500

C 3.67252000 3.00535800 -2.90509500

H 3.11655700 2.95016100 -0.82389500

C 3.22557300 2.91218700 -4.21994000

H 1.52168300 2.59943800 -5.50317400

H 4.72439700 3.18698200 -2.69623000

H 3.92695600 3.01783000 -5.04450000

H 0.99917500 2.44150700 0.02262300

C 2.39332500 -0.99726000 -3.25246300

C 2.67012900 -3.23581400 -1.48701400

C 3.57724300 -0.63727900 -0.66712800

H 2.21706600 0.07958400 -3.36400800

H 3.40642200 -1.23973800 -3.59656200

H 1.66199300 -1.53549500 -3.86519400

H 2.66709800 -3.61236400 -0.45799600

H 1.95048200 -3.81559000 -2.07487000

H 3.67240800 -3.35747800 -1.91598400

H 3.38226900 0.44048000 -0.61943800

H 3.68048300 -1.02296700 0.35276000

H 4.50961900 -0.81432600 -1.21763800

O -4.08585600 -0.64861800 2.98724400

H -4.46952700 -0.56419200 2.10459900

H -4.81832000 -0.91264400 3.55908300

Electronic Energy (0K) = -1874.0571335

Electronic Energy (0K) + ZPE = -1873.543632

Enthalpy (298K) = -1873.503476

Free Energy (298K) = -1873.617071

**TS_C-3W-TA_**

Number of imaginary frequencies: 1

C 3.62209800 0.41472000 -4.40540800

C 2.65915300 -0.60499200 -4.50735800

C 2.99668900 -1.92457900 -4.83334600

C 4.33934000 -2.17146300 -5.04316200

C 5.31903800 -1.15456600 -4.93759500

C 4.98241800 0.14599500 -4.62178700

H 2.24730500 -2.70719800 -4.90820400

H 4.65902000 -3.18093400 -5.29238800

H 6.36219800 -1.41335400 -5.10723700

H 5.72909600 0.93176300 -4.53425200

H 0.52013100 -0.27531700 -4.25622800

N 1.70225600 1.33095600 -4.00035400

N 2.97171000 1.58147300 -4.08927700

N 1.49165400 0.03484800 -4.23485000

O -0.29887100 3.50568300 -4.42239700

H 0.63203900 3.77443600 -4.17805200

H -0.40386000 2.54897000 -4.21528800

O 2.21280300 4.27426700 -3.85894100

H 2.74841800 3.45657700 -3.80810800

H 2.31627900 4.71449100 -3.00646700

C 1.54705100 3.41133000 -6.94073500

O 0.48752600 4.37207800 -6.61991200

C -0.57613600 3.69655400 -6.24427900

O -0.55968600 2.43782700 -6.65085300

C -1.87687800 4.39367100 -6.19820100

H -1.77265800 5.34759000 -5.67883100

H -2.61628300 3.77004400 -5.69101200

H -2.20121600 4.57274400 -7.22903700

C 0.77724200 2.11454600 -7.05080300

C 1.14380000 0.89597800 -7.41394400

H 0.35741800 0.13717800 -7.35733800

Au 3.07925600 0.44819300 -8.02812500

P 5.24584700 -0.04433100 -8.83737200

H 2.23826800 3.40127700 -6.08494000

C 2.25782900 3.80125200 -8.18073900

H 1.63920000 3.93650400 -9.06979300

C 3.59535100 3.84898500 -8.22585700

C 4.41742100 4.03355900 -9.42153400

C 3.90486500 3.87303200 -10.71787100

C 5.77445000 4.35012200 -9.27171200

C 4.72309600 4.04918900 -11.82662600

H 2.86220300 3.59100000 -10.85753300

C 6.59429200 4.52051900 -10.38204700

H 6.18146400 4.46859700 -8.26756300

C 6.06953900 4.37432100 -11.66337200

H 4.31163900 3.92117700 -12.82549800

H 7.64458200 4.76889600 -10.24632300

H 6.70849300 4.50470100 -12.53385600

H 4.14678600 3.71640600 -7.28900300

C 5.46695200 0.37139200 -10.59944000

C 5.74365600 -1.79648700 -8.74808700

C 6.61527600 0.84234400 -8.02210200

H 5.29731300 1.44480900 -10.74838300

H 6.48283000 0.11424100 -10.92415600

H 4.74036100 -0.18403800 -11.20217900

H 5.74044600 -2.13122400 -7.70489400

H 5.03236600 -2.40736800 -9.31425900

H 6.74878900 -1.92483000 -9.16831300

H 6.44665000 1.92328900 -8.09844300

H 6.66202700 0.56588000 -6.96315900

H 7.56684800 0.59027100 -8.50652200

O -1.20371400 0.84604600 -4.32951100

H -1.56462300 0.84298900 -5.22689100

H -1.95070500 0.64149200 -3.75119400

Electronic Energy (0K) = -1874.054022

Electronic Energy (0K) + ZPE = -1873.540069

Enthalpy (298K) = -1873.501431

Free Energy (298K) = -1873.612302

**C-3W-TA**

Number of imaginary frequencies: 0

C 0.34586900 -0.85747500 3.00623000

C -0.48759800 -1.95378800 2.72157900

C 0.01599500 -3.18656100 2.28924100

C 1.38868000 -3.26633100 2.16176600

C 2.23814900 -2.16942100 2.44562000

C 1.73892400 -0.95382900 2.86724300

H -0.63498500 -4.02892800 2.07505900

H 1.83631400 -4.20316300 1.83758000

H 3.31244800 -2.29771400 2.33076700

H 2.38528400 -0.10895800 3.09150300

H -2.65840500 -1.88749700 2.82211100

N -1.69533200 -0.20493000 3.35063500

N -0.45827200 0.18627700 3.39048000

N -1.73824500 -1.47487500 2.96396100

O -3.49034300 1.98510400 2.61069500

H -2.47085400 2.31872200 3.14416000

H -3.75298200 1.03649000 2.76990400

O -1.43589000 2.64804400 3.67004000

H -0.82764800 1.85051200 3.69444800

H -1.56168100 2.97161300 4.57628000

C -1.50190800 2.14164300 0.44083600

O -2.55438300 3.05716700 0.81247700

C -3.64081500 2.31115400 1.17739700

O -3.59868400 1.11755900 0.47661800

C -4.93804300 3.02771600 0.99396000

H -4.93420700 3.95043800 1.57980400

H -5.75877600 2.39002700 1.33667100

H -5.07677900 3.26461000 -0.06404600

C -2.25293600 0.84955400 0.17308500

C -1.81493600 -0.32828400 -0.25989800

H -2.58162200 -1.10791400 -0.32473900

Au 0.16189600 -0.68021700 -0.78507700

P 2.39868500 -1.04383700 -1.46754100

H -0.80790100 2.01726900 1.28906000

C -0.75802400 2.64356800 -0.74189800

H -1.35986900 2.86434600 -1.62601400

C 0.57927000 2.70305300 -0.76273600

C 1.41230200 2.99499600 -1.93015100

C 0.93290100 2.85639200 -3.24156500

C 2.74394700 3.39008800 -1.74257400

C 1.75496500 3.13237300 -4.32736700

H -0.08626200 2.51054500 -3.40975800

C 3.56758200 3.66150400 -2.82986500

H 3.12739400 3.49124300 -0.72720900

C 3.07413100 3.53719400 -4.12613500

H 1.36849500 3.01934000 -5.33805100

H 4.59723300 3.97123300 -2.66465600

H 3.71691200 3.74631800 -4.97831600

H 1.12026800 2.49579400 0.16672900

C 2.72668600 -0.56083500 -3.19614800

C 2.98962400 -2.76760700 -1.38453500

C 3.65596100 -0.11031100 -0.53095500

H 2.49930700 0.50426100 -3.32918500

H 3.77868200 -0.74341400 -3.44822300

H 2.08564000 -1.14125400 -3.86840000

H 2.94362500 -3.12445800 -0.34941200

H 2.34893900 -3.40486000 -2.00348800

H 4.02369400 -2.83148600 -1.74502500

H 3.45728400 0.96407500 -0.62656800

H 3.61367500 -0.38453700 0.52890500

H 4.65722200 -0.32707300 -0.92368200

O -4.44066700 -0.57085300 2.54913600

H -4.57047700 -0.52355900 1.58985300

H -5.31055600 -0.76994800 2.92137200

Electronic Energy (0K) = -1874.0574831

Electronic Energy (0K) + ZPE = -1873.543859

Enthalpy (298K) = -1873.506131

Free Energy (298K) = -1873.615259

**TA-(H_2_O)_4_**

Number of imaginary frequencies: 0

O 6.23342600 3.93903700 -1.73924700

H 5.56510500 4.14764600 -1.07746200

C 2.52444800 0.04041400 -1.59373200

C 1.16573900 -0.18780600 -1.32604300

C 0.72965300 -1.24871300 -0.52326100

C 1.72003200 -2.06162200 -0.00832100

C 3.09280800 -1.83954700 -0.27153400

C 3.52023600 -0.79309800 -1.06255400

H -0.32306000 -1.42105300 -0.31936100

H 1.43834400 -2.90202200 0.62199500

H 3.82379000 -2.51597200 0.16509000

H 4.57562900 -0.61025200 -1.26851400

H 6.89793900 3.39707900 -1.25745900

H -0.44787400 1.02019100 -2.08331300

N 1.42878700 1.59389100 -2.64173400

N 2.61897700 1.14551200 -2.40405000

N 0.54208600 0.81207800 -2.00626800

O 7.96285200 2.18813700 -0.51187800

H 7.74207700 2.09150500 0.42104200

H 7.60192000 1.37832400 -0.93747800

O 6.69192900 0.14500300 -1.80672900

H 6.17256300 0.71900600 -2.42674000

H 7.21754900 -0.43214100 -2.37174900

O 5.28091200 1.86477600 -3.31324900

H 4.34328200 1.72245200 -3.08466500

H 5.54673800 2.67739100 -2.83291600

Electronic Energy (0K) = -701.232116

Electronic Energy (0K) + ZPE = -701.023850

Enthalpy (298K) = -701.006055

Free Energy (298K) = -701.071857

**TS_C-4W-TA_**

Number of imaginary frequencies: 1

C 1.14175200 0.31437200 2.73054500

C 0.91172100 -1.07109000 2.66247700

C 1.92984800 -1.98567900 2.36036100

C 3.17705800 -1.44449000 2.12602200

C 3.41855000 -0.04836200 2.18176200

C 2.41677900 0.84898800 2.48352800

H 1.74235800 -3.05535600 2.31758000

H 4.00581100 -2.10745400 1.88543000

H 4.42379900 0.31604100 1.98300000

H 2.59570600 1.91987400 2.54080900

H -1.06290100 -1.99954000 2.88620900

N -0.95206500 0.00409500 3.15181900

N -0.04416900 0.92454500 3.04079800

N -0.40948400 -1.19623700 2.94315700

O -4.01207100 2.35758600 2.19435700

H -3.15858500 2.72680900 2.56191800

H -4.07758900 1.43070000 2.53783100

O -1.70776900 3.20635400 3.21209500

H -0.99158800 2.53776800 3.24509700

H -1.78447800 3.55956800 4.10648600

C -1.77097800 1.83782100 0.12424700

O -2.81908000 2.85995000 0.06676700

C -3.96290700 2.27013700 0.34710800

O -3.93560800 0.96387300 0.15707400

C -5.21390400 2.99613700 0.04889000

H -5.15462000 4.01715300 0.42973100

H -6.06391600 2.47803300 0.49637000

H -5.33819000 3.02153300 -1.03946200

C -2.57031600 0.56387200 0.00119500

C -2.21197500 -0.69295400 -0.21152200

H -3.03793700 -1.40961000 -0.21044500

Au -0.29185200 -1.31542700 -0.70455100

P 1.73560300 -2.17832800 -1.57973100

H -1.30118600 1.92167300 1.11603000

C -0.75798300 2.03960300 -0.93662500

H -1.11998400 2.00386600 -1.96559500

C 0.54272400 2.14212900 -0.63328500

C 1.65981200 2.17725600 -1.57521600

C 1.51602700 1.83279000 -2.92820900

C 2.92974500 2.53896800 -1.10657400

C 2.60803600 1.87303700 -3.78575300

H 0.54543700 1.51689900 -3.30870200

C 4.02228000 2.57992900 -1.96577000

H 3.05145900 2.79561900 -0.05438900

C 3.86404600 2.24973500 -3.30902800

H 2.48176200 1.60149200 -4.83176900

H 4.99962400 2.86890100 -1.58559700

H 4.71728600 2.27708500 -3.98308800

H 0.81777000 2.18394500 0.42568700

C 1.54982600 -2.52557900 -3.36221300

C 2.26005100 -3.78605200 -0.89461700

C 3.24640800 -1.15808100 -1.53189200

H 1.32063600 -1.59311500 -3.89094900

H 2.47608300 -2.95331000 -3.76568100

H 0.72538600 -3.22981800 -3.51765800

H 2.54886600 -3.67034500 0.15527800

H 1.42989000 -4.49806500 -0.95498500

H 3.11415600 -4.17369700 -1.46363600

H 3.11219500 -0.29520000 -2.19454300

H 3.44239100 -0.79986800 -0.51565300

H 4.10031900 -1.75074600 -1.88321100

O -3.74175700 -0.13667000 3.20411900

H -2.78047500 0.01108400 3.35708000

H -4.13959500 -0.22398900 4.08186300

O -2.66465900 -2.70829700 2.55239000

H -3.01450400 -3.32582100 3.20628400

H -3.24709300 -1.92807900 2.60091000

Electronic Energy (0K) = -1950.467811

Electronic Energy (0K) + ZPE = -1949.926456

Enthalpy (298K) = -1949.886158

Free Energy (298K) = -1949.999097

**C-4W-TA**

Number of imaginary frequencies: 0

C 0.99988500 0.46769900 2.86529400

C 0.85059300 -0.91598000 2.66932700

C 1.91218300 -1.73606000 2.26417200

C 3.11833700 -1.09989400 2.05625000

C 3.27635900 0.29802400 2.23463900

C 2.23374200 1.10179200 2.64376600

H 1.78474000 -2.80651400 2.12627900

H 3.98009000 -1.68570400 1.74256500

H 4.25250300 0.74000600 2.04909000

H 2.35086500 2.17239200 2.79101400

H -1.06089600 -1.98251400 2.81863400

N -1.07211800 -0.01596600 3.27111700

N -0.21919900 0.96496000 3.24238400

N -0.45726900 -1.14847400 2.94806100

O -3.65836400 2.29008400 2.06736100

H -2.70815400 2.74079400 2.65752800

H -3.88179000 1.35550400 2.34906100

O -1.75643000 3.14261200 3.25518200

H -1.05760800 2.41833100 3.35332300

H -1.97882200 3.49222300 4.13218200

C -1.50124400 1.90476600 0.13000300

O -2.46675900 2.97154400 0.22196400

C -3.65613700 2.40165300 0.59385100

O -3.69548700 1.11353400 0.10215800

C -4.83726500 3.21679600 0.17959900

H -4.76994900 4.21301900 0.62495700

H -5.75773100 2.73400500 0.51852400

H -4.85058200 3.30508600 -0.90994100

C -2.37307200 0.67856200 -0.05916500

C -2.04029900 -0.58136600 -0.32567500

H -2.88921000 -1.27076100 -0.37008700

Au -0.13711200 -1.24195700 -0.81313200

P 1.90248400 -2.11482600 -1.65221700

H -0.94655500 1.83545900 1.08006400

C -0.53711500 2.14862100 -0.97182800

H -0.96123100 2.26567400 -1.97125300

C 0.78384500 2.13542000 -0.75264900

C 1.84069700 2.22622600 -1.75868000

C 1.61063800 1.95677300 -3.11592000

C 3.13833200 2.56283200 -1.35122500

C 2.64680800 2.04464600 -4.03769900

H 0.61637600 1.65776100 -3.44618200

C 4.17373800 2.65489000 -2.27444800

H 3.32659100 2.75504500 -0.29447000

C 3.93041700 2.39787700 -3.62150800

H 2.45487100 1.82933600 -5.08682900

H 5.17411700 2.92369600 -1.94201000

H 4.73977400 2.46345200 -4.34531600

H 1.12920600 2.03794000 0.28204900

C 1.74329200 -2.55119100 -3.41774500

C 2.49032900 -3.66723500 -0.89308000

C 3.37206600 -1.03403400 -1.63748700

H 1.47396500 -1.65606600 -3.99017400

H 2.68937600 -2.95508800 -3.79904700

H 0.95174700 -3.29794200 -3.54321500

H 2.79525500 -3.48647600 0.14303500

H 1.68373500 -4.40825600 -0.89880600

H 3.34694800 -4.05766600 -1.45647200

H 3.20992200 -0.20589900 -2.33721900

H 3.53414500 -0.62276100 -0.63491100

H 4.25686400 -1.60274400 -1.94971500

O -3.85271300 -0.18385600 2.99974600

H -2.94708600 -0.12661700 3.37438200

H -4.45424300 -0.29434900 3.75006100

O -2.61458200 -2.73742600 2.39964100

H -3.00577200 -3.35040100 3.03437500

H -3.23475000 -1.98935000 2.35208800

Electronic Energy (0K) = -1950.4717639

Electronic Energy (0K) + ZPE = -1949.930691

Enthalpy (298K) = -1949.891283

Free Energy (298K) = -1950.002012

**TS_D-3W-TA_**

Number of imaginary frequencies: 1

C 0.29568600 -1.05544100 3.00423800

C -0.62046300 -2.10816200 2.83651900

C -0.22586500 -3.38896900 2.43220900

C 1.12607700 -3.56082800 2.21128600

C 2.05786400 -2.50705400 2.37659800

C 1.66575200 -1.24327600 2.76812700

H -0.94060900 -4.19701200 2.30933700

H 1.49209800 -4.53789300 1.90418300

H 3.11133600 -2.70749700 2.19278300

H 2.37619600 -0.43105200 2.89953300

H -2.76754200 -1.91049500 3.09548800

N -1.67887200 -0.26408000 3.46507000

N -0.41964800 0.04746900 3.39481100

N -1.82044700 -1.54084500 3.14366700

O -3.18647500 2.10559800 2.60928300

H -1.99436900 2.37239100 3.14439900

H -3.57895500 1.22442700 2.84107500

O -0.98511700 2.53368300 3.56163300

H -0.52275600 1.60438000 3.58458600

H -1.02323400 2.91422100 4.45505200

C -1.30469400 2.02276200 0.35952800

O -2.27111600 3.02299600 0.73438500

C -3.38727700 2.36872900 1.19695100

O -3.45700800 1.14401300 0.53297500

C -4.64135500 3.16169600 1.01173800

H -4.55803500 4.10975400 1.54957000

H -5.48999100 2.59843400 1.41297300

H -4.80241300 3.35537200 -0.05193200

C -2.15041700 0.77225900 0.19203700

C -1.80549100 -0.45299300 -0.19499200

H -2.62199900 -1.18353900 -0.18873800

Au 0.12075300 -0.95452600 -0.77875400

P 2.30919300 -1.48224400 -1.51235300

H -0.57432600 1.88790600 1.17488500

C -0.58730100 2.41716100 -0.87983100

H -1.21472400 2.62730800 -1.74868200

C 0.74925800 2.40036300 -0.95736000

C 1.54985200 2.58686900 -2.16825400

C 2.91196200 2.89593000 -2.05010100

C 1.00830200 2.42886500 -3.45291300

C 3.70659600 3.06373700 -3.17906300

H 3.34300000 3.01209600 -1.05563900

C 1.80165700 2.60082900 -4.58057000

H -0.03891200 2.15182400 -3.56659000

C 3.15274600 2.92007500 -4.44861300

H 4.76089300 3.30747900 -3.06723500

H 1.36660700 2.47390000 -5.56963800

H 3.77248500 3.04740800 -5.33337200

H 1.31616000 2.20999300 -0.03978100

C 2.62229700 -1.08944400 -3.26606400

C 2.80087600 -3.23371600 -1.37585600

C 3.64003100 -0.59111500 -0.63786600

H 2.43930300 -0.02233000 -3.44181100

H 3.65915500 -1.33111000 -3.53077800

H 1.94099400 -1.66863300 -3.89860900

H 2.76562400 -3.54729600 -0.32673100

H 2.10702000 -3.85587800 -1.95118400

H 3.81853400 -3.37126500 -1.76173100

H 3.51405500 0.48793100 -0.78824600

H 3.59021500 -0.80617200 0.43550800

H 4.61958200 -0.89657300 -1.02626100

O -4.44901000 -0.34957800 2.67825100

H -4.46812000 -0.30523900 1.70994500

H -5.37322800 -0.41686600 2.95239100

Electronic Energy (0K) = -1874.0570814

Electronic Energy (0K) + ZPE = -1873.544924

Enthalpy (298K) = -1873.507836

Free Energy (298K) = -1873.614758

**D-3W-TA**

Number of imaginary frequencies: 0

C 0.30860600 -1.08173700 3.03432100

C -0.69937400 -2.04529000 2.86875800

C -0.41333500 -3.36212500 2.49279300

C 0.92151300 -3.65278900 2.30266100

C 1.93938700 -2.68242400 2.47549700

C 1.66165100 -1.38135000 2.83917900

H -1.19635800 -4.10252600 2.36544500

H 1.20771100 -4.66079500 2.01338600

H 2.97386400 -2.97893100 2.31885200

H 2.43645100 -0.63345500 2.97595800

H -2.84877700 -1.63810000 3.04799300

N -1.65003500 -0.11641000 3.42012100

N -0.36048100 0.06062900 3.37409100

N -1.85881200 -1.36981000 3.13022500

O -3.22879900 2.20123900 2.49918000

H -1.53309400 2.66027500 3.17671500

H -3.64104200 1.38593700 2.84324000

O -0.59947800 2.71197900 3.46284800

H -0.06765700 1.05240200 3.52236800

H -0.59873700 3.23512900 4.27312400

C -1.40169300 2.03300600 0.29059500

O -2.45490600 2.97901000 0.52522200

C -3.49336900 2.28921200 1.11984300

O -3.46390700 0.98581300 0.55898800

C -4.81907900 2.93411000 0.84722700

H -4.82746400 3.94001500 1.27606200

H -5.61394200 2.34497900 1.31703600

H -4.99934400 2.99321300 -0.22948200

C -2.14077700 0.70571800 0.22554800

C -1.69719800 -0.51379000 -0.07759100

H -2.45323500 -1.30433400 -0.00128400

Au 0.24116000 -0.93437400 -0.67622000

P 2.43057100 -1.42489600 -1.42967400

H -0.69699300 2.03877000 1.13956600

C -0.67081900 2.36158300 -0.96058000

H -1.28285100 2.45120900 -1.86073100

C 0.66507900 2.44174100 -1.00134800

C 1.49407300 2.59569900 -2.19764400

C 0.99940600 2.36524200 -3.49038700

C 2.84128100 2.95496400 -2.05270100

C 1.82224400 2.51675400 -4.59966900

H -0.03448600 2.05014300 -3.62509500

C 3.66626800 3.10060900 -3.16300100

H 3.23657500 3.12820700 -1.05166100

C 3.15775200 2.88627600 -4.44132600

H 1.42247900 2.33545100 -5.59517000

H 4.70811800 3.38385900 -3.02946900

H 3.80033500 2.99804700 -5.31176100

H 1.21319600 2.36990600 -0.05618300

C 2.72036800 -1.06078300 -3.19330000

C 2.94209100 -3.16887000 -1.27020000

C 3.75789100 -0.50201800 -0.58382600

H 2.53482400 0.00299300 -3.38506500

H 3.75377200 -1.30681600 -3.46734100

H 2.03088800 -1.65053600 -3.80696400

H 2.90489200 -3.47298800 -0.21827800

H 2.25663700 -3.80366200 -1.84205200

H 3.96251000 -3.30114900 -1.65056100

H 3.59687200 0.57394600 -0.72383000

H 3.74134900 -0.72416100 0.48899800

H 4.73623100 -0.77728500 -0.99692000

O -4.40649800 -0.49065200 2.69624200

H -4.34308800 -0.30556700 1.74389900

H -5.32655800 -0.73196900 2.86316700

Electronic Energy (0K) = -1874.0704833

Electronic Energy (0K) + ZPE = -1873.553023

Enthalpy (298K) = -1873.515070

Free Energy (298K) = -1873.623266

**TS_E-3W-TA_**

Number of imaginary frequencies: 1

C 0.95527000 0.53864600 3.70700900

C 0.93937000 -0.43276300 2.69329500

C 2.11723200 -0.87261500 2.07383800

C 3.28737300 -0.29069900 2.51433700

C 3.29995600 0.68472400 3.54174100

C 2.14567800 1.12011100 4.15984300

H 2.09620400 -1.62446000 1.28731200

H 4.23256500 -0.58469900 2.06390200

H 4.25454800 1.10341100 3.84991900

H 2.15494900 1.86734700 4.94713600

H -0.93414000 -1.04605500 1.44720000

N -1.14352200 -0.02590000 3.26325700

N -0.36399600 0.72366200 4.01080300

N -0.38023600 -0.74272100 2.48811700

O -4.00955900 1.91003600 2.16689400

H -2.84582400 2.30744600 3.64037900

H -4.74899100 1.33091700 2.47237300

O -2.33971700 2.52146800 4.44536900

H -0.86268300 1.46557300 4.53667500

H -2.17393600 3.46955000 4.38861000

C -1.72645100 1.56761100 0.52850100

O -2.81607100 2.47261600 0.34094300

C -3.94401800 1.81601500 0.78769300

O -3.72697600 0.42504100 0.43711000

C -5.18080200 2.30991500 0.09888700

H -5.32303200 3.36806600 0.33593300

H -6.05211300 1.74996300 0.45214400

H -5.08767900 2.18789100 -0.98343200

C -2.39927000 0.21540000 0.34685300

C -1.82551000 -1.00560800 0.21646000

H -2.55427800 -1.82328300 0.20357400

Au -0.02412400 -1.30605300 -0.86232300

P 1.89873200 -1.76040900 -2.13445800

H -1.37242000 1.64008400 1.57202800

C -0.60208700 1.86649700 -0.39395800

H -0.83931900 1.87161300 -1.45949300

C 0.63999900 2.06395500 0.06821500

C 1.85925400 2.27367800 -0.71110100

C 1.87345300 2.31347500 -2.11354100

C 3.07365100 2.42041900 -0.02652700

C 3.06558800 2.49638700 -2.80262200

H 0.94406300 2.20116800 -2.67085900

C 4.26745200 2.60180800 -0.71701400

H 3.07105400 2.38770000 1.06380300

C 4.26702300 2.64025800 -2.10857500

H 3.05845000 2.52586000 -3.89051700

H 5.19918700 2.71435000 -0.16698800

H 5.19806700 2.78262600 -2.65250200

H 0.78830200 2.03802300 1.15351500

C 1.88225700 -1.05806400 -3.81577100

C 2.19529100 -3.53655800 -2.41594800

C 3.46518300 -1.17590200 -1.40497000

H 1.80758100 0.03326700 -3.76278800

H 2.80129200 -1.33440000 -4.34707100

H 1.01548000 -1.44481900 -4.36223700

H 2.29733700 -4.04958800 -1.45368300

H 1.34687200 -3.96935300 -2.95643000

H 3.11117700 -3.67758000 -3.00299600

H 3.38774600 -0.11902000 -1.12350600

H 3.68332900 -1.76587500 -0.50720700

H 4.28341900 -1.30458900 -2.12466500

O -5.97540200 0.16153000 2.79667800

H -5.88101000 -0.56840600 2.17063900

H -6.89239400 0.45093800 2.70608500

Electronic Energy (0K) = -1874.0535374

Electronic Energy (0K) + ZPE = -1873.542826

Enthalpy (298K) = -1873.504565

Free Energy (298K) = -1873.614561

**E-3W-TA**

Number of imaginary frequencies: 0

C 1.30223700 1.49243200 4.11991600

C 1.88260200 0.24357400 3.83525100

C 3.27168600 0.06037300 3.91809400

C 4.02924600 1.15476800 4.28121900

C 3.43334900 2.40783700 4.56156000

C 2.06809000 2.60633100 4.48890000

H 3.72116900 -0.90490300 3.69939000

H 5.10998400 1.06054400 4.35589000

H 4.07412000 3.23998300 4.84426100

H 1.61387100 3.56886700 4.70602800

H -0.21438000 -1.40888400 1.13174100

N -0.24497400 0.01205500 3.52292000

N -0.02553000 1.28000800 3.91650500

N 0.88167900 -0.62565900 3.47374300

O -4.09163100 1.32774400 1.50120500

H -3.00706200 2.20281900 2.79820400

H -4.88306200 0.76917900 1.71139100

O -2.36483600 2.72488100 3.31047300

H -0.82869700 1.92758900 3.89791800

H -2.20755000 3.50753800 2.76828400

C -1.42275800 1.05418800 0.52508400

O -2.52642800 1.76196500 -0.04612000

C -3.66764700 1.04645900 0.23030400

O -3.21030300 -0.36859400 0.22565800

C -4.71550000 1.20755800 -0.82592900

H -5.03359700 2.25353800 -0.85196400

H -5.58115900 0.58243600 -0.58755800

H -4.31940400 0.92236300 -1.80378500

C -1.91674100 -0.37594600 0.45865800

C -1.17839300 -1.52686900 0.63160100

H -1.72255600 -2.45505700 0.80433800

Au -0.41380200 -1.61085800 -1.45188500

P 0.54952300 -1.80365400 -3.56942800

H -1.30598700 1.31313400 1.59070300

C -0.16537800 1.32528500 -0.21622700

H -0.22238600 1.22512800 -1.30302400

C 0.97766600 1.59680300 0.42572700

C 2.30804700 1.74021600 -0.15879300

C 2.59351100 1.41984100 -1.49480900

C 3.35022300 2.19260600 0.66079400

C 3.88082800 1.56635500 -1.99409600

H 1.80352100 1.04396200 -2.14606900

C 4.63957900 2.33487000 0.16135400

H 3.13536600 2.43617500 1.70247800

C 4.90830800 2.02421700 -1.16871800

H 4.08724400 1.31592700 -3.03301900

H 5.43615600 2.68603900 0.81400700

H 5.91647200 2.13148300 -1.56256700

H 0.93632500 1.72041800 1.51083100

C 0.26681300 -0.34423000 -4.61878500

C -0.04864700 -3.20627500 -4.55956000

C 2.35666000 -2.00446700 -3.52846400

H 0.67626500 0.54867500 -4.13315600

H 0.75541600 -0.48556900 -5.59068300

H -0.80865000 -0.20288000 -4.76854100

H 0.15705100 -4.14303100 -4.03166800

H -1.12879100 -3.11522500 -4.71305200

H 0.45886400 -3.21611800 -5.53180300

H 2.81201300 -1.15825600 -3.00163100

H 2.61096000 -2.92745500 -2.99713800

H 2.74736400 -2.05308400 -4.55222400

O -6.12247200 -0.35221800 1.95093400

H -5.90116400 -1.24735900 1.66216100

H -6.98925500 -0.16814300 1.56646200

Electronic Energy (0K) = -1874.0902553

Electronic Energy (0K) + ZPE = -1873.574963

Enthalpy (298K) = -1873.535771

Free Energy (298K) = -1873.650875

**TS_F-3W-TA_**

Number of imaginary frequencies: 1

C 1.69953700 1.08145300 3.89682300

C 2.36115800 0.03418800 3.23210200

C 3.76176000 0.00478900 3.15154600

C 4.44699100 1.04471000 3.74532100

C 3.76910000 2.09650900 4.40668300

C 2.39131200 2.14090700 4.49870400

H 4.27410500 -0.80526800 2.63875200

H 5.53354900 1.06447900 3.70719800

H 4.35504100 2.89353800 4.85872900

H 1.87421200 2.94811900 5.00941800

H -0.79559800 -1.35167400 1.12559700

N 0.24456200 -0.36877900 3.04817600

N 0.38424500 0.76868900 3.75152200

N 1.41405200 -0.82480800 2.72822300

O -4.32197800 1.40082700 1.51297700

H -2.89729400 1.11896700 3.63752100

H -5.30372000 1.24612500 1.72300500

O -2.23960500 1.78580600 3.87104000

H -0.47349500 1.27601400 4.00318700

H -2.52275900 2.58369900 3.40744300

C -1.84813400 1.01310900 0.42402300

O -2.88431000 1.88409600 -0.10252500

C -4.09712800 1.57602000 0.25464800

O -3.60191400 -0.44881500 -0.20306800

C -5.18884300 1.85856300 -0.69657600

H -5.57854600 2.86499400 -0.49838500

H -6.00335300 1.14255400 -0.55809500

H -4.81935300 1.80811300 -1.72098300

C -2.41148200 -0.40785200 0.15102900

C -1.54047400 -1.52423300 0.34086200

H -2.08432200 -2.46572100 0.45669300

Au -0.30241800 -1.66541200 -1.42015800

P 1.10138200 -1.96879700 -3.28062500

H -1.74217800 1.15265500 1.51131400

C -0.58643300 1.33440900 -0.28374900

H -0.61960000 1.24678500 -1.37179700

C 0.53253600 1.67083500 0.37041800

C 1.84897400 1.90732500 -0.21656800

C 2.14525100 1.61989800 -1.55747100

C 2.86452700 2.42685200 0.59630000

C 3.41319000 1.86346700 -2.06846900

H 1.37761500 1.18997600 -2.20127400

C 4.13517800 2.66704900 0.08620800

H 2.64462200 2.64009300 1.64344200

C 4.41304400 2.38884000 -1.24916700

H 3.62673200 1.63389200 -3.11124600

H 4.91054800 3.06726400 0.73654800

H 5.40701400 2.57186900 -1.65109800

H 0.48033000 1.77464200 1.45708400

C 0.94918100 -0.73079400 -4.61024400

C 0.83921600 -3.55553400 -4.13729500

C 2.87888100 -1.97357800 -2.88139600

H 1.22196300 0.26191500 -4.23529900

H 1.61486200 -0.99625000 -5.44087600

H -0.08472400 -0.69993800 -4.96965000

H 0.99722800 -4.38135200 -3.43571900

H -0.18802000 -3.60541100 -4.51340200

H 1.53982500 -3.65046500 -4.97600100

H 3.15696200 -1.01963600 -2.41881400

H 3.09117600 -2.78113200 -2.17280600

H 3.46858000 -2.12449200 -3.79404000

O -6.78544000 1.03583100 2.13386300

H -7.16875900 0.17223600 1.92558700

H -7.42509500 1.68926300 1.81810200

Electronic Energy (0K) = -1874.0749073

Electronic Energy (0K) + ZPE = -1873.562851

Enthalpy (298K) = -1873.523071

Free Energy (298K) = -1873.639238

**F-3W-TA**

Number of imaginary frequencies: 0

C 1.37281700 0.42352100 3.65613600

C 1.13057200 1.80695300 3.75324600

C 2.16065200 2.73599600 3.95386500

C 3.43360400 2.21238000 4.05182100

C 3.69098900 0.82204600 3.95536800

C 2.67653500 -0.08959300 3.76020200

H 1.96634700 3.80175400 4.02704500

H 4.27140300 2.88783000 4.20791100

H 4.71719800 0.47389600 4.04124800

H 2.86351300 -1.15771500 3.68854700

H -0.83221000 2.72111900 3.45906300

N -0.74395500 0.69316000 3.39687700

N 0.17940000 -0.21210300 3.43984700

N -0.21218500 1.91057700 3.58807900

O -4.55049200 1.54030800 1.36477100

H -2.99766500 3.82811200 2.98066300

H -3.88541400 0.88278700 2.09783600

O -2.29079000 3.37384800 2.50327700

H -1.02647000 -1.04965400 1.31721900

H -1.99700000 4.00710100 1.83418700

C -2.07304100 1.11850000 0.16631800

O -3.11891500 1.98298100 -0.33669900

C -4.28047800 2.02391000 0.24825600

O -3.62071800 -0.54915100 -0.57107900

C -5.31991800 2.73991900 -0.52379500

H -6.10935800 3.09759700 0.13864900

H -5.75277400 2.02455500 -1.23426000

H -4.88455600 3.56035900 -1.09835200

C -2.53579300 -0.35453500 -0.03211200

C -1.62329200 -1.36991200 0.45401500

H -2.14045100 -2.31744100 0.63811300

Au -0.14130000 -1.69758900 -1.06437400

P 1.55166700 -2.12777900 -2.63930700

H -1.91888300 1.32947600 1.23268100

C -0.83948700 1.44342400 -0.59536000

H -0.92182300 1.36779300 -1.68179500

C 0.32029100 1.72667400 0.01076500

C 1.61901000 1.91553400 -0.63238100

C 1.78678200 1.90934700 -2.02580300

C 2.75009400 2.08693500 0.17605200

C 3.04715000 2.07156700 -2.58546700

H 0.92302200 1.77906400 -2.67729400

C 4.01338700 2.24316900 -0.38462700

H 2.62747700 2.10338900 1.25946600

C 4.16636600 2.23721600 -1.76801000

H 3.16004600 2.06624800 -3.66796600

H 4.87805100 2.37277200 0.26319200

H 5.15197000 2.36171100 -2.21076800

H 0.32901700 1.77904700 1.10293900

C 1.39448300 -1.23231400 -4.21943000

C 1.68279600 -3.87458600 -3.14140300

C 3.22901400 -1.71049300 -2.06070400

H 1.39724500 -0.15270600 -4.03258800

H 2.23073600 -1.48861200 -4.88159600

H 0.45057000 -1.50393300 -4.70376400

H 1.88151600 -4.49581800 -2.26177700

H 0.73949400 -4.19760700 -3.59430300

H 2.49636000 -3.99897300 -3.86668500

H 3.28037000 -0.64278900 -1.81600900

H 3.46061600 -2.29061700 -1.16115200

H 3.96446000 -1.93982200 -2.84175800

O -3.38696600 0.20185100 2.95491500

H -2.42058500 0.38259800 3.16249500

H -3.48602600 -0.74840700 2.78283100

Electronic Energy (0K) = -1874.087493

Electronic Energy (0K) + ZPE = -1873.575022

Enthalpy (298K) = -1873.536049

Free Energy (298K) = -1873.649102

**TS_G-3W-TA_**

Number of imaginary frequencies: 1

C 1.43115900 0.06734500 3.10665400

C 1.23670300 1.40579300 3.49536200

C 2.30709800 2.27974800 3.72992100

C 3.56965000 1.75108700 3.54894800

C 3.77812600 0.40806300 3.14906000

C 2.72363400 -0.45228900 2.92753600

H 2.15120400 3.31029400 4.03456700

H 4.43677400 2.38560900 3.71696900

H 4.79788000 0.05239200 3.02440000

H 2.87309100 -1.48867700 2.63470800

H -0.72983100 2.35035600 3.64260200

N -0.69914600 0.37436400 3.20764000

N 0.20244000 -0.51984500 2.95002400

N -0.11463100 1.53277900 3.54151700

O -4.64168000 1.51759200 1.21685000

H -3.06254900 3.31381900 3.49560000

H -3.85644000 0.36414900 2.24522500

O -2.28922200 3.09176100 2.96157100

H -1.22935000 -0.87508800 1.06928300

H -2.16181100 3.85338800 2.38113500

C -2.26651600 1.41819800 -0.02481600

O -3.30026200 2.33996900 -0.39650400

C -4.44784600 2.27073100 0.27747400

O -3.52407900 -0.19540300 -1.27818200

C -5.45945900 3.22137300 -0.26034500

H -6.33249800 3.25009700 0.39280300

H -5.76189700 2.89107600 -1.26025400

H -5.02743300 4.22116500 -0.36404600

C -2.69191500 -0.01984800 -0.40346300

C -2.07946700 -1.08089000 0.39525700

H -2.36778100 -2.11537300 0.18412700

Au -0.13467300 -1.44890400 -0.80849000

P 1.74773100 -2.05751600 -2.00382200

H -2.08745900 1.49215300 1.05712800

C -1.03917600 1.77973900 -0.78465000

H -1.15618700 1.83516500 -1.86931600

C 0.14981600 1.91690800 -0.18349900

C 1.45071700 2.11303000 -0.82189500

C 1.62745400 2.13206400 -2.21419000

C 2.57690200 2.26348700 -0.00286600

C 2.89171500 2.31109300 -2.76054100

H 0.77110200 2.00602000 -2.87540100

C 3.84375200 2.43564200 -0.54983300

H 2.44698800 2.25361000 1.07950100

C 4.00514400 2.46343300 -1.93200400

H 3.01309300 2.32675800 -3.84171800

H 4.70372800 2.54959700 0.10772200

H 4.99311600 2.59899700 -2.36615600

H 0.17612000 1.85565900 0.90955500

C 1.62844700 -1.73845000 -3.78899900

C 2.08920700 -3.83750600 -1.86461400

C 3.29027400 -1.24127600 -1.49780600

H 1.50200100 -0.66415400 -3.96221900

H 2.54459200 -2.08230400 -4.28486800

H 0.76623200 -2.27043200 -4.20371100

H 2.27759500 -4.09281500 -0.81661200

H 1.22377300 -4.40707300 -2.21853700

H 2.96906400 -4.09368400 -2.46738200

H 3.24679600 -0.18232300 -1.77561300

H 3.42560600 -1.32484600 -0.41490600

H 4.13564500 -1.71487100 -2.01225900

O -3.32836700 -0.39513700 2.59497700

H -2.53448000 -0.01283900 3.04521900

H -2.79574600 -0.84809400 1.54345700

Electronic Energy (0K) = -1874.0713473

Electronic Energy (0K) + ZPE = -1873.562153

Enthalpy (298K) = -1873.523051

Free Energy (298K) = -1873.635234

**G-3W-TA**

Number of imaginary frequencies: 0

C 1.72745600 -0.18920400 2.38952800

C 1.36277900 0.99749400 3.05011500

C 2.31386600 1.91518200 3.51812900

C 3.63542500 1.59360300 3.27923000

C 4.01440300 0.40865400 2.60210900

C 3.07688200 -0.49821000 2.15318900

H 2.02649000 2.82583000 4.03559800

H 4.41440200 2.27165700 3.62011600

H 5.07199100 0.21153200 2.44518800

H 3.35837000 -1.41865500 1.64523500

H -0.71192400 1.66113500 3.31859700

N -0.42926200 -0.16385800 2.45637400

N 0.58141600 -0.86541200 2.04982300

N 0.00469600 0.95635300 3.05897200

O -4.48759500 1.03304100 0.63813300

H -2.82711800 1.63179200 3.15610800

H -3.78898000 0.06835600 2.11160200

O -2.28975100 2.45395900 3.10471200

H -1.56427300 -1.43692100 0.28107300

H -2.60320700 3.01916100 3.82008700

C -2.07464800 1.12844700 -0.37034100

O -2.98304300 2.23046500 -0.51170100

C -4.17150500 2.06947200 0.08489400

O -3.15020100 -0.01531800 -2.15494000

C -5.00515100 3.29856500 0.00832200

H -6.04868600 3.05561900 0.21426300

H -4.90575100 3.78276000 -0.96641300

H -4.64731800 4.00214600 0.76984300

C -2.63387000 -0.12562000 -1.06302700

C -2.46590400 -1.42836200 -0.34530400

H -2.44486600 -2.25668800 -1.05966200

Au 0.69341100 -0.22073700 -1.06233800

P 1.66484000 -2.29182700 -1.52816100

H -1.89639900 0.93978700 0.69725000

C -0.81804600 1.57102300 -1.06130900

H -0.92327700 1.74995500 -2.13411700

C 0.27322600 2.02754700 -0.36481300

C 1.45147000 2.71209300 -0.90301900

C 1.58476600 3.05751800 -2.25686000

C 2.49590100 3.00541700 -0.01707000

C 2.74147100 3.67931600 -2.70347700

H 0.78140900 2.84370200 -2.95970500

C 3.65530500 3.62311900 -0.46921500

H 2.38781200 2.74257500 1.03519800

C 3.78024900 3.95989300 -1.81385200

H 2.83676200 3.94981200 -3.75237200

H 4.45879200 3.84190600 0.23075400

H 4.68451200 4.44593900 -2.17261900

H 0.23567400 1.94999900 0.72645000

C 0.97818700 -3.07771500 -3.01482700

C 1.42617800 -3.50724800 -0.20009200

C 3.45916200 -2.22847600 -1.80624000

H 1.15412200 -2.43792200 -3.88546700

H 1.45950500 -4.05074200 -3.17140300

H -0.10057900 -3.21996900 -2.88988500

H 1.89158300 -3.14689300 0.72351700

H 0.35469700 -3.65128800 -0.02225200

H 1.88034500 -4.46181700 -0.49276000

H 3.67562600 -1.57907300 -2.66053400

H 3.95847600 -1.82555800 -0.91878600

H 3.83366100 -3.23914200 -2.00964600

O -3.32203100 -0.06615500 2.95146800

H -2.44102600 -0.39945000 2.70819600

H -3.33158200 -1.57111300 0.31604000

Electronic Energy (0K) = -1874.1252064

Electronic Energy (0K) + ZPE = -1873.609917

Enthalpy (298K) = -1873.570449

Free Energy (298K) = -1873.683427

**3a**

Number of imaginary frequencies: 0

C 0.77190200 0.31772300 2.58913000

O 1.24972500 0.58281700 3.91085300

C 1.09158000 -0.43124600 4.78134600

O -1.44579300 0.67813000 3.40753300

C 1.59556500 -0.06068600 6.13588500

H 1.00345300 0.77186500 6.53091400

H 2.63451300 0.27852900 6.07505300

H 1.52035300 -0.91576000 6.80929900

C -0.74561500 0.09733000 2.60545500

C -1.25861600 -0.82346500 1.54726100

H -2.34670500 -0.76318700 1.47171900

C 1.05334300 1.52074200 1.75484800

H 0.69800500 2.46525800 2.17184100

C 1.64218000 1.44826400 0.55505700

C 1.90528500 2.55449700 -0.36549000

C 1.45681300 3.86383600 -0.13505700

C 2.64090700 2.29671300 -1.52969800

C 1.74629600 4.87828100 -1.03710900

H 0.87210300 4.08969700 0.75497700

C 2.92994900 3.31273400 -2.43377300

H 2.98987000 1.28203100 -1.71899100

C 2.48435600 4.60824400 -2.18949800

H 1.39083900 5.88819300 -0.84352200

H 3.50375500 3.09160100 -3.33119600

H 2.70674300 5.40620100 -2.89451700

H 1.97861900 0.46687800 0.20865600

O 0.60114800 -1.49399000 4.46821500

H -0.96555000 -1.84756100 1.81459500

H -0.79589300 -0.60142600 0.57792900

H 1.26852000 -0.57349500 2.18028000

Electronic Energy (0K) = -729.019165

Electronic Energy (0K) + ZPE = -728.778566

Enthalpy (298K) = -728.761534

Free Energy (298K) = -728.824603

**TS_3W-TA_**

Number of imaginary frequencies: 1

C 2.07129500 4.42729300 -6.55283400

O 3.03173700 5.47778600 -6.71469100

C 4.18206800 5.18076600 -7.35043100

O 4.40325800 4.11732800 -7.88954100

C 5.13018800 6.32613000 -7.28128100

H 5.54828600 6.35755600 -6.26786800

H 4.61689300 7.27514500 -7.45760900

H 5.94001800 6.18672100 -7.99888300

C 1.84074600 4.21148100 -5.11498700

C 1.21771800 4.07344400 -4.03707500

H 1.41461800 3.84372400 -2.99927200

Au -0.86787600 4.17975500 -4.61421800

H 2.45718200 3.50056600 -6.99603600

C 0.79442800 4.83546900 -7.21754800

H 0.39849900 5.80354400 -6.90497100

C 0.17801000 4.06086800 -8.11864800

C -1.08781000 4.35641200 -8.78791700

C -1.70930200 5.61289600 -8.71767300

C -1.71360700 3.33856600 -9.51929100

C -2.92281300 5.83529400 -9.35514800

H -1.23378500 6.42716000 -8.17308700

C -2.93382800 3.55899600 -10.14917900

H -1.23045500 2.36376500 -9.58601900

C -3.54209700 4.80851300 -10.06909200

H -3.38909600 6.81651200 -9.29770500

H -3.40701800 2.75555800 -10.70930100

H -4.49288300 4.98684900 -10.56625200

H 0.62883900 3.09562000 -8.36622400

P -3.18750300 4.35687500 -4.83741700

C -3.93563200 3.76633400 -6.38578600

C -3.74447500 6.08413600 -4.70872700

C -4.09297000 3.48379100 -3.52312000

H -4.83833400 6.12787500 -4.77774400

H -3.42050500 6.50658200 -3.75198800

H -3.30431400 6.67117000 -5.52250700

H -3.65054000 2.72654100 -6.57709200

H -5.02741100 3.83857400 -6.30432300

H -3.59282300 4.39360000 -7.21633200

H -3.91120600 2.40630500 -3.60100600

H -3.75025800 3.83285400 -2.54348200

H -5.16747100 3.67936400 -3.62541000

C 0.20067000 0.78825800 -5.85676800

C -0.44825500 0.64537100 -4.61924300

C -1.83063800 0.45019600 -4.50946800

C -2.52714600 0.40332100 -5.70061600

C -1.88698700 0.54718500 -6.95648100

C -0.52521500 0.74071200 -7.05801900

H -2.31919400 0.32935900 -3.54667600

H -3.60373200 0.24646600 -5.67814800

H -2.49250700 0.49884200 -7.85903900

H -0.02508500 0.83925900 -8.01882200

H 0.53816600 0.66018900 -2.70763500

N 1.73886400 0.92018700 -4.33966500

N 1.54185200 0.95742300 -5.61848800

N 0.56570300 0.73667200 -3.71968200

O 4.04854300 3.77838200 -4.74934300

H 4.20057200 2.98465200 -5.30870300

H 3.97637900 3.40445800 -3.84867900

O 4.21748200 1.63312400 -6.49732700

H 3.29687700 1.31619900 -6.44263100

H 4.24917400 2.23888400 -7.25293700

O 3.73619400 2.14207300 -2.55634300

H 3.17360700 1.57279800 -3.11098200

H 3.21351900 2.32156600 -1.76645600

Electronic Energy (0K) = -1874.0395752

Electronic Energy (0K) + ZPE = -1873.526852

Enthalpy (298K) = -1873.487298

Free Energy (298K) = -1873.599091

**P_3W-TA_**

Number of imaginary frequencies: 0

C 2.31257900 0.51395300 -0.93394000

O 3.35026700 1.39078000 -1.45373500

C 4.41354100 0.84954700 -2.05517000

O 4.58099100 -0.34412500 -2.22409100

C 5.37336900 1.90551500 -2.49215500

H 5.83563400 2.35682400 -1.60703000

H 4.85108900 2.70213200 -3.02996400

H 6.14989200 1.46793000 -3.12142000

C 2.41955900 0.50201000 0.57255300

C 1.40351600 0.57803200 1.44715700

H 1.72650700 0.47142800 2.49070900

Au -0.63024000 0.74201800 1.09266700

H 2.52152500 -0.49968400 -1.30894800

C 1.02286200 1.00022700 -1.49811000

H 0.72441900 2.01083500 -1.21360700

C 0.29519400 0.26417700 -2.34845700

C -0.98217800 0.62943300 -2.96066100

C -1.56522600 1.89826700 -2.81807800

C -1.66665700 -0.33617300 -3.71171900

C -2.79182200 2.18321600 -3.40459800

H -1.05170600 2.67129500 -2.24687100

C -2.89626100 -0.05136000 -4.29689300

H -1.21819500 -1.32230200 -3.83581300

C -3.46422300 1.21033100 -4.14430500

H -3.22682900 3.17390000 -3.28595400

H -3.40960400 -0.81583400 -4.87616700

H -4.42378900 1.43853600 -4.60280600

H 0.65670900 -0.73687200 -2.59962300

P -2.95980700 1.06793000 0.80966800

C -3.35739900 2.78603400 0.33672500

C -3.96392000 0.81386000 2.31236500

C -3.81891800 0.04723200 -0.43824000

H -5.02197700 1.01220500 2.10069300

H -3.85207100 -0.21844100 2.66190900

H -3.61926200 1.48983200 3.10235000

H -2.88118300 3.02791800 -0.61982800

H -4.44304500 2.91514800 0.24554800

H -2.97245100 3.46911000 1.10190200

H -3.21731900 -0.03487900 -1.35081300

H -3.97448400 -0.95698300 -0.02841100

H -4.79443000 0.48825700 -0.67897700

C 0.48558800 -2.89601200 0.25382700

C -0.33254200 -2.84748500 1.39230300

C -1.72448000 -2.97133700 1.31929900

C -2.23791900 -3.13668700 0.05098600

C -1.41603200 -3.16829400 -1.10374300

C -0.04440900 -3.05637600 -1.03284200

H -2.34835100 -2.94665700 2.20723800

H -3.31152300 -3.25162100 -0.07432000

H -1.88908300 -3.29689300 -2.07404200

H 0.59372000 -3.09549900 -1.91080500

H 0.34306700 -2.56990400 3.41858700

N 1.78520800 -2.59356400 2.02162000

N 1.75660400 -2.74181000 0.72743100

N 0.54204400 -2.66483300 2.42366100

O 3.73626400 0.30415700 0.92649200

H 4.27169400 -1.36669000 0.30608100

H 3.78569200 0.09464300 1.88844800

O 4.19775600 -2.21090800 -0.18282600

H 2.68478000 -2.64741900 0.21622000

H 4.31272300 -1.90034400 -1.09779600

O 3.91896500 -0.87322300 3.35418000

H 3.31345100 -1.61296500 3.19419600

H 3.60758200 -0.46496600 4.17177100

Electronic Energy (0K) = -1874.0774928

Electronic Energy (0K) + ZPE = -1873.560668

Enthalpy (298K) = -1873.521733

Free Energy (298K) = -1873.634190

**TS_TA_**

Number of imaginary frequencies: 1

C 2.03695900 3.94893300 -2.17397400

O 3.12772200 4.58591700 -1.49250600

C 3.83406000 3.83139400 -0.61261600

O 3.60751400 2.66394400 -0.40301300

C 4.89865700 4.65194800 0.03466700

H 4.44362500 5.49148000 0.57428700

H 5.56197000 5.07543600 -0.72651200

H 5.47310900 4.03375300 0.72615500

C 0.82948400 4.76399500 -1.96973600

C -0.14639100 5.49888400 -2.28091400

H -0.95393200 5.95746000 -1.72405700

Au -0.13524900 5.75757000 -4.41081100

H 1.88396900 2.94329000 -1.75943900

C 2.34394200 3.87904800 -3.63911800

H 2.71343100 4.80810900 -4.07685200

C 2.14091800 2.77248200 -4.36410200

C 2.34822300 2.63688800 -5.80547700

C 2.91091900 3.65478100 -6.59198000

C 1.95115200 1.44932200 -6.43372700

C 3.06318700 3.48643700 -7.96170200

H 3.24187500 4.58331600 -6.12769100

C 2.10100800 1.28257500 -7.80651900

H 1.51791900 0.65222600 -5.83070000

C 2.65692200 2.30090900 -8.57520100

H 3.50293500 4.28482500 -8.55636900

H 1.78485300 0.35370000 -8.27596500

H 2.77786800 2.17253200 -9.64844400

H 1.75567800 1.88412200 -3.85714500

P -0.33358000 6.09552200 -6.71279000

C -0.58237800 4.56878200 -7.67003400

C 1.10652500 6.90433400 -7.47449700

C -1.74010400 7.15459000 -7.16857700

H 0.95233100 6.99468100 -8.55677700

H 1.23604800 7.90087500 -7.03958000

H 2.00815000 6.31413200 -7.28127300

H -1.50433800 4.07892400 -7.34001900

H -0.65886700 4.81070800 -8.73731000

H 0.26061400 3.88718000 -7.50981100

H -2.67292200 6.69681700 -6.82378400

H -1.63006400 8.13575900 -6.69543700

H -1.77391600 7.27638500 -8.25814900

C 1.86231600 6.64949100 1.01726700

C 1.46265600 5.31235800 1.15556700

C 1.66807100 4.60819400 2.35161000

C 2.28021200 5.19508200 3.46578700

C 2.67055800 6.51081100 3.31597400

C 2.46520800 7.22999600 2.11342900

H 1.70235000 7.18909100 0.08682700

H 2.44013500 4.64681300 4.38924500

H 3.15315400 7.01589800 4.14918600

H 2.79494400 8.26447900 2.06064300

N 1.17055800 3.36970400 2.07764000

H 1.12019700 2.54928200 2.67366100

N 0.87371700 4.45026900 0.26600100

N 0.70703900 3.29706500 0.82591900

Electronic Energy (0K) = -1644.8068202

Electronic Energy (0K) + ZPE = -1644.372375

Enthalpy (298K) = -1644.339947

Free Energy (298K) = -1644.440181

**P_TA_**

Number of imaginary frequencies: 0

C 1.15310300 -1.21764800 1.25081600

O 2.35769800 -0.68491900 1.87158700

C 2.88908200 -1.34982400 2.91887400

O 2.42570000 -2.37029100 3.37650900

C 4.08482600 -0.62768000 3.44727700

H 3.74663400 0.24915700 4.01429500

H 4.72071100 -0.26811500 2.63381200

H 4.64943000 -1.28241400 4.11323900

C 0.00311600 -0.38600900 1.76225900

C -0.87765400 0.38873300 1.12385800

H -1.58304400 0.88744000 1.79753800

Au -1.09406100 0.71155300 -0.91377000

H 1.03450900 -2.25940300 1.58097400

C 1.35299300 -1.15416700 -0.22138700

H 1.67748900 -0.18908200 -0.61497000

C 1.15209900 -2.20913000 -1.02052300

C 1.29245600 -2.23601100 -2.47713200

C 1.81414400 -1.16011700 -3.21288200

C 0.88327500 -3.38138100 -3.17248700

C 1.91575200 -1.23217800 -4.59622600

H 2.15525200 -0.26240300 -2.69794900

C 0.98120900 -3.45169900 -4.55838600

H 0.48345800 -4.22492300 -2.61068400

C 1.49796900 -2.37686700 -5.27579600

H 2.32779200 -0.39054100 -5.14993100

H 0.65653900 -4.35038500 -5.07834800

H 1.58005100 -2.42965100 -6.35906100

H 0.82282700 -3.14708700 -0.56540700

P -1.46616600 1.15158600 -3.20620800

C -1.75450800 -0.32006600 -4.24233400

C -0.12012100 2.04051900 -4.05597500

C -2.92965600 2.19332900 -3.51735300

H -0.38291500 2.20341800 -5.10849300

H 0.04319100 3.00772500 -3.56876500

H 0.80516100 1.45683000 -3.99964400

H -2.62426800 -0.86755900 -3.86398700

H -1.93892300 -0.01743400 -5.28051800

H -0.87813600 -0.97761600 -4.20607400

H -3.82389100 1.69694400 -3.12591600

H -2.81044600 3.15527500 -3.00764400

H -3.05064200 2.36466700 -4.59407400

C 0.93218300 1.80095500 3.91875000

C 0.40927100 0.51721900 4.10322300

C 0.26485400 -0.05943600 5.37039200

C 0.63195000 0.61674700 6.53861700

C 1.14780800 1.88204800 6.35521500

C 1.29564500 2.46387400 5.07155300

H 1.04077800 2.23371700 2.92833700

H 0.51792000 0.16795200 7.51979700

H 1.45333900 2.45792800 7.22459900

H 1.70957600 3.46586800 4.99929200

N -0.25387400 -1.29801200 5.11479200

H -0.51120700 -2.02957400 5.77592000

N -0.03592800 -0.43943800 3.22286900

N -0.42874700 -1.52529900 3.83840000

Electronic Energy (0K) = -1644.8473562

Electronic Energy (0K) + ZPE = -1644.408347

Enthalpy (298K) = -1644.376595

Free Energy (298K) = -1644.473920

**In the presence of TA**

**A*_syn_*_-PMe3_ as the precursor**

**TS*_syn_*_-3W-TA_**

Number of imaginary frequencies: 1

C 2.63892100 3.83797900 -1.04202800

O 3.63521500 4.58029200 -1.77150600

C 3.75372600 5.88982200 -1.47348600

O 3.13125700 6.42792000 -0.58096500

C 4.70015300 6.57341600 -2.39433100

H 5.54628900 5.93110300 -2.65241400

H 5.04360000 7.51090700 -1.95236000

H 4.15225000 6.79940600 -3.32059500

C 1.32260200 4.12420200 -1.67476900

C 0.60184100 4.42277800 -2.67900000

H -0.49007500 4.41681700 -2.71576900

Au 1.69018000 4.97202200 -4.40852700

H 2.64194600 4.17261500 0.00263700

C 2.97544000 2.38755900 -1.13420200

H 3.01394100 1.98094300 -2.14659000

C 3.17394500 1.63914100 -0.04201000

C 3.46846100 0.20777200 0.00428100

C 3.50128400 -0.60115600 -1.14131000

C 3.72950900 -0.38108200 1.24827500

C 3.79339800 -1.95396800 -1.03988800

H 3.29264000 -0.17106600 -2.11920800

C 4.02245300 -1.73637700 1.34931200

H 3.70284800 0.23989100 2.14296700

C 4.05567300 -2.52687700 0.20463700

H 3.81418200 -2.56913200 -1.93683200

H 4.22369200 -2.17577700 2.32379400

H 4.28254300 -3.58805300 0.27934800

H 3.11874300 2.13292000 0.93260900

P 2.80620200 5.46354500 -6.41243000

C 3.51605600 3.97517200 -7.18314600

C 4.21259800 6.61224100 -6.28166000

C 1.75301100 6.18208000 -7.71162000

H 4.65066500 6.76629400 -7.27563000

H 3.87742300 7.57569300 -5.88081800

H 4.97231100 6.19035600 -5.61470200

H 2.71747900 3.25617600 -7.39372100

H 4.02294400 4.24257400 -8.11836900

H 4.23465500 3.51426000 -6.49740000

H 0.89860500 5.52374000 -7.90003400

H 1.38272600 7.16227600 -7.39174300

H 2.33488800 6.29747800 -8.63427000

O 0.15273600 3.75293100 -0.12676300

H 0.46557600 3.03939300 0.45251600

H 0.28533300 4.63257600 0.35163700

O 0.63686100 6.15883200 0.73865300

H 0.18393400 6.68254800 0.04373000

H 1.57350400 6.27077900 0.50229400

C 1.65460700 8.58083000 -2.60551700

C 0.38921100 7.97427500 -2.64044800

C -0.33514400 7.87979700 -3.84000400

C 0.15165700 8.38444300 -5.05263000

C 1.39652700 8.98098300 -5.00054400

C 2.13998200 9.07510400 -3.79848300

H 2.21728400 8.64605400 -1.67763500

H -0.41996200 8.31640000 -5.97399500

H 1.82222100 9.39650100 -5.91191900

H 3.11562400 9.55585700 -3.82302100

N -1.47128800 7.23038600 -3.47209400

H -2.27425400 6.96404900 -4.03342600

N -0.35168100 7.38040200 -1.64943800

N -1.45702500 6.94477500 -2.16313800

O -2.35105700 4.18054300 -1.60078100

H -1.68047800 3.88735000 -0.96697800

H -2.29031600 5.14751900 -1.56920000

Electronic Energy (0K) = -1874.0337554

Electronic Energy (0K) + ZPE = -1873.520889

Enthalpy (298K) = -1873.481728

Free Energy (298K) = -1873.594216

**P*_syn_*-_3W-TA_**

Number of imaginary frequencies: 0

C 0.81524900 -1.03425300 2.06747100

O 1.76257500 -0.36721100 1.22116600

C 1.90445700 0.95219500 1.37040700

O 1.31343400 1.60867200 2.21050800

C 2.90586800 1.52668100 0.42658800

H 3.14728100 0.83777900 -0.38712900

H 3.82182800 1.75250100 0.98550400

H 2.52225400 2.47186700 0.02719000

C -0.61281600 -0.82403700 1.60736800

C -1.07776600 -0.39584800 0.43356100

H -2.17353300 -0.40982700 0.39846800

Au -0.07467600 0.27905400 -1.24373400

H 0.92429600 -0.65041400 3.09437900

C 1.13915000 -2.49202800 2.03214400

H 1.18448500 -2.92851200 1.03149000

C 1.27939400 -3.22974700 3.14121500

C 1.50695200 -4.67392500 3.21298200

C 1.51221200 -5.50032500 2.07950700

C 1.73103300 -5.25791000 4.46637100

C 1.74274300 -6.86350900 2.20083200

H 1.32945400 -5.07438000 1.09458500

C 1.96112800 -6.62391600 4.58791600

H 1.72478500 -4.62453800 5.35286800

C 1.96884400 -7.43137400 3.45462600

H 1.74284900 -7.49058900 1.31176900

H 2.13357300 -7.05837900 5.57022000

H 2.14701000 -8.50060000 3.54541600

H 1.22936500 -2.71785400 4.10711900

P 0.99461200 0.91231400 -3.25914900

C 1.93396500 -0.47167500 -3.98694300

C 2.22239700 2.26115700 -3.21306700

C -0.14344300 1.41565800 -4.59210100

H 2.62858800 2.42224900 -4.21944400

H 1.75379400 3.18681600 -2.86207800

H 3.04102200 1.99805700 -2.53440600

H 1.26322500 -1.31988000 -4.16059200

H 2.39204000 -0.16746700 -4.93610600

H 2.71843400 -0.78360700 -3.28797700

H -0.86632600 0.61381800 -4.77702200

H -0.68919500 2.31601300 -4.28813800

H 0.41543200 1.62230600 -5.51305400

O -1.50111800 -1.13697200 2.64983300

H -1.17137100 -1.90311600 3.14936700

H -1.37643200 0.59016900 3.54932100

O -1.11535100 1.52801500 3.56627700

H -1.80516800 2.15661600 2.24599200

H -0.18401600 1.51583600 3.27105900

C -0.23682600 3.75225200 0.25744200

C -1.50885400 3.16897100 0.32005200

C -2.39785400 3.20090300 -0.76354600

C -2.07604700 3.82461600 -1.97433800

C -0.82641900 4.40454300 -2.02822000

C 0.07604100 4.36877000 -0.93470600

H 0.45236100 3.70403700 1.09609100

H -2.76994600 3.84958600 -2.80847100

H -0.51697200 4.90951400 -2.94025500

H 1.04675400 4.84623700 -1.04592500

N -3.49028900 2.51358400 -0.31689700

H -4.36123900 2.31243600 -0.80565400

N -2.16686800 2.47556100 1.29569400

N -3.34623800 2.08138400 0.91094500

O -4.25779400 -0.78114200 1.68316100

H -3.41555700 -1.02616000 2.09338900

H -4.22542800 0.18302800 1.67164900

Electronic Energy (0K) = -1874.0693681

Electronic Energy (0K) + ZPE = -1873.552446

Enthalpy (298K) = -1873.513647

Free Energy (298K) = -1873.624581

**TS*_syn_*_-TA_**

Number of imaginary frequencies: 1

C 2.31085300 3.98836700 -2.26584000

O 3.45509600 4.63668200 -1.70019700

C 4.26993600 3.87313200 -0.93067900

O 4.11271800 2.68777800 -0.76265700

C 5.34623700 4.71237800 -0.32895000

H 4.90552200 5.35484100 0.44353700

H 5.79650800 5.36595000 -1.08167200

H 6.10613400 4.07421200 0.12465300

C 1.11939900 4.77287700 -1.95704900

C 0.07530100 5.47464000 -2.07684900

H -0.28488500 5.83925700 -3.04046500

Au -1.13245200 6.05156900 -0.43106100

H 2.22264300 2.97197400 -1.85994200

C 2.42013700 3.95116900 -3.76193300

H 2.65080900 4.90128000 -4.24502600

C 2.23034000 2.81384800 -4.44373700

C 2.29823300 2.62992400 -5.89128800

C 2.49873000 3.68883500 -6.78982500

C 2.15698400 1.33333800 -6.40330800

C 2.56094800 3.45038600 -8.15543600

H 2.60282200 4.70643900 -6.41783100

C 2.21894100 1.09522300 -7.77129000

H 1.99909300 0.50708400 -5.71121600

C 2.42203400 2.15387700 -8.65145900

H 2.71620600 4.28061100 -8.84096300

H 2.10829300 0.08179900 -8.15052200

H 2.47015200 1.97231300 -9.72284200

H 2.00941400 1.90814900 -3.87282800

P -2.45026900 6.71483600 1.37763400

C -1.51791500 7.64039800 2.63601400

C -3.19771600 5.33081800 2.29132500

C -3.84737800 7.79434600 0.94326800

H -3.78048900 5.71440500 3.13767700

H -3.85315400 4.75998700 1.62557400

H -2.40784300 4.66857100 2.66272200

H -1.04078600 8.51347600 2.17845400

H -2.19419700 7.96839500 3.43505000

H -0.74047900 6.99413400 3.06115200

H -3.47222600 8.70705100 0.46911900

H -4.50894200 7.27641600 0.24139900

H -4.40820200 8.05686700 1.84858800

C 2.24739400 6.50178200 1.27280900

C 1.67453900 5.22130400 1.29693800

C 1.24952100 4.63207300 2.49753200

C 1.39287900 5.27473500 3.73358700

C 1.96714600 6.53075800 3.69754400

C 2.38336100 7.13982200 2.48879200

H 2.56659900 6.95486700 0.33672600

H 1.07534000 4.81114500 4.66277200

H 2.10486700 7.07429200 4.62913600

H 2.82419500 8.13262400 2.52860100

N 0.74298100 3.43149900 2.09850700

H 0.34861200 2.68011100 2.65584700

N 1.40285800 4.33672900 0.28415500

N 0.84944600 3.27773100 0.77253200

Electronic Energy (0K) = -1644.7987924

Electronic Energy (0K) + ZPE = -1644.364809

Enthalpy (298K) = -1644.332215

Free Energy (298K) = -1644.434244

**P*_syn_*_-TA_**

Number of imaginary frequencies: 0

C 2.00943800 -1.38970000 -1.05121200

O 3.18385200 -0.61913600 -0.66425600

C 3.98911200 -1.11998900 0.29387500

O 3.76780100 -2.15033000 0.89083600

C 5.15868800 -0.21825800 0.51787800

H 4.80967700 0.75325700 0.88576000

H 5.68572800 -0.03816000 -0.42427900

H 5.83677300 -0.66298500 1.24787600

C 0.83157900 -0.82238900 -0.30326100

C -0.26624100 -0.20348500 -0.74422300

H -0.31775600 -0.13190800 -1.83469600

Au -1.72498300 0.62326700 0.47754100

H 2.17793400 -2.43380500 -0.75230400

C 1.88087000 -1.27885600 -2.52966400

H 1.84519300 -0.26426700 -2.93072200

C 1.81890200 -2.35557200 -3.32132400

C 1.68498300 -2.37467300 -4.77868000

C 1.55909300 -1.21027700 -5.55111600

C 1.68483700 -3.61279700 -5.43413000

C 1.44225800 -1.28800600 -6.93201500

H 1.55031200 -0.23476400 -5.06813200

C 1.56715000 -3.69088200 -6.81737700

H 1.77976500 -4.52264100 -4.84231700

C 1.44615300 -2.52766800 -7.57149200

H 1.34575400 -0.37524900 -7.51617800

H 1.56986800 -4.66231600 -7.30697000

H 1.35314000 -2.58381500 -8.65380800

H 1.87413500 -3.34096100 -2.85050900

P -3.24882800 1.62518200 1.97607500

C -2.40516700 2.19535300 3.48912000

C -4.58508200 0.55009200 2.59125300

C -4.11683800 3.10694600 1.36834500

H -5.20476400 1.09591900 3.31317800

H -5.20814400 0.22052200 1.75332500

H -4.15297700 -0.33149000 3.07631300

H -1.65808300 2.95184200 3.22370000

H -3.12659700 2.62839200 4.19283500

H -1.89374400 1.35015500 3.96405300

H -3.38529600 3.86057200 1.05867100

H -4.73652100 2.84272100 0.50512000

H -4.75346600 3.52058000 2.15999200

C 1.62516700 1.37204000 1.92484100

C 1.34558400 0.00632000 2.05053800

C 1.37551000 -0.65143800 3.28562500

C 1.67965500 0.01610800 4.47694600

C 1.94941500 1.36222500 4.35120700

C 1.92623100 2.02732800 3.10007800

H 1.60781200 1.87084900 0.96022400

H 1.70086100 -0.49873100 5.43167000

H 2.19292300 1.93698000 5.24068000

H 2.15495300 3.08919600 3.07104600

N 1.05450200 -1.94472200 2.98121900

H 0.96896600 -2.74421400 3.60703400

N 1.00404200 -0.96277700 1.13883200

N 0.83029700 -2.12695600 1.70557500

Electronic Energy (0K) = -1644.8441135

Electronic Energy (0K) + ZPE = -1644.404849

Enthalpy (298K) = -1644.373049

Free Energy (298K) = -1644.472815

**TS_C-1W-PPh3_**

Number of imaginary frequencies: 1

C 1.22528100 -0.45800800 1.41090800

O 1.25197200 -1.25138700 2.65874900

C 0.71708200 -2.45157400 2.44518300

O -0.07317300 -2.39256300 1.20695500

C 0.01868600 -3.07100200 3.59581100

H -0.81193800 -2.42526400 3.89311600

H 0.71875400 -3.16441000 4.43078100

H -0.35644000 -4.06002500 3.32346600

C 0.01099700 -1.01520700 0.72592900

C -0.87907200 -0.52483600 -0.11808800

H -1.65909100 -1.22166000 -0.43311900

Au -0.79034100 1.42488100 -0.81217200

P -0.64619900 3.65761600 -1.58080100

H 2.15005000 -0.68829400 0.85684700

C 1.17169400 0.98274500 1.75158100

H 0.28158300 1.31236100 2.29034700

C 2.16044700 1.82423500 1.41718600

C 2.20712100 3.26359100 1.66887100

C 1.09422000 3.99340300 2.11418100

C 3.41104200 3.94609800 1.44970200

C 1.18944100 5.36044600 2.33829400

H 0.14071200 3.49087800 2.27154500

C 3.51016200 5.31189400 1.68986800

H 4.27580400 3.39004800 1.08841000

C 2.39954300 6.02357900 2.13511400

H 0.31170900 5.91435600 2.66761800

H 4.45460700 5.82400500 1.51761200

H 2.47175200 7.09409400 2.31425600

H 3.03178300 1.41273500 0.89933800

O 1.65815600 -3.43040100 1.80835000

H 0.87562900 -3.12295900 0.89993200

H 2.56966700 -3.07571000 1.76157800

C 1.09213200 4.19005200 -1.73645100

C 1.54656200 5.47385300 -1.42720300

C 1.99000300 3.24369500 -2.24723100

C 2.88235800 5.80730300 -1.63700900

H 0.86567600 6.21606500 -1.01421100

C 3.32088000 3.58308900 -2.46054700

H 1.63929800 2.23728500 -2.48164200

C 3.76743100 4.86767400 -2.15703200

H 3.23101000 6.80721900 -1.38737600

H 4.01079100 2.84380300 -2.86115600

H 4.80942000 5.13407200 -2.31971000

C -1.51752900 4.84642500 -0.50972500

C -1.72264300 6.17627500 -0.89589000

C -2.00606100 4.40240000 0.72280400

C -2.37879600 7.05507900 -0.04153900

H -1.37333700 6.52485200 -1.86764200

C -2.67144900 5.28245100 1.57180000

H -1.86490300 3.35956400 1.01129300

C -2.85025600 6.60964100 1.19218000

H -2.53068200 8.08892200 -0.34273100

H -3.05127200 4.92971600 2.52795600

H -3.36860000 7.29909100 1.85477800

C -1.37101200 3.90512800 -3.23898100

C -2.59377400 3.27899000 -3.50638400

C -0.76930900 4.69787600 -4.21894300

C -3.21190200 3.45177300 -4.73937100

H -3.06033500 2.65369000 -2.74377400

C -1.38943800 4.86227600 -5.45502000

H 0.18296600 5.18828100 -4.02079000

C -2.60783600 4.24195900 -5.71518800

H -4.16176900 2.96231900 -4.94211600

H -0.91698200 5.47846000 -6.21664900

H -3.08732200 4.37120600 -6.68286300

Electronic Energy (0K) = -1900.3173292

Electronic Energy (0K) + ZPE = -1899.804093

Enthalpy (298K) = -1899.769518

Free Energy (298K) = -1899.873847

**
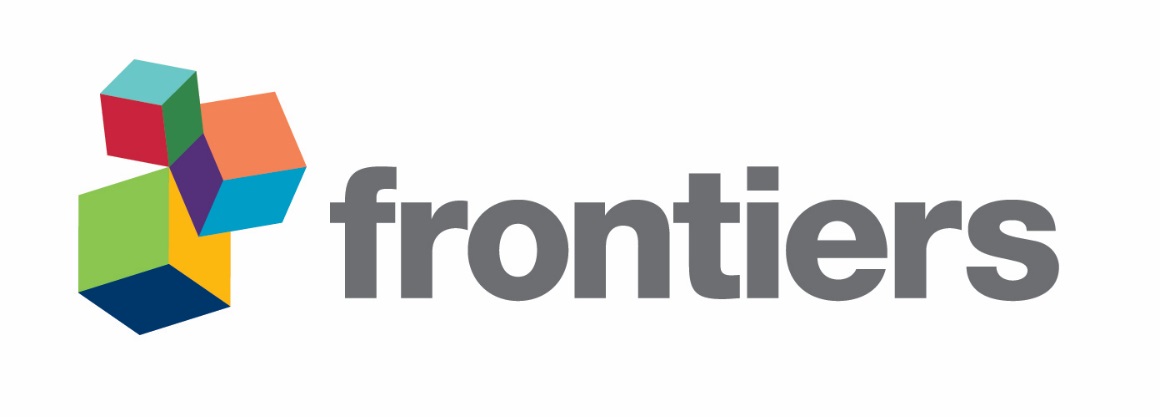
**
